# Mechanistic and Kinetic Investigations of ON/OFF (Photo)Switchable Binding of Carbon Monoxide by Chromium(0), Molybdenum(0) and Tungsten(0) Carbonyl Complexes with a Pyridyl‐Mesoionic Carbene Ligand

**DOI:** 10.1002/chem.202201038

**Published:** 2022-07-13

**Authors:** Pit J. Boden, Patrick Di Martino‐Fumo, Tobias Bens, Sophie T. Steiger, Daniel Marhöfer, Gereon Niedner‐Schatteburg, Biprajit Sarkar

**Affiliations:** ^1^ Department of Chemistry and State Research Center Optimas TU Kaiserslautern Erwin-Schrödinger-Straße 52 67663 Kaiserslautern Germany; ^2^ Chair of Inorganic Coordination Chemistry Institute of Inorganic Chemistry University of Stuttgart Pfaffenwaldring 55 70569 Stuttgart Germany

**Keywords:** group 6 metals, mesoionic carbene, metastable compounds, photochemistry, reversible reactions, time-resolved FTIR spectroscopy

## Abstract

This work tackles the photochemistry of a series of mononuclear Cr^0^, Mo^0^ and W^0^ carbonyl complexes containing a bidentate mesoionic carbene ligand of the 1,2,3‐triazol‐5‐ylidene type. FTIR spectroscopy, combined with density functional theory calculations, revealed a clean photo‐induced reaction in organic solvents (acetonitrile, pyridine, valeronitrile) to give mainly one photoproduct with monosubstitution of a carbonyl ligand for a solvent molecule. The highest photodissociation quantum yields were reached for the Cr^0^ complex under UV irradiation (266 nm). Based on previous investigations, the kinetics of the dark reverse reactions have now been determined, with reaction times of up to several hours in pyridine. Photochemical studies in the solid state (KBr matrix, frozen solution) also showed light‐induced reactivity with stabilization of the metastable intermediate with a free coordination site at very low temperature. The identified reactive species emphasizes a mechanism without ligand–sphere reorganization.

## Graphical abstract

**Shining light on group 6 photochemistry**: The reversible (photo)chemistry of chromium(0), molybdenum(0) and tungsten(0) carbonyl complexes containing a mesoionic carbene ligand has been investigated. Kinetic and mechanistic studies were performed by using (temperature‐dependent) FTIR and UV/VIS spectroscopy, in combination with density functional theory calculations.

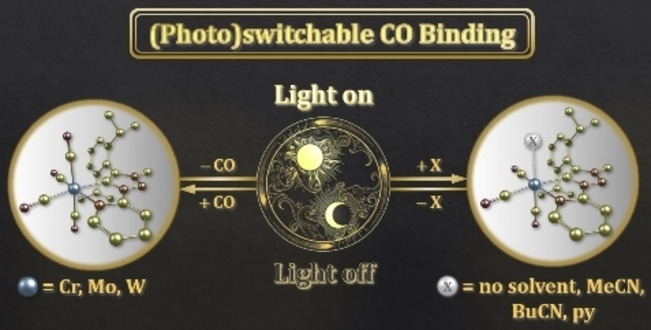

## Introduction

Light‐driven chemical reactivity is an elegant way to generate new classes of chemical substances, and to induce unusual types of catalysis.^[1]^ Oftentimes photo‐induced reactions tend to be very different from their thermal counterparts, as light can be used to selectively access states that might not be accessible in a selective manner in thermal reactions. In this context, carbonyl complexes of zero‐valent group 6 metals are well‐known for their mostly irreversible loss of carbon monoxide (CO) on irradiation with light.[^2^, ^3^, ^4^, ^5^, ^6^, ^7^, ^8^, ^9^, ^10^, ^11^, ^12^, ^13^, ^14^] Such reactions are well established for the parent [M(CO)_6_] (M=Cr, Mo, W) complexes,[^9^, ^10^, ^11^, ^12^, ^13^, ^15^, ^16^] systems of the type [LM(CO)_5_] (L is usually a monodentate N‐donor),[^2^, ^3^, ^17^] but also for complexes of the type [LM(CO)_4_] (M=Cr, Mo, W), where L is usually a chelating ligand containing N‐donors such as 2,2′‐bipyridine.[^2^, ^5^, ^6^, ^7^, ^8^, ^14^, ^18^] In the aforementioned metal complexes, apart from the specific case of W(CO)_6_ in acetonitrile (MeCN),^[9]^ irradiation with UV/VIS light leads to an irreversible loss of CO in solution, and such a method is often used for performing facile substitution reactions on these complexes.

**Figure 1:**
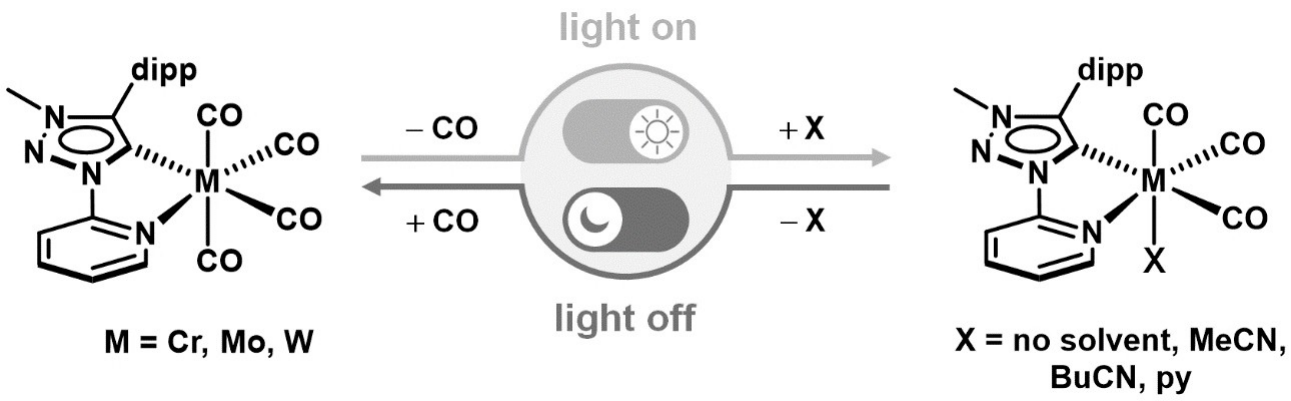
Photochemical reactivity of **Cr**, **Mo**, and **W**.

In recent years, N‐heterocyclic carbenes (NHCs), and in particular mesoionic carbenes (MICs) of the 1,2,3‐triazol‐5‐ylidene type have been established as privileged ligands in the fields of photochemistry and photophysics.[^19^, ^20^, ^21^, ^22^, ^23^, ^24^, ^25^, ^26^] It was shown that MIC containing transition metal complexes often display much more promising excited state properties for applications in comparison to metal complexes that contain only N‐donor ligands. Additionally, MIC ligands were also shown to form luminescent metal complexes with 3d transition metals[^20^, ^21^, ^22^, ^24^, ^26^] as well as with main group metals.[^25^, ^26^] We have recently reported Cr^0^, Mo^0^ and W^0^ carbonyl complexes with a pyridyl‐MIC ligand (**L**; Figure 1).[^19^, ^20^] These compounds display electrochemical, photophysical and photochemical properties that are largely different from related complexes with exclusively N‐donor ligands. The Cr^0^ complex displays a completely reversible oxidation to the corresponding Cr^I^ complex at ambient temperatures.^[19]^ In solution, they display a completely light‐driven reversible bond activation reaction, with an almost complete recovery of the initial species when left in the dark.^[19]^ This observation is very distinct from photochemical reactions observed for other related group 6 carbonyl complexes in which the photo‐induced CO ligand loss is always completely irreversible.[^2^, ^5^, ^6^, ^7^, ^8^, ^14^, ^18^] In the literature, a (photo)switchable coordination of CO by a 3d metal complex is only known for very few Cu^I^ pyridylalkylamine systems so far, to the best of our knowledge.^[27]^

In this contribution, we tackle the question of the reversible photochemical bond activation reaction in the abovementioned complexes of Cr^0^ (**Cr**), Mo^0^ (**Mo**) and W^0^ (**W**; Figure 1) from a mechanistic perspective by using a combination of experimental and theoretical methods. The photochemical reactivity in solution and in the solid state was followed by FTIR spectroscopy, including investigations at low temperature to stabilize reactive intermediates, and interpreted by considering density functional theory (DFT) calculations. Beyond these mechanistic insights, the photodissociation quantum yields and the kinetics of the dark reverse reaction were determined on basis of the evolution of IR spectra over time. To the best of our knowledge, this is the first mechanistic investigation on group 6 metal complexes of the type [LM(CO)_4_] in which the photochemical bond activation reactions are almost completely reversible.

## Results and Discussion

Based on our preliminary studies of the photochemistry of **Cr** and **Mo** in different organic solvents acetonitrile (MeCN), pyridine (py), dichloromethane,^[19]^ we proceeded with refined photochemical studies by FTIR spectroscopy on **Cr**, **Mo** and **W** in MeCN and pyridine. The experimental procedure was optimized by irradiation of the liquid sample cell with two separate laser beams from opposite directions to ensure a homogenous illumination of the cell volume. This procedure enabled in particular the determination of the photodissociation quantum yields and kinetic studies, as presented below.

**Figure 2:**
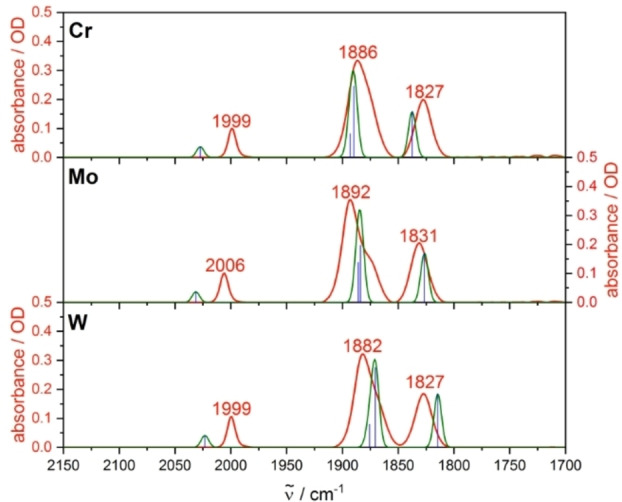
Experimental IR spectra of **Cr**, **Mo**, and **W** in MeCN (red) as well as calculated IR frequencies (blue) and convoluted spectra (green). Calculations: DFT/B3LYP‐D3(BJ)/def2‐TZVP/COSMO, scaling factor: 0.99, Gaussian convolution with FWHM=8 cm^−1^.

Fresh solutions of the complexes **Cr**, **Mo** and **W** show three strong IR absorption bands localized at 1995–2006, 1877–1892 and 1827–1835 cm^−1^, with respect to the metal center and the solvent (Figures 2 and S30).[^19^, ^20^] The experimental IR spectra are very well described by the theoretical spectra calculated by DFT with simulation of solvent effects through the conductor‐like screening model (COSMO). Hereby, the COSMO model is crucial for an accurate description of the CO stretching frequencies. The comparison between experiment and theory also confirmed the assignment of the abovementioned experimental bands to the four CO stretching vibrations.

**Figure 3:**
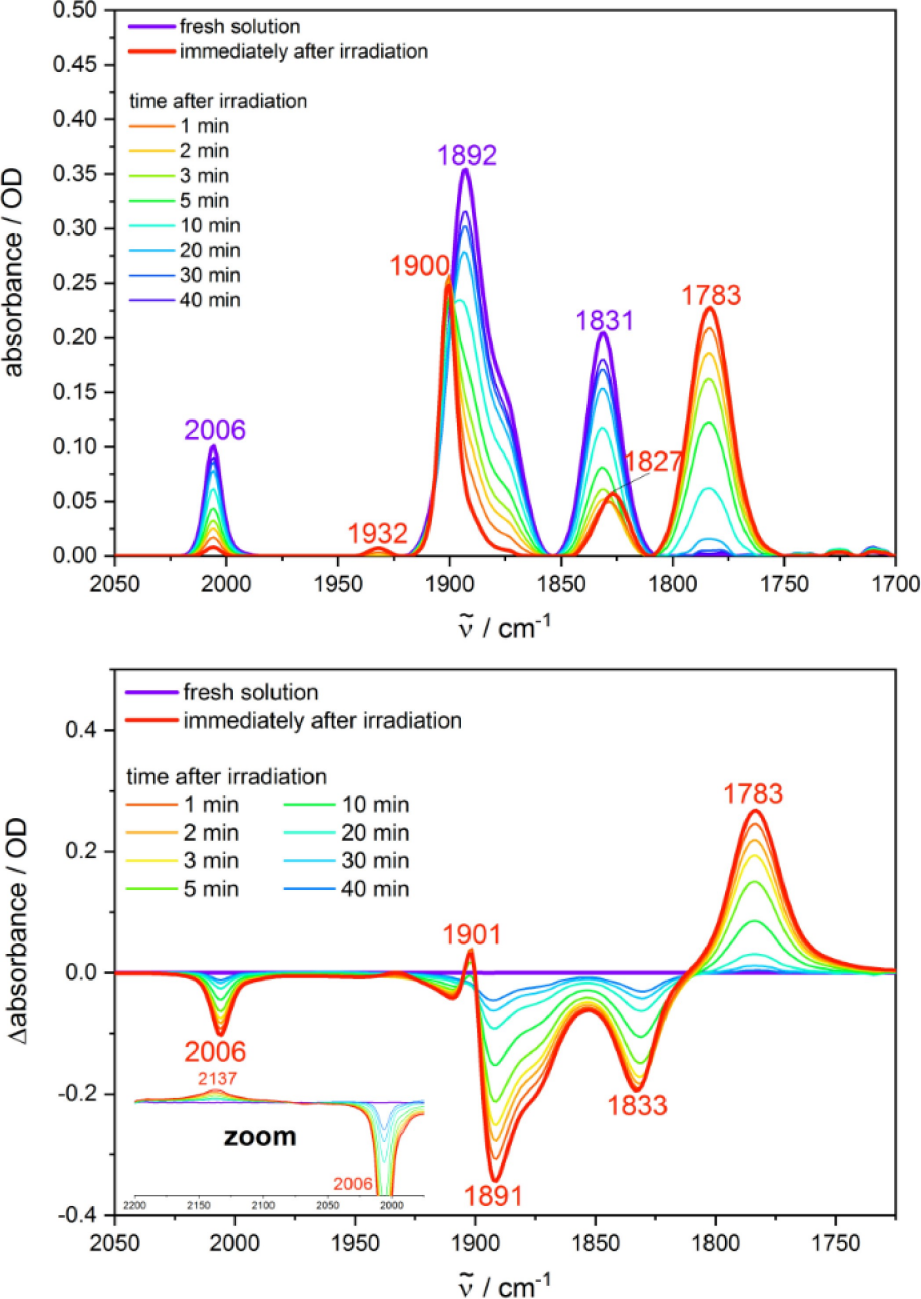
Irradiation (*λ*_ex_=355 nm) of a fresh solution of **Mo** in MeCN (*c*=6 mM), and spectra recorded in the dark after irradiation (top: absolute absorption spectra, bottom: difference spectra).

Irradiation of the respective solutions was performed at 355 nm over 15 or 20 s for **Cr** and 60 s for **Mo** and **W** to stimulate the photochemical reaction. Excitation times were selected to achieve an almost complete conversion to the photoproducts, but prevent eventual follow‐up reactions, which would impede a clear identification of photoproducts and reaction pathways. Experiments were performed at concentrations of 3 and 6 mM to analyze potential concentration effects on the photo‐induced reaction products. In MeCN, intense bands were observed at 1892–1900 and 1779–1783 cm^−1^ upon irradiation independent of concentration, with a slight shift of the spectral frequencies with respect to the metal center (Figures 3 and S1–S5). Only minor traces of side products were observed at 1927 cm^−1^ (**Cr**) and 1932 cm^−1^ (**Mo**, *c*=6 mM), revealing a clean conversion of the starting complex. A red‐shift of 4 cm^−1^ was found for the initial band at about 1830 cm^−1^ in the case of **Cr** and **Mo**, which could result from the same side product. Interestingly, a weak IR signal was observed at 2135–2138 cm^−1^ in the difference spectra of all three complexes (Figures 3 and S1–S5). This feature is in good agreement with the IR spectrum of CO gas dissolved in MeCN, which shows a sharp band at 2138 cm^−1^ (Figure S8). The control experiment clearly points to dissolved free CO in solutions of the three complexes upon irradiation, where the broader band most likely results from solvation effects.

Performing the experiment in pyridine yielded similar observations with weak side bands at 1718 (**Mo**) and 1727 cm^−1^ (**Cr**, only recognizable in the difference spectrum), which are assigned to a minor side product (Figures S9–S14). Analogous to MeCN, a weak broad band was found at 2132–2133 cm^−1^, consistent with the spectrum of free CO gas dissolved in pyridine with a sharp peak at 2133 cm^−1^ (Figure S16).

**Figure 4:**
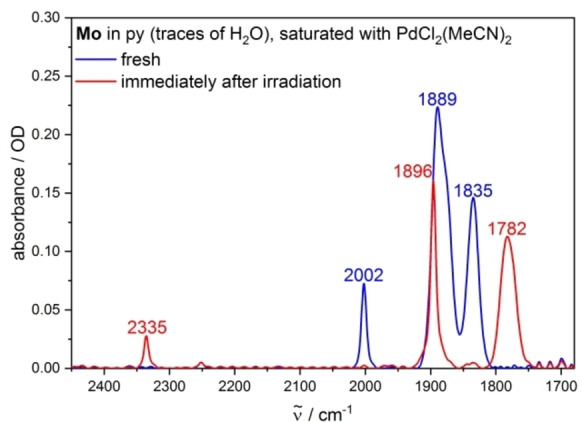
IR spectra of a solution of **Mo** in py (containing traces of H_2_O, *c*=6 mM), saturated with PdCl_2_(MeCN)_2_, before and after irradiation (*λ*_ex_=355 nm).

As the presence or absence of free CO molecules in solutions of the complexes upon irradiation is a key point in the identification of the photochemical reaction, we conducted further analysis with the CO detection reagent PdCl_2_(MeCN)_2_. Pd^II^ is known to oxidize CO to CO_2_ in presence of water,^[28]^ a reaction that only occurs with uncoordinated CO molecules. The formed CO_2_ is an ideal IR probe as it shows an intense IR absorption in a region, where an interference with other absorption bands is not to be expected. Indeed, a sharp and quite intense absorption band was observed at 2335 cm^−1^ in pyridine (containing traces of H_2_O) for all three complexes, which is assigned to CO_2_ and confirms indirectly the formation of free CO molecules (Figures 4, S28, and S29). Unfortunately, the photochemical studies in MeCN under presence of PdCl_2_(MeCN)_2_ failed due to undesired dark reactions taking place before irradiation.

**Figure 5:**
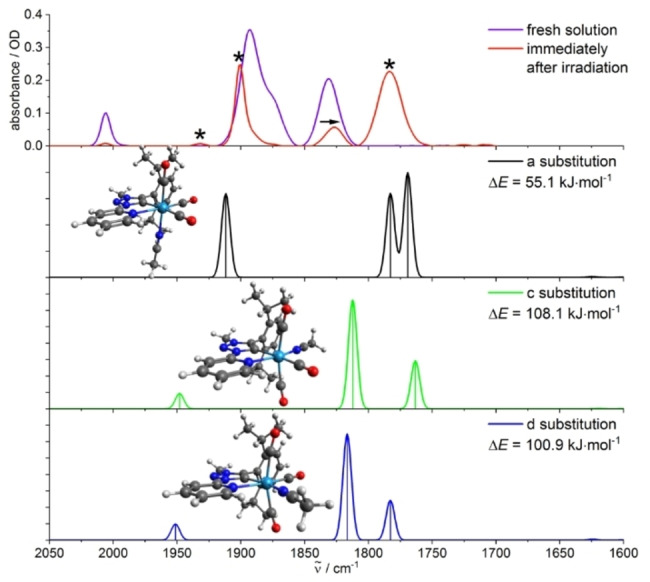
Experimental IR spectra of **Mo** in MeCN before and after irradiation (upper trace, bands marked with asterisks result from the formed photoproduct(s)) and calculated IR spectra of the photoproducts with the substitution of a CO ligand for a MeCN molecule (three lower traces), including the optimized structures and calculated enthalpies of reaction. The substitution occurs in the axial position for isomer a ([LMo(CO)_3_MeCN_ax_]) and in the equatorial position for isomers c/d ([LMo(CO)_3_MeCN_eq_]), respectively. Calculations: DFT/B3LYP−D3(BJ)/def2‐TZVP/COSMO, scaling factor: 0.99, Gaussian convolution with FWHM=8 cm^−1^.

Concerning the irradiation of **Cr**, **Mo**, and **W** in MeCN and pyridine, experimental data suggest the formation of one main photoproduct, next to minor contributions of side products. Hereby, side reactions seem to play a larger role for **Cr**. For a clear identification of the formed photoproducts, in‐depth quantum chemical calculations (including COSMO) were performed for a large series of conceivable photoproducts. Considering the presented experimental results, a substitution of a CO ligand for a solvent molecule plays a key role ([LM(CO)_3_(X)], with X=MeCN or py). Accordingly, all three potential isomeric photoproducts were optimized, followed by harmonic frequency calculations. Interestingly, the isomer with a substitution of an axial CO ligand for a solvent molecule ([LM(CO)_3_(X_ax_)]) is in very good agreement with the experimental data independent of the metal center or the solvent. The theoretical spectra of the two equatorially substituted isomers of the remaining two isomers ([LM(CO)_3_(X_eq_)]) give only a limited description of the experiment (Figures 5 and S31–S35), but could be responsible for the small side bands observed experimentally in MeCN at 1927 (**Cr**) and 1932 cm^−1^ (**Mo**) as well as the abovementioned red‐shift of 4 cm^−1^ for the band at approx. 1830 cm^−1^.

**Figure 6:**
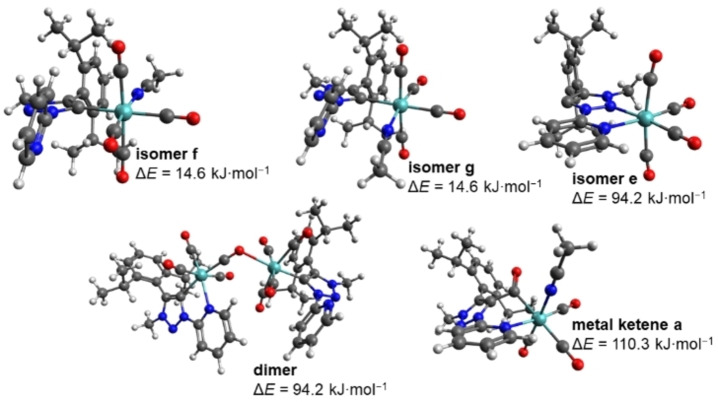
Optimized structures and enthalpies of reaction of selected photoproducts of **Mo** in MeCN. Calculations: DFT/B3LYP−D3(BJ)/def2‐TZVP/COSMO.

At the same time, the axial monosubstitution was throughout the photoproduct with the smallest predicted enthalpy of reaction with values of 31.7–66.5 kJ mol^−1^, depending on the metal center and the solvent (Figures 5 and S31–S35). Hereby, the values are higher in MeCN (55.1–66.5 kJ mol^−1^) compared to pyridine (31.7–43.6 kJ mol^−1^) and follow the trend **Cr**>**W**>**Mo**. At first sight, these values might appear rather high, but the reaction enthalpies are far below the energy input resulting from the absorbed 355 nm photon in the experiment (337 kJ mol^−1^). Thus, [LM(CO)_3_(X_ax_)] can be assigned as main product from an energetic point of view, even though the isomers [LM(CO)_3_(X_eq_)] could also contribute to a small extent. A bisubstitution with exchange of two CO ligands for solvent molecules ([LM(CO)_2_(X)_2_]) is not in good agreement with the experimental IR spectra at least for three of the four isomers, as their theoretical spectra show two strong bands in the region of 1825–1675 cm^−1^ versus only one in the experiment (Figures S36–S41). Only the fourth isomer [LM(CO)_2_(X_eq_)_2_] shows one single calculated band in the mentioned spectral region (1825–1675 cm^−1^) that might be assigned to the experiment. However, it is energetically the most unfavorable bisubstitution (≥ 207.6 kJ mol^−1^). Concerning the weak side bands observed experimentally in py at 1718 and 1727 cm^−1^ for **Mo** (Figures S10 and S13) and **Cr** (Figures S9 and S12), respectively, an assignment to [LM(CO)_2_(py_ax_)(py_eq_)] is conceivable (Figures S39 and S40). However, the calculated spectra of [LM(CO)_2_(py_ax_)_2_] and [LM(CO)_2_(py_ax_)(py_eq_)] are similar, making an unambiguous assignment difficult from that point of view. It can only be mentioned that the formation of [LM(CO)_2_(py_ax_)(py_eq_)] gives the smallest enthalpy of reaction. A trisubstitution is excluded as the predicted bands are too far red‐shifted with respect to the experiment and the reaction enthalpies are much higher (≥ 228.0 kJ mol^−1^; Figures S42–S47). Next, the formation of a metallaketene upon irradiation was considered, whereby a CO ligand is inserted into the initial metal–carbene bond and the vacant coordination site is occupied by a solvent molecule (Figure 6). A related photochemical reactivity has been reported for a Fischer‐carbene complex of Cr^0^ with conversion to a metal‐ketene upon visible light excitation.^[29]^ This pathway occurs without a loss of CO ligands, inconsistent with the free CO observed experimentally. However, the calculated IR spectrum of the metallaketene with insertion of a CO ligand into the metal–carbene bond of the starting species and a solvent molecule coordinated in axial position (metal ketenes a or d, Figures S48–S53) is in good agreement with the experimental new absorption bands, so that a contribution of this reaction cannot be fully excluded at the current state of investigation. Nevertheless, the higher reaction enthalpies of ≥ 90.8 kJ mol^−1^ compared to the monosubstitution (≥ 31.7 kJ mol^−1^) suggest that the formation of a metallaketene most likely takes place at most as side reaction and is not the main reaction product. Beyond this, a photoproduct with cleavage of the initial metal–carbene bond, followed by a rotation around the C−N bond bridging the triazole and pyridine rings and coordination of the triazole via the free nitrogen to the metal center was considered in the calculations (isomer e in Figures 6 and S54–S59).

The calculated IR spectra of this structure are only partly in agreement with the experiment and do not explain the strong product band at 1778–1784 cm^−1^, so that this species could only occur as side product. Furthermore, it is unlikely that this photoproduct with an uncoordinated carbene is stable in solution over a longer period at ambient temperature under experimental conditions (e. g., protonation of the carbene), which is inconsistent with the slow dark reverse reaction described in more detail in the following section. This drawback is in principle circumvented by isomers f and g with cleavage of the metal–pyridyl bond instead of the coordination of the carbene to the metal center (Figures 6 and S54–S59). The resulting vacant coordination site at the metal is occupied by a solvent molecule. Herewith, the two isomeric structures only differ by the position of the coordinated solvent molecule relative to the now monodentate pyridyl‐MIC ligand. The calculated IR spectra show similar bands, which could contribute to the experimental IR spectrum, but do not explain the new band observed experimentally at 1778–1784 cm^−1^ upon irradiation. The low reaction enthalpies of 4.0–35.9 kJ mol^−1^ suggest the two isomers as conceivable side products, but can be excluded as main photoproducts. A similar description is valid for a dimeric structure formed out of two complex molecules with a CO ligand bridging the two metal centers and loss of one CO molecule per formed dimer (Figures 6 and S54–S59). Hereby, it should be mentioned that no optimized minimum structures were found for other conceivable dimers. However, dimerization would require a collision between one photoexcited species and a second molecule of the complex, which is rather unlikely at sample concentrations of 3 and 6 mM.

By considering the full set of experimental IR spectra and the calculations, including the predicted reaction enthalpies, it can be concluded that [LM(CO)_3_(X_ax_)] is probably the main product of the photochemical reaction upon irradiation at 355 nm, independent of the solvent (MeCN and py) and the metal center (Cr, Mo, W). The conceivable primary reaction step with loss of a CO ligand was further analyzed by IR spectroscopy in the solid state (KBr matrix and frozen solution) at low temperature (see below). This assignment with photofragmentation of one M−CO bond and formation of the respective photoproduct with an axially coordinated solvent molecule is in accordance with literature reports on related systems such as [Mo(CO)_4_(iPr‐DAB)] (DAB: 1,4‐diaza‐butadiene) and [Cr(CO)_4_(bpy)] (bpy: 2,2’‐bipyridine).[^14^, ^18^]

A powerful tool to further investigate photo‐induced reactivity is represented by UV/VIS spectroscopy and consideration of absorption spectra of all three complexes in py after irradiation at 355 nm. Pyridine was the solvent of choice due to the higher stability of the formed photoproducts compared to MeCN (see below and ref. [19]). For the three complexes two low‐energy absorption bands are localized at 499–465 and 377–398 nm in py (Figure S64), which had been assigned to MLCT transitions earlier.^[20]^ The spectral blue‐shift from **Cr** via **W** to **Mo** for the lowest‐energy absorption band is in accordance with the absorption in other organic solvents.^[20]^ Interestingly, the UV/VIS spectrum clearly changes upon intense laser irradiation with a significant blue‐shift of the strong low‐energy visible absorption band to 449–470 nm (Figure S65). The mentioned absorption band is very well described by the theoretical UV/VIS spectra of [LM(CO)_3_(py_ax_)], as calculated by time‐dependent DFT (Figure S66). The strong visible absorption band of the photoproduct is a charge transfer transition from the metal center and the CO ligands to the coordinated pyridine ring and the bidentate pyridyl‐MIC ligand (Tables S2–S4). Thus, UV/VIS spectroscopy further strengthens the suggestions that are based on IR spectroscopy.

The eventual influence of the excitation wavelength on the formed photoproducts was investigated by irradiation of fresh solutions of the complexes at 532 and 266 nm, complementary to the investigations at 355 nm excitation presented above. The investigations at 266 nm had to be restricted to MeCN solutions, as pyridine shows an intense absorption at 266 nm. Interestingly, the IR spectra after irradiation at 532 and 266 nm show the same product bands as at 355 nm irradiation (Figures S6, S7 and S15), indicating that the photon energy has no significant influence on the formed photoproducts. Particular attention should be paid to the reactivity at 532 nm upon visible excitation into the lowest energy absorption band (see Figure S64 for the absorption in py and ref. [20] for MeCN). These observations clearly showed that at least one of the low‐lying electronically excited states is photochemically reactive. It should also be mentioned that the lower photon energy at 532 nm (225 kJ mol^−1^) is still far above the calculated reaction enthalpies of most potential photoproducts.

As irradiation at 532, 355 and 266 nm did not reveal any significant changes with respect to the formed main photoproduct, potential differences in the efficiency of reaction were considered by determination of the photodissociation quantum yields at these excitation wavelengths. The quantum yield of the photochemical reaction was determined from the fraction of reacted complex molecules relative to the number of absorbed photons, independent of the type of photoproduct which is formed. This evaluation was performed by considering the evolution of the IR spectrum during a short period of irradiation of few seconds at 532, 355 and 266 nm. The number of absorbed photons was determined from the respective UV/VIS extinction coefficients (Table S1). Irradiation times were kept short to minimize an undesired influence of the dark reverse reaction (see the next section) and follow‐up reactions induced by absorption of a second photon by the initially formed photoproducts. **Cr** revealed to be the most reactive complex with a photodissociation quantum yield (*ϕ*_diss_) of 43 % at 355 nm in MeCN with **Mo** and **W** showing lower quantum efficiencies of 15 and 5 %, respectively (Table 1). No significant influence was observed using pyridine as solvent instead of MeCN. The quantum yields obtained for **Cr**, **Mo**, and **W** in MeCN and pyridine are of the order of those reported for related M(CO)_4_(diimine) complexes in the literature.[^5^, ^6^, ^7^, ^8^, ^14^] The described trend of **Cr** ≫ **Mo** > **W** is known from the related α‐diimine carbonyl complexes of Cr^0^, Mo^0^ and W^0[6,7,14,30]^ and could result from efficient intersystem crossing towards low‐reactive ^3^MLCT states for **W** with its heavy tungsten center.^[14]^ Indeed, our previous photophysical studies on solid samples of **Cr**, **Mo**, and **W** yielded the highest phosphorescence quantum yields for tungsten.^[20]^ Interestingly, the quantum yields of the photochemical dissociation increase with decreasing irradiation wavelength resulting in a rise by more than one order of magnitude from 532 to 266 nm excitation (Table 1). This means on the one hand, that at least one low‐energy excited state is photochemically reactive and on the other hand, that also one or several higher energy excited states show photochemical reactivity. The increase of the quantum efficiency with decreasing excitation wavelength is known from related α‐diimine carbonyl complexes and is assigned to a higher photochemical reactivity of energetically higher MLCT states compared to the lower ones.[^5^, ^6^, ^8^, ^14^, ^18^] Beyond this, extensive studies on [Cr(CO)_4_(2,2’‐bipyridine)] revealed that the excitation wavelength has a significant influence on the ultrafast dynamics in the first hundreds of femtoseconds after excitation and herewith on the relative population of reactive and unreactive excited states. Excitation with high energy photons favors the population of reactive ^1^MLCT states.[^31^, ^32^]

**Table 1:** Photodissociation quantum yields of **Cr**, **Mo**, and **W** in MeCN (*λ*_ex_=532, 355, 266 nm) and py (*λ*_ex_=532, 355 nm).

		*ϕ*_diss_ [%}]		
	*λ*_ex_ [nm]	**Cr**	**Mo**	**W**
MeCN	532	0.9	0.6	0.3
	355	43	15	5
	266	46	44	10
py	532	0.9	0.5	0.3
	355	36	14	3.4

At this point, we wish to highlight the reversibility of the presented complexes to their initial species in a slow dark reaction. This reversibility is quite surprising for the investigated carbonyl complexes, as it has, to the best of our knowledge, only been reported for the homoleptic hexacarbonyl complex W(CO)_6_ so far.[^9^, ^11^] Preliminary observations concerning the reverse reactions for the systems **Cr** and **Mo** have been presented in an earlier publication,^[19]^ but profound kinetic studies were up to now hampered by limited reproducibility. Herein, we report on the kinetics of the dark reverse reaction, which were analyzed under optimized conditions with a homogenous irradiation of the sample volume by two laser beams.

**Figure 7:**
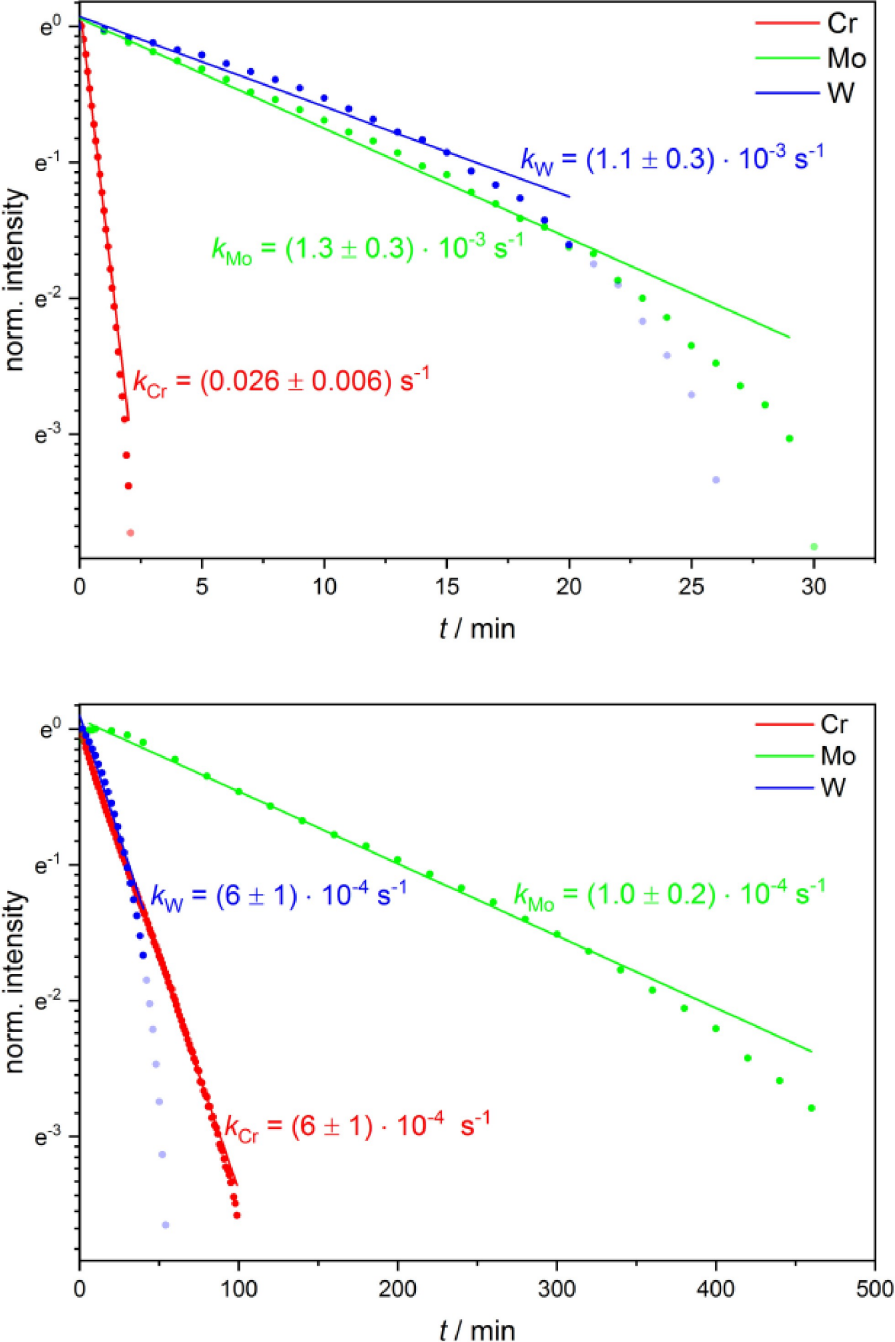
First‐order kinetic plots and fits of the dark reverse reaction of **Cr**, **Mo**, and **W** measured in MeCN (top) and py (bottom; *c*=6 mM). The shown kinetics were recorded after irradiation at 532 nm. The IR intensity at the end of the irradiation period (*t*=0) was normalized to 1.

Concretely, the kinetics of the dark reverse reaction were analyzed in MeCN and pyridine by considering the decrease of the strong new band at 1778–1784 cm^−1^, which is clearly assigned to the main photoproduct and is well separated from the other absorption features. The dark reaction was monitored subsequent to a period of irradiation at 355 nm (15 or 20 s for **Cr**, 60 s for **Mo** and **W**) or 532 nm (15 s for **Cr**, 60 or 120 s for **Mo** and **W**) with conversion of significant amounts of the initial complex to the photoproducts (Figures 3, S1–S6 and S9–S15). Hereby, the experimental IR intensity of the considered photoproduct band at the end of the irradiation period was normalized to 1 as the photoproduct concentration could not be accurately determined with the available techniques. Experiments were performed at concentrations of 6 and 3 mM with the laser power being adapted accordingly. The decay of the photoproduct band can be described by a first‐order kinetic fit for all three complexes, yet with deviations at longer time scales for **Mo** and **W** (Figures 7 and S67–S69). At the same time, the second order model kinetic plots do not yield the linear slopes expected for a bimolecular reaction (Figures S70–S73). For the second order evaluation, it was assumed that the photoproduct concentration is throughout identical to that of solvated CO. At first sight, the much better agreement of the first‐order kinetics is surprising for this bimolecular reverse reaction. However, it may be explained by a rate limiting initial thermal loss of the coordinated solvent molecule, before the formed reactive intermediate with 16 valence electrons reacts with CO to the initial complex (**Cr**, **Mo**, **W**).

Interestingly, the obtained reaction constants in MeCN strongly depend on the metal center as the dark reverse reaction is about one order of magnitude faster for **Cr** (*k*_Cr_(MeCN)=0.019–0.026 s^−1^) compared to the lower values of **Mo** and **W** (*k*_Mo_(MeCN)=(1.3–1.9)×10^−3^ s^−1^; *k*_W_(MeCN)=(1.1–2.4)×10^−3^ s^−1^ (Table 2).

**Table 2:** First‐order reaction constants of the dark reverse reaction for **Cr**, **Mo**, and **W** in MeCN and py after 532 or 355 nm irradiation.

	*c* [mM]	*λ*_ex_ [nm]	*K* [s^‐1^]		
			Cr	Mo	W
MeCN	6	532	0.026± 0.006	(1.3± 0.3)×10^−3^	(1.1± 0.3)×10^−3^
	3	532	0.019± 0.005	(1.4± 0.4)×10^−3^	(2.4± 0.6)×10^−3^
	6	355	0.023± 0.006	(1.9± 0.5)×10^−3^	(1.7± 0.4)×10^−3^
	3	355	0.025± 0.006	(1.7± 0.4)×10^−3^	(1.4± 0.4)×10^−3^
py	6	532	(6± 1) 10^−4^	(1.0± 0.2)×10^−4^	(6± 1)×10^−4^
	3	532	(5± 1) 10^−4^	(2.0± 0.5)×10^−4^	(1.2± 0.3)×10^−4^
	6	355	(8± 2) 10^−4^	(1.7±0.4)×10^−4^*	(1.5± 0.4)×10^−4^
	3	355	(6.1± 0.2) 10^−4^	(4± 1)×10^−4^	(8± 2)×10^−4^

* It should be noted that the underlying fit shows larger deviations from the experimental data.

In pyridine no clear trend was observed with respect to the correlation between the kinetics and the metal. **Cr** only gave clearly the highest kinetic rate at an irradiation wavelength of 355 nm and a concentration of 6 mM. At 532 nm irradiation in pyridine the reaction constants of **W** surpass those of **Cr**. Furthermore, the obtained results for **Mo** and **W** in pyridine strongly depend on the wavelength of the preceding irradiation (355 vs. 532 nm) and on concentration (6 vs. 3 mM). These observations for **Mo** and **W** in pyridine suggest at least two competing pathways for the reverse reaction under these conditions and are not yet fully understood. The impact of the solvent is more pronounced and obvious with reverse reactions that are at least one order of magnitude slower in pyridine compared to MeCN (Table 2, Figures 7 and S67–S69). In pyridine the photoproduct of **Mo** is clearly detectable several hours after the irradiation period and is thus particularly stable. This is in accordance with the observations reported in an earlier publication.^[19]^ The higher stability of the photoproducts containing pyridine relative to those with MeCN can be explained by the strong π ‐backbonding in case of the py ligand. Selected absolute and difference spectra of the kinetic experiments are represented in Figures 3, S1–S5 and S9–S14.

It is concluded that the general trend of **Cr** ≫ **Mo**≈**W** found for the dark kinetics in MeCN is similar to the results for the photodissociation quantum yields (**Cr** ≫ **Mo**>**W**). The initial photochemical reaction and the subsequent dark reaction seem to be more favorable for **Cr**. The initial step is best described as cleavage of a coordinative M−CO and M–solvent (acetonitrile/pyridine) bond, respectively (see low‐temperature IR spectroscopy). Such a dissociative mechanism has also been reported for related Cr complexes such as [Cr(CO)_4_phen] (phen=phenanthroline) by van Eldik et al., but the analogous Mo and W complexes rather follow associative substitution pathways.[^8^, ^33^]

**Figure 8:**
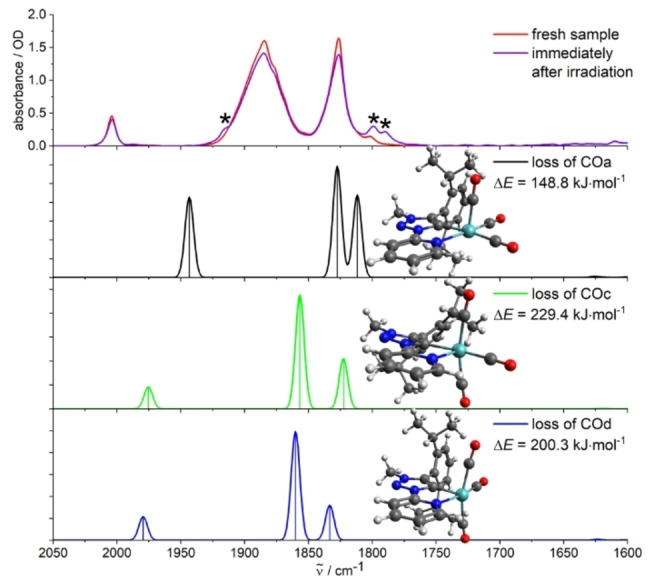
Irradiation (*λ*_ex_=355 nm) of fresh samples of **Mo** (KBr pellet) at 10 K and calculated IR spectra of different photoproducts with loss of a CO ligand, including the optimized structures and calculated enthalpies of reaction. The vacant coordination site is localized in the axial position for isomer a and in the equatorial position for structures c/d. Calculations: DFT/B3LYP−D3(BJ)/def2‐TZVP/COSMO, scaling factor: 0.99, Gaussian convolution with FWHM=8 cm^−1^.

Temperature‐dependent photochemical studies on **Cr**, **Mo**, and **W** were performed in a KBr matrix to obtain more insights with respect to the reaction pathway and long‐lived intermediates. Irradiation at 355 nm of KBr pellets cooled down to 10 K yielded weak new absorption bands/shoulders in the regions of 1918–1905 cm^−1^ (one signal) and 1813–1763 cm^−1^ (two signals), with small spectral shifts depending on the metal center (Figures 8 and S60–S61). Furthermore, small signals appeared at 2123–2183 cm^−1^ in the respective difference spectra, which are assigned to traces of unbound or weakly bound CO in the matrix upon irradiation (Figure S18). Thus, reactivity was obtained in the solid state without the presence of any obvious reaction partner. The traces of free CO indicate the cleavage of M−CO bonds, similar to the observations in liquid solution. Considering the experimental spectra, theoretical calculations were performed for three potential photoproducts in the solid state with loss of a single CO ligand and a vacant coordination site ([LM(CO)_3_]) (Figures 8, S60, and S61). The theoretical spectra of the optimized structures with loss of an axial CO ligand are indeed in very good agreement with the experiment for all three complexes. The isomers with a vacant equatorial coordination site describe the recorded IR spectra only to a smaller extent and are higher in energy.

These statements are in perfect agreement with the conclusions deduced from the photochemical studies in solution, where [LM(CO)_3_(X_ax_)] was best described as the main photoproduct.

In the KBr matrix with absence of a suitable reaction partner, it is conceivable that the assigned species [LM(CO)_3_] with 16 valence electrons is stabilized at 10 K. The weak product features must result from a very limited conversion of the initial species to the photoproducts in the solid sample. A specific orientation and packing on a molecular level may be required for photo‐induced reactivity. The formed photoproduct is stabilized to full extent at 10 K over at least 30 min in the dark. However, the intensity of the product features decreases steadily upon heating stepwise to 290 K after irradiation at 10 K, accompanied by an increase of the absorption bands of the initial complex. Hence, the reaction in the solid state is reversible at least at sufficient thermal energy (Figures S19–S21). This means that a certain amount of photoproduct is efficiently stabilized over time at a specific temperature and, accordingly, irradiation of a fresh sample at a chosen temperature yields a certain amount of stable photoproduct (Figures S22–S24). No reaction was observed in the static spectra at 290 K, which most probably results from a very fast reverse reaction, impeding the accumulation of detectable amounts of photoproduct. The fast reverse reaction at higher temperature may be a result of stronger fluctuations within the KBr matrix at higher thermal energy, making the trapping of uncoordinated CO less efficient and leading to its recombination with the very reactive 16 valence electron species. [LM(CO)_3_] is far too short‐lived to be detectable with the applied techniques in solution at room temperature. In this context, it should be noticed that the transient femtosecond pump–probe experiments required for the observation of such short‐lived intermediates would most likely be hampered by the rather low photochemical reactivity of the systems (in particular **Mo** and **W**). This drawback had led to very weak step‐scan FTIR signals in preliminary works.^[19]^ In accordance with the reported photodissociation quantum yields in solution (MeCN and py), the photochemical reaction in the solid state is, compared to excitation at 355 nm, less efficient at 532 nm and more pronounced at 266 nm according to the relative spectral intensities (Figures S25–S27). This photochemistry in the solid state is closely related to the reported reactivity of the hexacarbonyls of chromium, molybdenum and tungsten in a cold matrix (KBr,^[9]^ PMMA (poly(methyl methacrylate))^[15]^ and inert‐gas matrix^[10]^ with a loss of a CO ligand and formation of the reactive species M(CO)_5_ (M=Cr^0^, Mo^0^ or W^0^).

**Figure 9:**
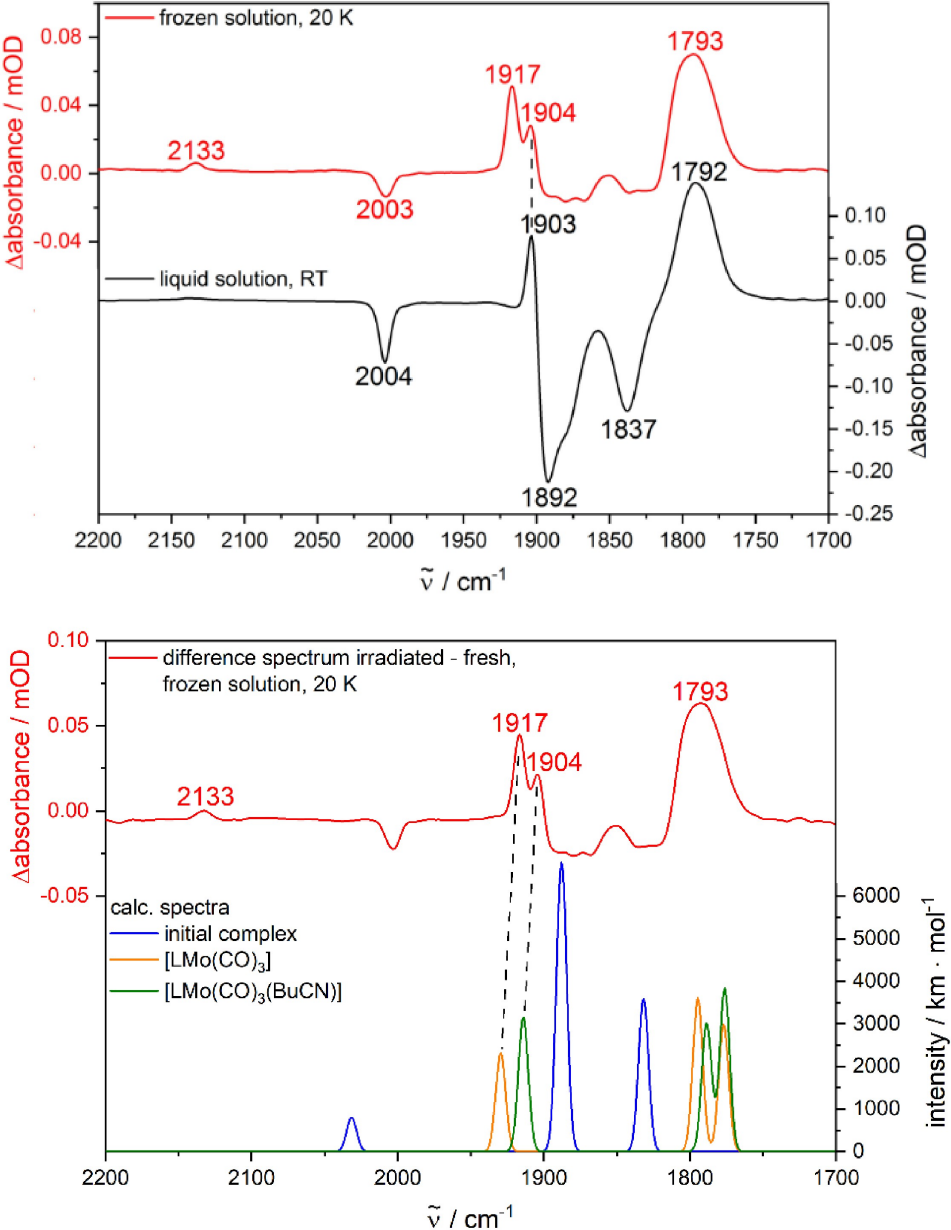
Top: Difference spectra (irradiated−fresh) of **Mo** recorded in frozen (20 K) and liquid (RT) solutions. Bottom: Comparison of the difference spectrum at 20 K with calculated IR spectra of the initial complex as well as the photoproducts [LMo(CO)_3_] (vacant coordination site in axial position) and [LMo(CO)_3_BuCN_ax_]. The dashed lines are guides to the eye. Calculations: DFT/B3LYP−D3(BJ)/def2‐TZVP/COSMO, scaling factor: 0.99, Gaussian convolution with FWHM=8 cm^−1^.

Complementary studies were performed in frozen valeronitrile (BuCN) at 10 K in order to confirm the formation of the reactive species [LM(CO)_3_] also under conditions that are closer to the studies in liquid solution. BuCN was selected not only for its ability to form a clear glass at the melting point, but also for its ligand properties comparable to MeCN. As expected, irradiation (*λ*_ex_=355 nm) of fresh solutions of **Cr**, **Mo**, and **W** in BuCN at room temperature yielded IR spectra with product bands very similar to those in MeCN and pyridine (Figure S17). Concerning the experiments in frozen solution, the measurement of absolute spectra turned out to be difficult due to varying scattering effects induced by the glass matrix when comparing the sample scans with the reference consisting of pure valeronitrile. Hence, the following discussions are limited to difference spectra representing the spectral changes induced by irradiation at 355 nm. The difference spectrum of **Mo** shows intense positive peaks at 1917 and 1904 cm^−1^ as well as a broad positive band at 1793 cm^−1^ (Figure 9). A weak positive band was observed at 2133 cm^−1^, which can be assigned to free CO under consideration of the studies in solution and KBr presented before. Surprisingly, the negative bands in the difference spectrum obtained in frozen BuCN are very weak compared to the positive ones, which might be explained by higher oscillator strengths of the photoproduct peaks in this medium. The comparison of the difference spectra of **Mo** obtained in liquid and frozen BuCN revealed that the peaks at 1903 cm^−1^ in liquid solution and at 1904 cm^−1^ in frozen solution result from the same product species (Figure 9). The positive peak at 1792 cm^−1^ is broader in frozen glass compared to the liquid phase and could indicate the superposition of the absorption features of at least two different structures in this spectral region for the frozen matrix. Furthermore, an additional band was observed at 1917 cm^−1^ in the glass, which was completely absent at room temperature. Under consideration of this experimental result, the new peak at 1917 cm^−1^ was preliminarily attributed to a reactive intermediate, similar to the observations in a KBr matrix at low temperature. For clarification, theoretical calculations were performed in BuCN. Indeed, the calculated IR spectra of [LM(CO)_3_] and [LM(CO)_3_BuCN_ax_] suggest their coexistence in frozen solution. Concerning the experimental double band around 1910 cm^−1^, theory predicts a slight red‐shift for the structure with a bound BuCN molecule, in agreement with the experimental difference spectra in frozen and liquid BuCN (Figure 9). The same descriptions are valid for **Cr** and **W** (Figures S62 and S63), both from an experimental and theoretical point of view. Finally, the photochemical studies in frozen solution clearly show that the mechanistic pathway in organic solvents involves the presented species [LM(CO)_3_] as metastable intermediate, followed by formation of [LM(CO)_3_(X_ax_)] with a solvent molecule in axial position.

The first step in the reaction mechanism is preferably a cleavage of an axial M−CO bond as these are elongated compared to those of the equatorial CO ligands according to theory (Table S5) and the crystal structures reported earlier.[^19^, ^20^] In this case, the mechanism could take place without ligand‐sphere reorganization with respect to the orientation of the CO ligands. This suggestion is in full agreement with ultrafast spectroscopy on [Cr(CO)_4_(2,2’‐bipyridine)], which did not show any structural reorganization after the initial photochemical loss of CO.^[32]^

## Conclusion

**Figure 10:**
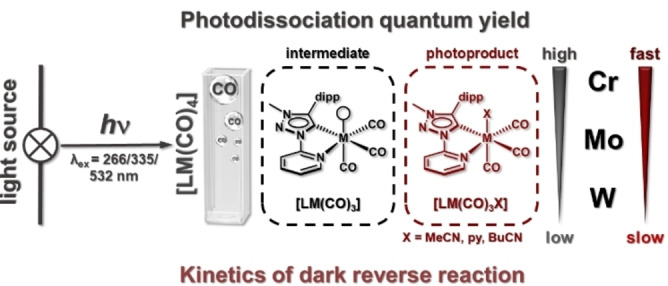
Graphical summary of the (photo)chemistry of **Cr**, **Mo**, and **W** in organic solution (MeCN, py, BuCN). The given molecular structures of intermediate and photoproduct are those of the species mainly observed. The illustrated trends for the photodissociation quantum yield and the kinetics of the dark reverse reaction are fully valid for MeCN, with a few exceptions for py.

In conclusion, profound spectroscopic and theoretical studies were performed on the photochemical properties of a series of pseudo‐octahedral Cr^0^, Mo^0^ and W^0^ carbonyl complexes containing a bidentate pyridyl‐mesoionic carbene ligand (Figure 10). According to IR spectroscopy in combination with density functional theory (DFT), in common coordinating organic solvents (acetonitrile, valeronitrile and pyridine), UV and visible irradiation induce a clean photochemistry with monosubstitution of an axial CO ligand for a solvent molecule. FTIR spectroscopy at low temperatures revealed a metastable reactive intermediate with a vacant axial coordination site, thereby pointing to a reaction without ligand–sphere reorganization upon photo‐induced cleavage of one of the two weakest coordinative bonds (CO_axial_). The photodissociation quantum yield increases from W^0^ over Mo^0^ to Cr^0^ (values of up to 46 %) independently of the solvent. The values in acetonitrile are higher throughout compared to those in pyridine and increase with decreasing irradiation wavelength. The most remarkable observation for the investigated complexes is definitely the reversibility of the photochemical reaction with recovery of at least the majority of the starting complex in a dark reverse reaction consecutive to irradiation. The reverse reaction is faster for Cr^0^ (minutes) than for Mo^0^ and W^0^ in MeCN and can take several hours for Mo^0^ and W^0^ in pyridine. This atypical reversibility will be investigated by further theoretical studies in the future with a focus on mechanistic pathways and, in particular, energy barriers. The photochemistry, with one preferred reaction pathway, would be extremely useful for applications in highly selective photochemical synthesis, and its reversibility opens a new field of (photo)switchable transition metal complexes.

## Experimental Section

Experimental details are provided in the Supporting Information.

## Conflict of interest

The authors declare no conflict of interest.

1

## Supporting information

As a service to our authors and readers, this journal provides supporting information supplied by the authors. Such materials are peer reviewed and may be re‐organized for online delivery, but are not copy‐edited or typeset. Technical support issues arising from supporting information (other than missing files) should be addressed to the authors.

Supporting Information

## Data Availability

The data that support the findings of this study are available in the supplementary material of this article.
