# Supplementary material for: Mechanistic and Kinetic Investigations of ON/OFF (Photo)Switchable Binding of Carbon Monoxide by Chromium(0), Molybdenum(0) and Tungsten(0) Carbonyl Complexes with a Pyridyl‐Mesoionic Carbene Ligand
Source: Chemistry. 2022 Jul 13;28(51):e202201038. doi: 10.1002/chem.202201038 (PMC9542575; doi:10.1002/chem.202201038)
Supplement: Supplementary file 1 — Supporting Information [file CHEM-28-0-s001.pdf]

Chemistry–A European Journal

Supporting Information

Mechanistic and Kinetic Investigations of ON/OFF (Photo)Switchable Binding of Carbon Monoxide by Chromium(0), Molybdenum(0) and Tungsten(0) Carbonyl Complexes with a Pyridyl-Mesoionic Carbene Ligand

Pit J. Boden, Patrick Di Martino-Fumo, Tobias Bens, Sophie T. Steiger, Daniel Marhöfer, Gereon Niedner-Schatteburg,* and Biprajit Sarkar*

Table of Contents

1 Experimental Details 3

1.1 Static and time-resolved FTIR spectroscopy 3

1.1.1 General setup for time-resolved FTIR experiments 3

1.1.2 Sample preparations for FTIR spectroscopy in solution at room temperature 3

1.1.3 Kinetic studies by time-resolved FTIR spectroscopy 3

1.1.4 Determination of photodissociation quantum yields by FTIR spectroscopy 4

1.1.5 FTIR spectroscopy in frozen solution 4

1.1.6 FTIR spectroscopy in the solid state 4

1.2 UV/VIS absorption spectroscopy 5

1.3 Theoretical calculations 5

2 Experimental IR spectra 6

2.1 Acetonitrile 6

2.2 Pyridine 14

2.3 Valeronitrile 21

2.4 KBr matrix 22

3 Detection of CO with the detection reagent $PdCl_2(MeCN)_2$ 30

4 Comparison between experimental and theoretical IR spectra 31

4.1 IR spectra of Cr, Mo and W 31

4.2 Monosubstitution 32

4.2.1 Monosubstitution in acetonitrile 32

4.2.2 Monosubstitution in pyridine 34

4.3 Bisubstitution 37

4.3.1 Bisubstitution in acetonitrile 37

4.3.2 Bisubstitution in pyridine 40

4.4 Trisubstitution 43

4.4.1 Trisubstitution in acetonitrile 43

4.4.2 Trisubstitution in pyridine 46

4.5 Formation of a metallaketene ... 49
4.5.1 Formation of a metallaketene in acetonitrile ... 49
4.5.2 Formation of a metallaketene in pyridine ... 52
4.6 Further isomerizations ... 55
4.6.1 Further isomerizations in acetonitrile ... 55
4.6.2 Further isomerizations in pyridine ... 58
4.7 Loss of a CO ligand in a KBr matrix ... 61
4.8 Photochemistry in frozen valeronitrile ... 63
5 Comparison between experimental and theoretical UV/VIS spectra ... 65
6 Kinetic data for the dark reverse reaction ... 69
7 Calculated bond lengths ... 76
8 References ... 77

1 Experimental Details

1.1 Static and time-resolved FTIR spectroscopy

1.1.1 General setup for time-resolved FTIR experiments

IR spectroscopy was performed with the FTIR spectrometer Bruker Vertex 80v. A liquid-nitrogen-cooled mercury cadmium telluride (MCT) detector (Kolmar Tech., Model KV100-1-B-7/190) with a rise time of 25 ns, connected to a fast preamplifier, was used for signal detection and processing. The laser setup includes a Q-switched Nd:YAG laser (Innolas SpitLight Evo I) generating pulses with a band-width of 6 – 8 ns at a repetition rate of 100 Hz. Either the second, third or fourth harmonic of the Nd:YAG laser (532, 355 and 266 nm, respectively) was used directly for sample excitation. The homogeneity of the irradiation of the whole sample volume was optimized by dividing the incoming beam of the Nd:YAG laser (8.0 mJ/shot at 532 nm, 4.0 – 5.5 mJ/shot at 355 nm, 4.0 – 5.0 mJ/shot at 266 nm if not stated otherwise) into two partial beams, which irradiated the sample from opposite directions.

The sample chamber was purged with argon and was equipped with anti-reflection-coated germanium filters to prevent the entrance of laser radiation into the detector and interferometer compartments. The spectral region was limited by undersampling either to 0 – 3949.5 cm^{-1} or 0 – 2633.5 cm^{-1} with a spectral resolution of 4 cm^{-1} resulting in 2221 and 1481 interferogram points, respectively. The germanium windows also prevented problems when performing a Fourier transformation as IR radiation outside the measured spectral window of 0 – 3949.5 cm^{-1} was efficiently absorbed, which was further guaranteed by the CaF_2 windows of the sample cell (no transmission < 1000 cm^{-1}). In the case of measurements covering the spectral range of 0 – 2633.5 cm^{-1}, the spectral region was limited by use of an IR longpass filter (no transmission > 2400 cm^{-1}).

1.1.2 Sample preparations for FTIR spectroscopy in solution at room temperature

Experiments in solution at room temperature were performed in a sealed optical cell (Specac Omni-Cell®) with two CaF_2 windows separated by 200 µm. Solutions of the complexes for IR spectroscopy were prepared at concentrations of 3 or 6 mM, depending on the experiment. Acetonitrile was purchased from Merck (Uvasol Grade) and pyridine from Acros Organics (anhydrous, ≥ 99.5 %). Solutions were handled under air if not stated otherwise. Investigations with $PdCl_2(MeCN)_2$ were performed with saturated solutions of the reagent, which was purchased from Sigma-Aldrich. Saturation of acetonitrile and pyridine solutions with carbon monoxide (CO) was performed by purging the solutions with argon and subsequently with CO for at least 20 min, respectively.

1.1.3 Kinetic studies by time-resolved FTIR spectroscopy

For the kinetic studies on the dark reverse reactions following an initial period of irradiation of 15 – 60 s (depending on the experiment) at 532 or 355 nm, FTIR spectra were recorded at regular intervals of 15 – 120 s in the dark subsequent to irradiation. Experiments were performed at concentrations of 6 and 3 mM with the laser power being adapted accordingly (355 nm: 4 mJ at 6 mM and 2 mJ at 3 mM; 532 nm: 8 mJ at 6 mM and 4 mJ at 3 mM). The decrease of the intensity of the strong new band at

1779 – 1784 cm^{-1} was considered over time, which is clearly assigned to the main photoproduct. The IR intensity of this photoproduct band at the end of the irradiation period (*t* = 0) was normalized to 1. The error bars for the kinetic rates are estimated to $\pm$ 25 %.

1.1.4 Determination of photodissociation quantum yields by FTIR spectroscopy

Photodissociation quantum yields were determined by constant irradiation of solutions (*c* = 3 or 6 mM) at 532, 355 or 266 nm and consideration of the photo-induced decrease of the isolated band of the initial complex at 1995 – 2006 cm^{-1}. The considered time region was limited to an irradiation period of $\leq$ 70 s at 532 nm and $\leq$ 5 s at 355 nm and 266 nm to avoid a large influence of the formed photoproduct(s) (in particular the photon absorption at 266 nm) and the much slower dark reverse reaction on the obtained quantum yields. Irradiation intensities (8.0 mJ/shot at 532 nm, 4.2 mJ at 355 nm/shot and 4.5 mJ/shot at 266 nm) were determined using a power meter and UV/VIS extinction coefficients were obtained *via* UV/VIS absorption spectroscopy. The higher laser power at 532 nm was selected to induce considerable conversion to the photoproducts for an accurate determination of the quantum yields despite the throughout poor efficiency at this wavelength. The time of irradiation, the irradiation power, the sample concentration, the cell volume (80 µL), the extinction coefficient and the IR intensity then yielded information on the number of reacted complex molecules per number of irradiated photons. The error bars for the photodissociation quantum yields are estimated to $\pm$ 25 %.

1.1.5 FTIR spectroscopy in frozen solution

Investigations on frozen solutions at 20 K were performed in valeronitrile ($\geq$ 99 %, purchased from Sigma-Aldrich) at a concentration of 6 mM. The samples were measured in a home-built cell consisting of two CaF_2 windows separated by 1 mm, which was sealed with high vacuum grease. The cell was cooled to 20 K with a closed-cycle helium cryostat (ARS Model DE-202A). The cryocooler was equipped with a cell holder and CaF_2 windows. Photochemical experiments were performed by irradiation over 8 min at 355 nm (3.0 mJ/shot).

1.1.6 FTIR spectroscopy in the solid state

KBr pellets were prepared by mixing neat powder of the compound (ca. 0.4 mg) with dry KBr (ca. 180 mg, stored in a compartment dryer at 80 °C, purchased from Merck) and grinding to a homogenous mixture. This mixture was filled in an evacuable pellet die with a diameter of 13 mm and sintered at a pressure of 0.75 GPa. The strongest peak in the ground state spectrum showed an absorption of $\geq$ 1 OD with the mentioned concentration. The measurements were performed at temperatures of 20 – 290 K using a closed-cycle helium cryostat (ARS Model DE-202A). The cryocooler was equipped with a pellet holder and CaF_2 windows. Photochemical investigations were performed by irradiation of the pellets over 8 min at 532, 355 or 266 nm (2.0 mJ/shot).

1.2 UV/VIS absorption spectroscopy

UV/VIS absorption experiments in solution were performed with a Lambda 900 UV/VIS/NIR spectrometer using 10 mm path length quartz cells at 25°C. The solutions were prepared at a concentration of $2 \cdot 10^{-5}$ M. The spectra were recorded relative to the pure solvent. The UV/VIS spectra of the photoproducts were recorded subsequent to a period of laser irradiation (3 min at 2.0 mJ/pulse at 100 Hz, 355 nm, Innolas SpitLight Evo I). The yielded solutions were centrifugated before the measurement of the UV/VIS spectrum of the photoproduct to prevent scattering losses due to insoluble photoproducts, which most probably resulted from side reactions.

1.3 Theoretical calculations

The crystal structures were used as input structures for the initial complexes and starting structures for conceivable photoproducts were generated by chemical intuition. Geometry optimizations were performed with the Berny algorithm of Gaussian 09[1] by using energies and gradients computed by Turbomole 7.4.[2,3] All calculations were performed with the DFT functional B3LYP with dispersion correction (no three-body interaction) (D3(BJ))[4] as implemented in Turbomole using the resolution of identity (RI) approximation and the def2-TZVP basis set.

Harmonic frequency calculations were performed for the optimized minimum structures. The influence of the medium (MeCN, valeronitrile, pyridine, KBr) was modulated by using the conductor-like screening model (COSMO). The vibrational frequencies are scaled by a factor of 0.99 to minimize the differences between the experimental and calculated frequencies. A Gaussian convolution with a full-width at half-maximum of 8 cm^{-1} was applied to the calculated vibrational transitions.

2 Experimental IR spectra

2.1 Acetonitrile

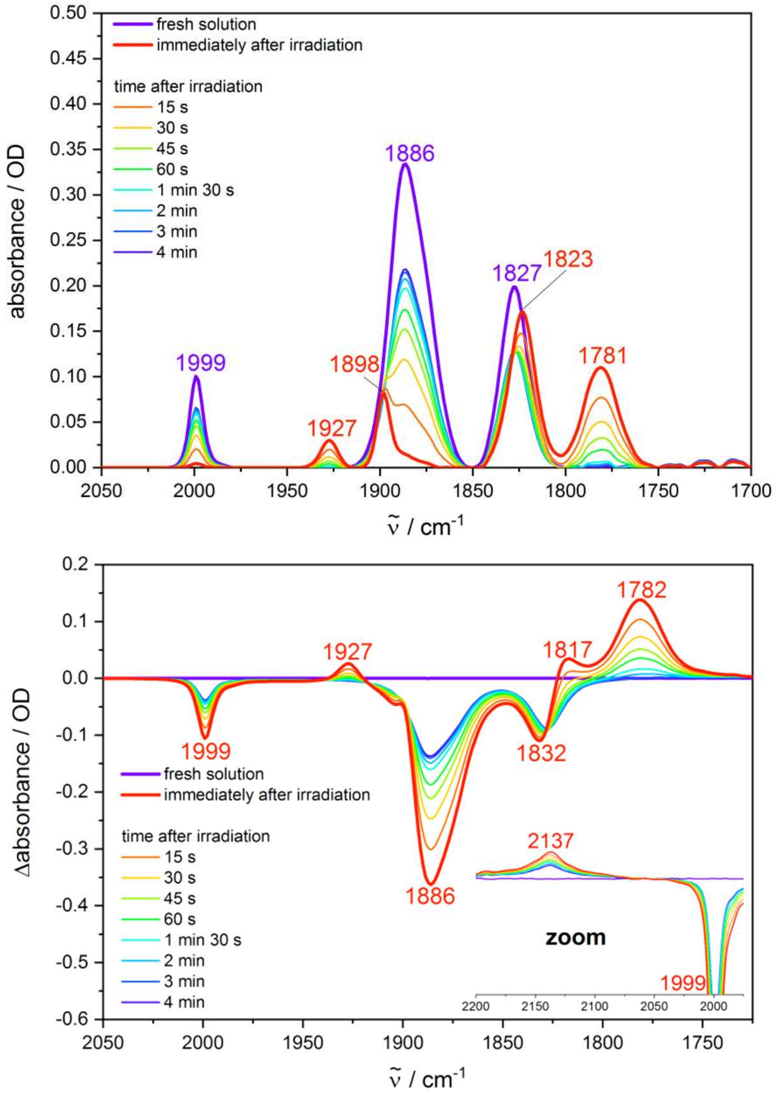

Figure S1. Irradiation (λ_{ex} = 355 nm) of a fresh solution of **Cr** in MeCN (c = 6 mM) and spectra recorded in the dark after irradiation (top: absolute absorption spectra, bottom: difference spectra).

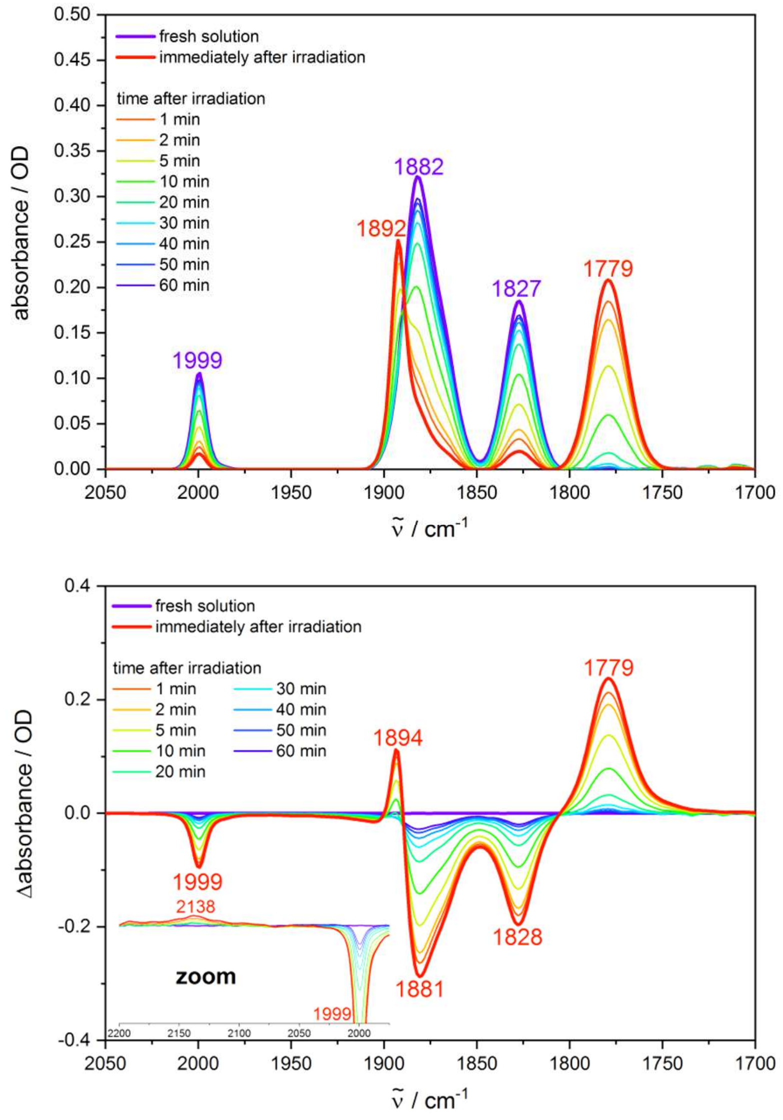

Figure S2. Irradiation (λ_{ex} = 355 nm) of a fresh solution of **W** in MeCN (c = 6 mM) and spectra recorded in the dark after irradiation (top: absolute absorption spectra, bottom: difference spectra).

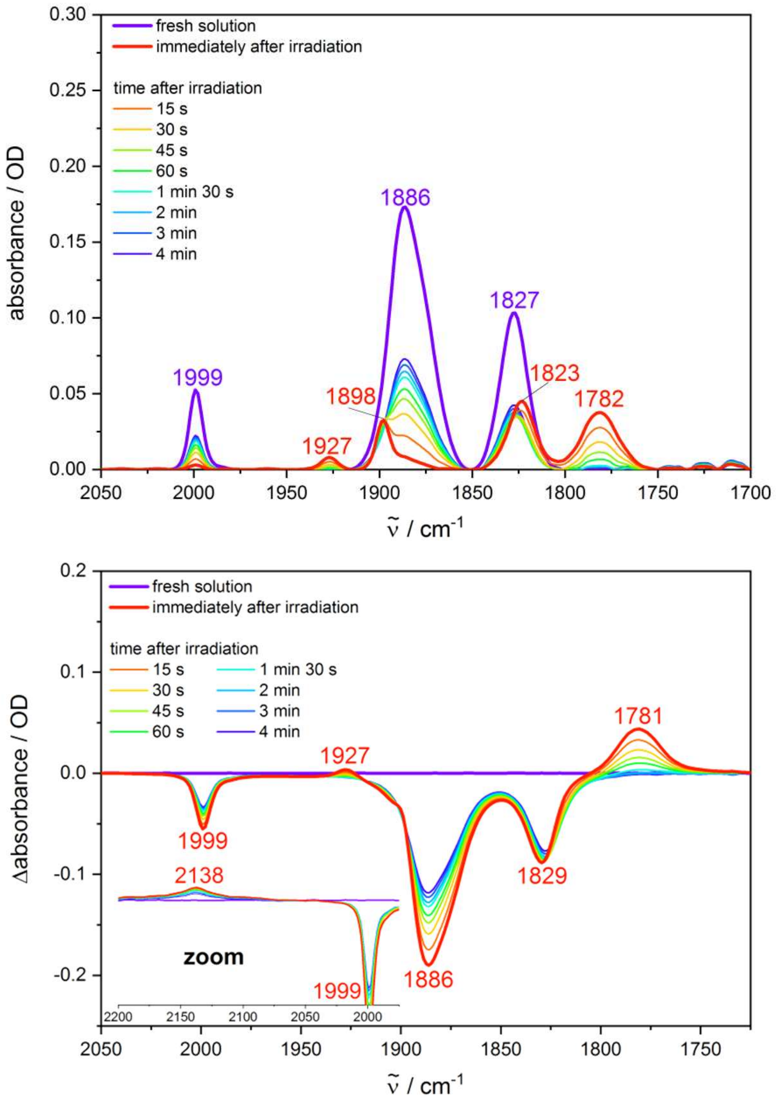

Figure S3. Irradiation (λ_{ex} = 355 nm) of a fresh solution of **Cr** in MeCN (c = 3 mM) and spectra recorded in the dark after irradiation (top: absolute absorption spectra, bottom: difference spectra).

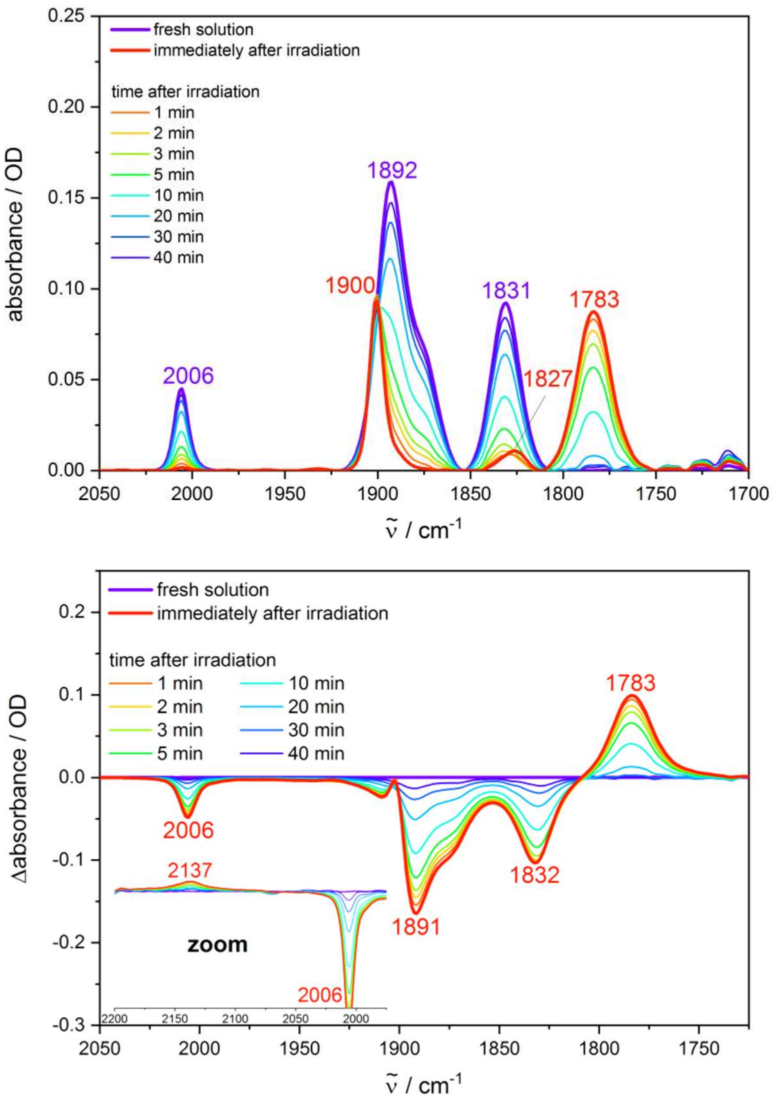

Figure S4. Irradiation (λ_{ex} = 355 nm) of a fresh solution of **Mo** in MeCN (c = 3 mM) and spectra recorded in the dark after irradiation (top: absolute absorption spectra, bottom: difference spectra).

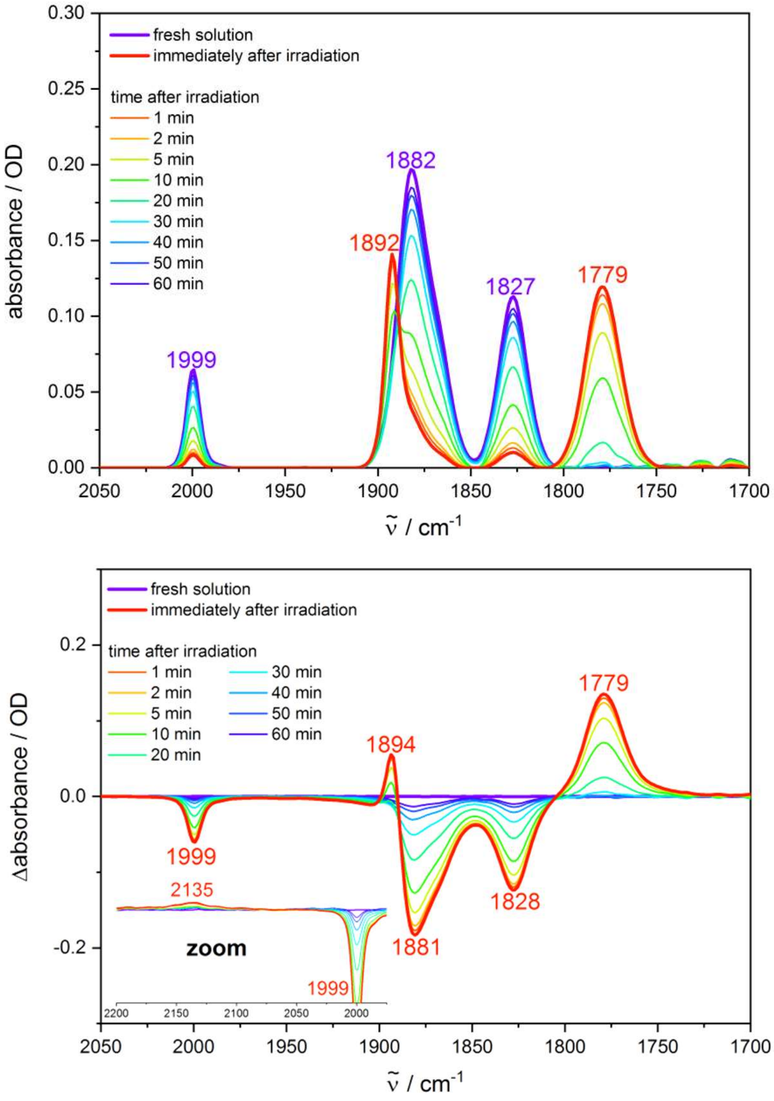

Figure S5. Irradiation (λ_{ex} = 355 nm) of a fresh solution of **W** in MeCN (c = 3 mM) and spectra recorded in the dark after irradiation (top: absolute absorption spectra, bottom: difference spectra).

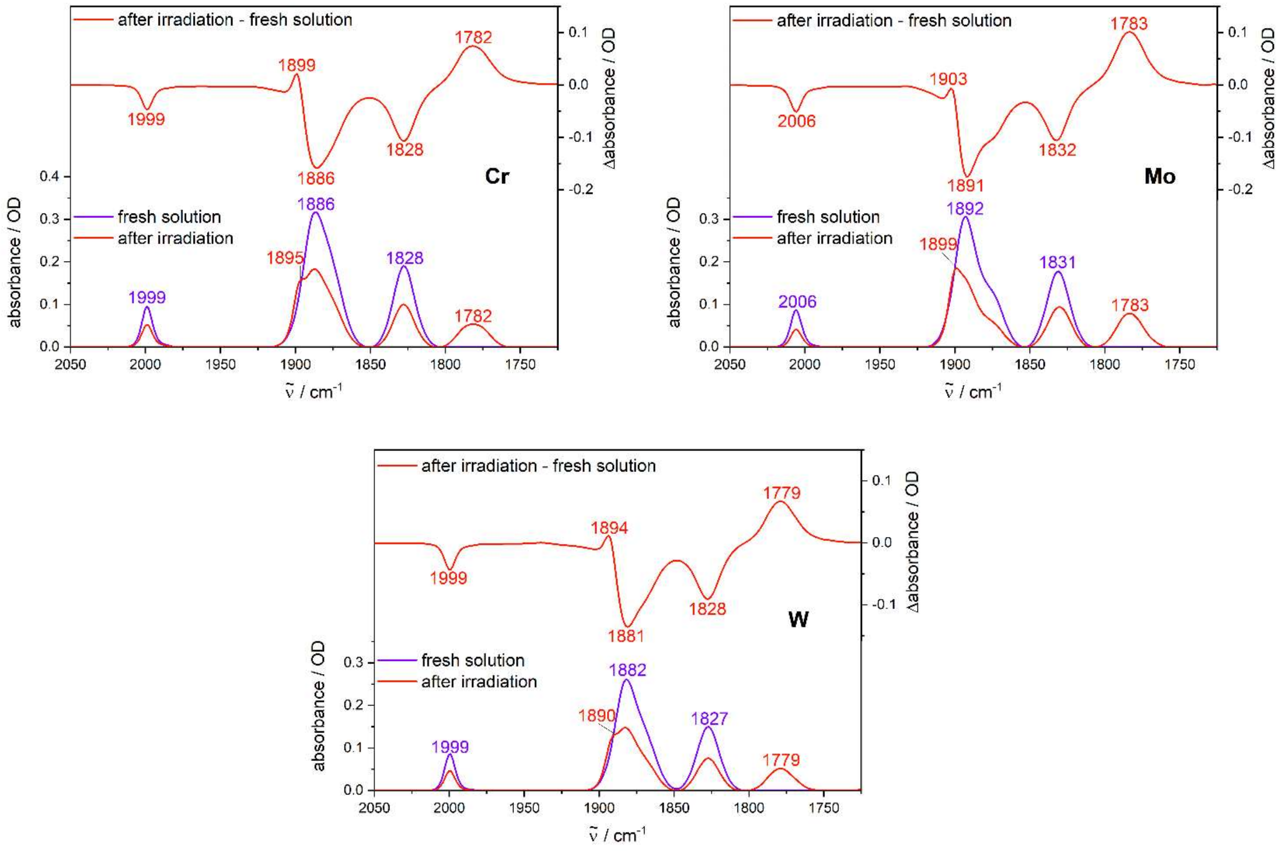

Figure S6. Irradiation (λ_{ex} = 532 nm) of fresh solutions of **Cr** (top, left), **Mo** (top, right) and **W** (bottom) in MeCN (c = 6 mM) with representation of the absolute spectra before and immediately after irradiation as well as the corresponding difference spectrum (irradiated – fresh).

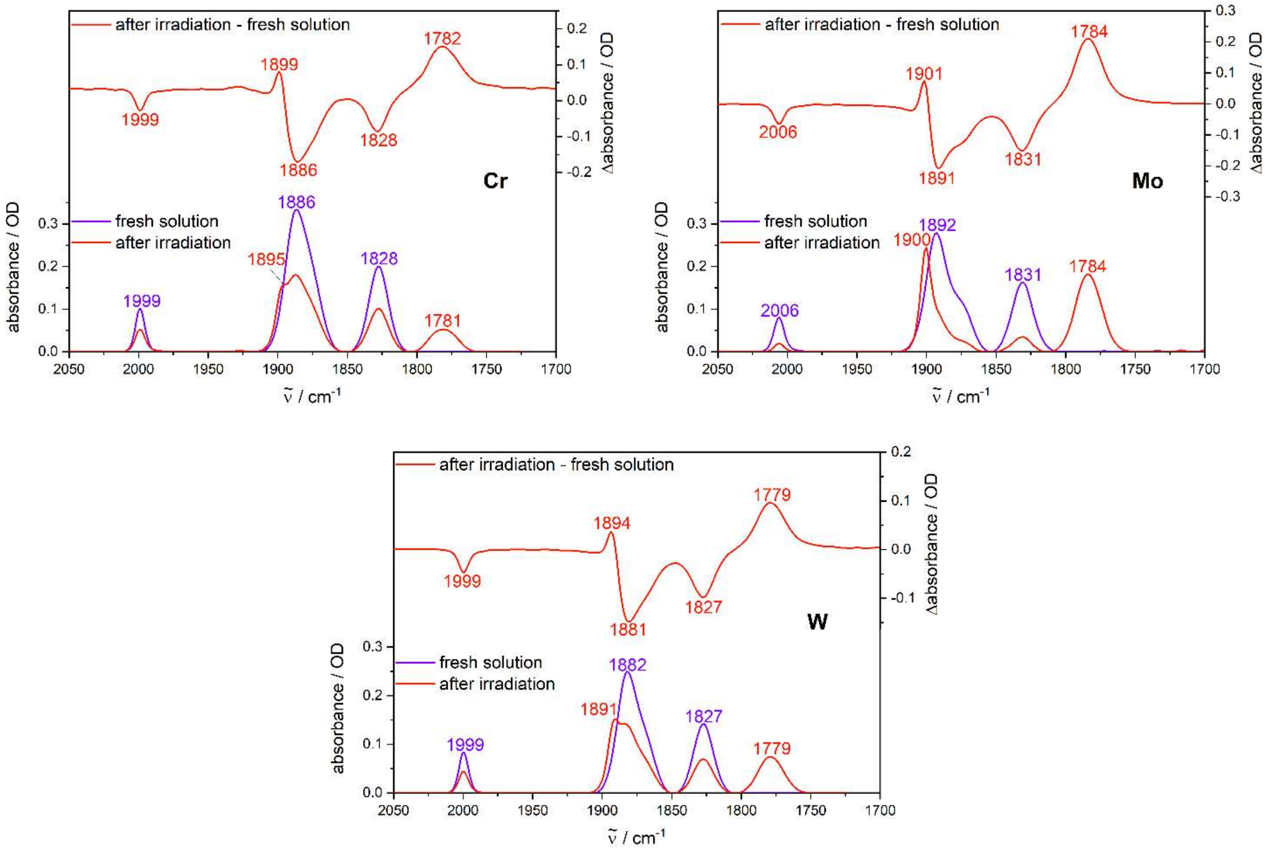

Figure S7. Irradiation (λ_{ex} = 266 nm) of fresh solutions of **Cr** (top, left), **Mo** (top, right) and **W** (bottom) in MeCN (*c* = 6 mM) with representation of the absolute spectra before and immediately after irradiation as well as the corresponding difference spectrum (irradiated – fresh).

Figure S8. IR spectrum of CO dissolved in MeCN.

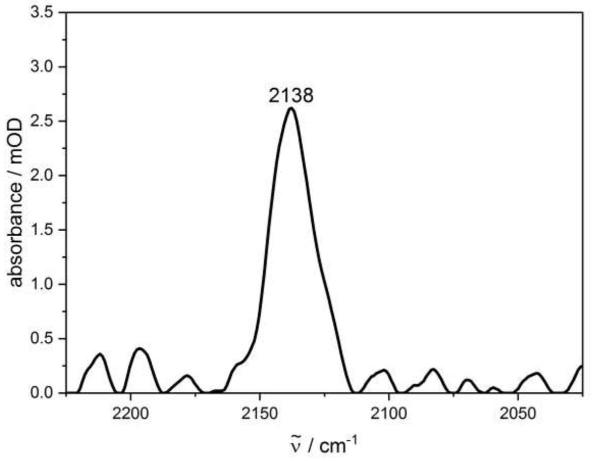

2.2 Pyridine

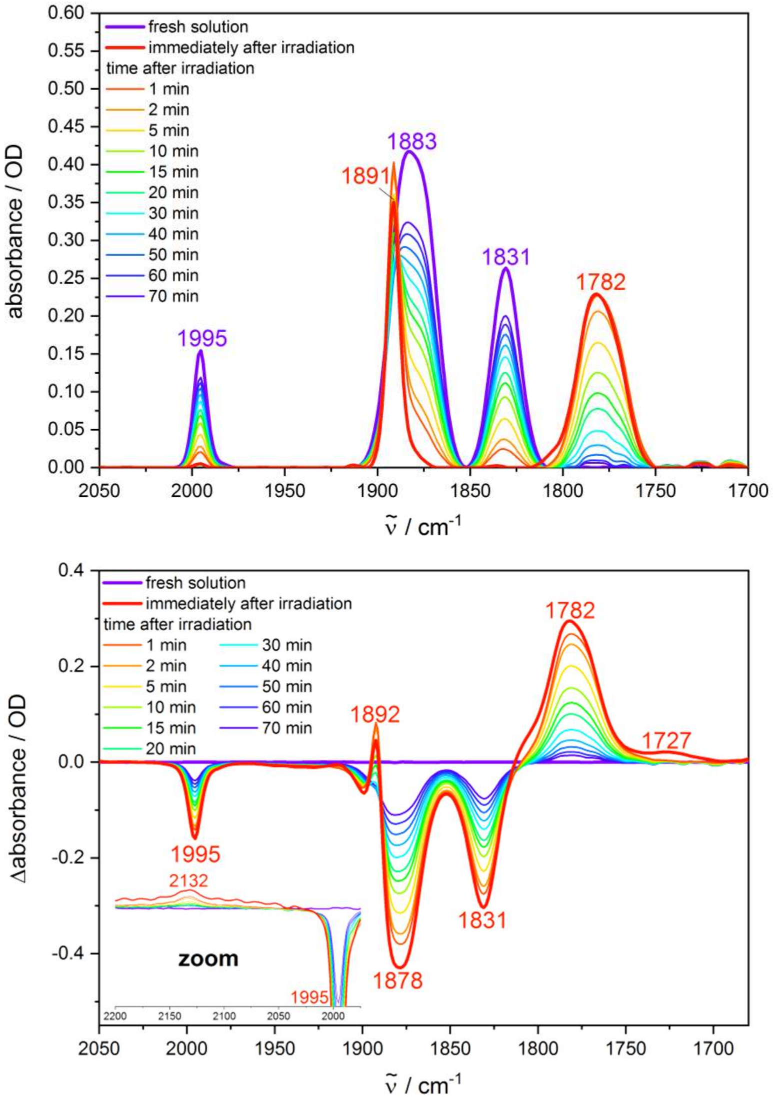

Figure S9. Irradiation (λ_{ex} = 355 nm) of a fresh solution of **Cr** in py (c = 6 mM) and spectra recorded in the dark after irradiation (top: absolute absorption spectra, bottom: difference spectra).

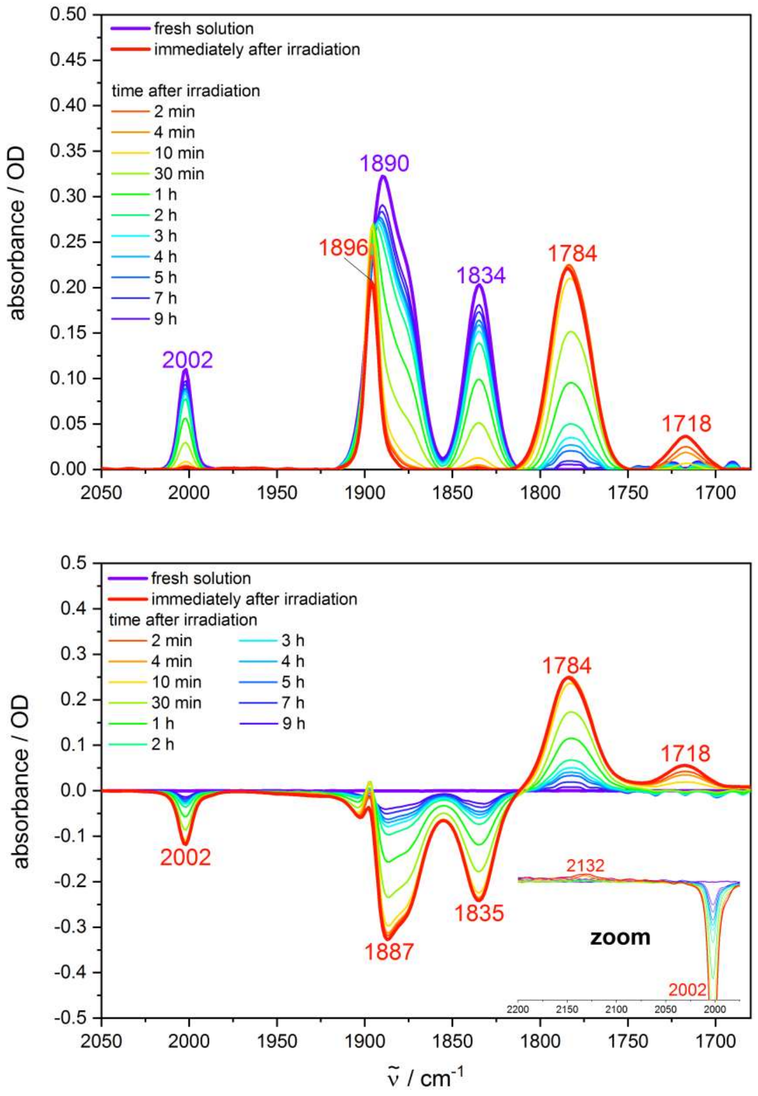

Figure S10. Irradiation (λ_{ex} = 355 nm) of a fresh solution of **Mo** in py (c = 6 mM) and spectra recorded in the dark after irradiation (top: absolute absorption spectra, bottom: difference spectra).

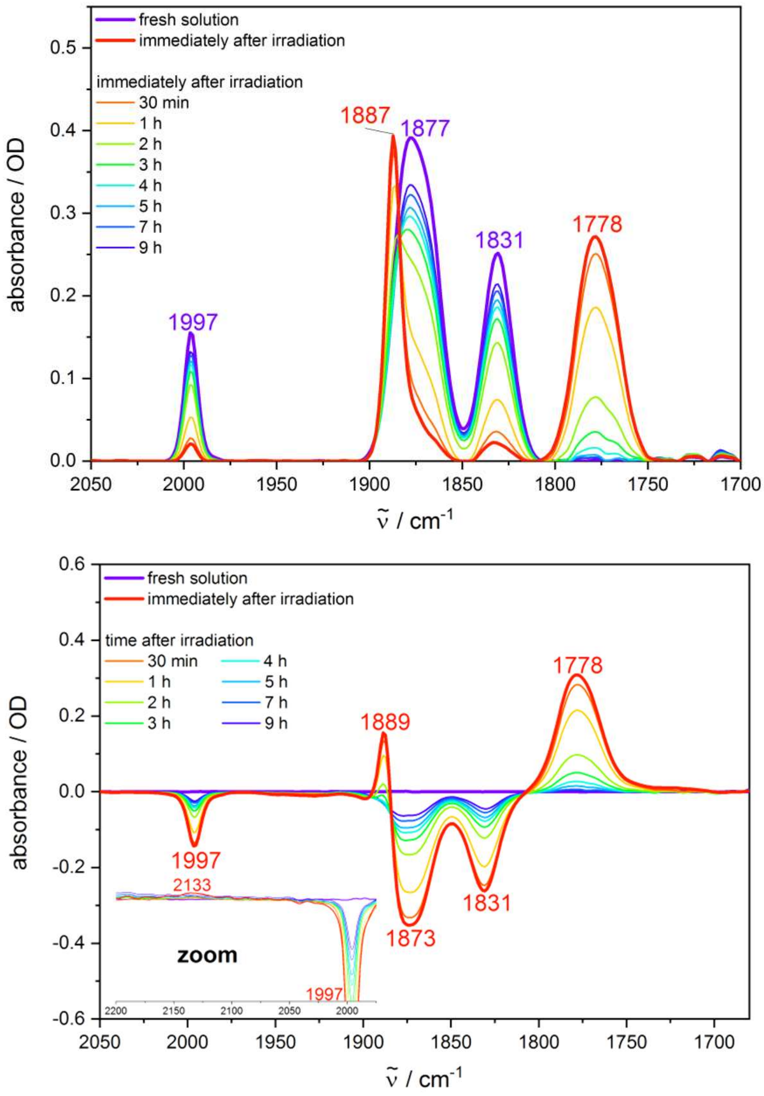

Figure S11. Irradiation (λ_{ex} = 355 nm) of a fresh solution of **W** in py (c = 6 mM) and spectra recorded in the dark after irradiation (top: absolute absorption spectra, bottom: difference spectra).

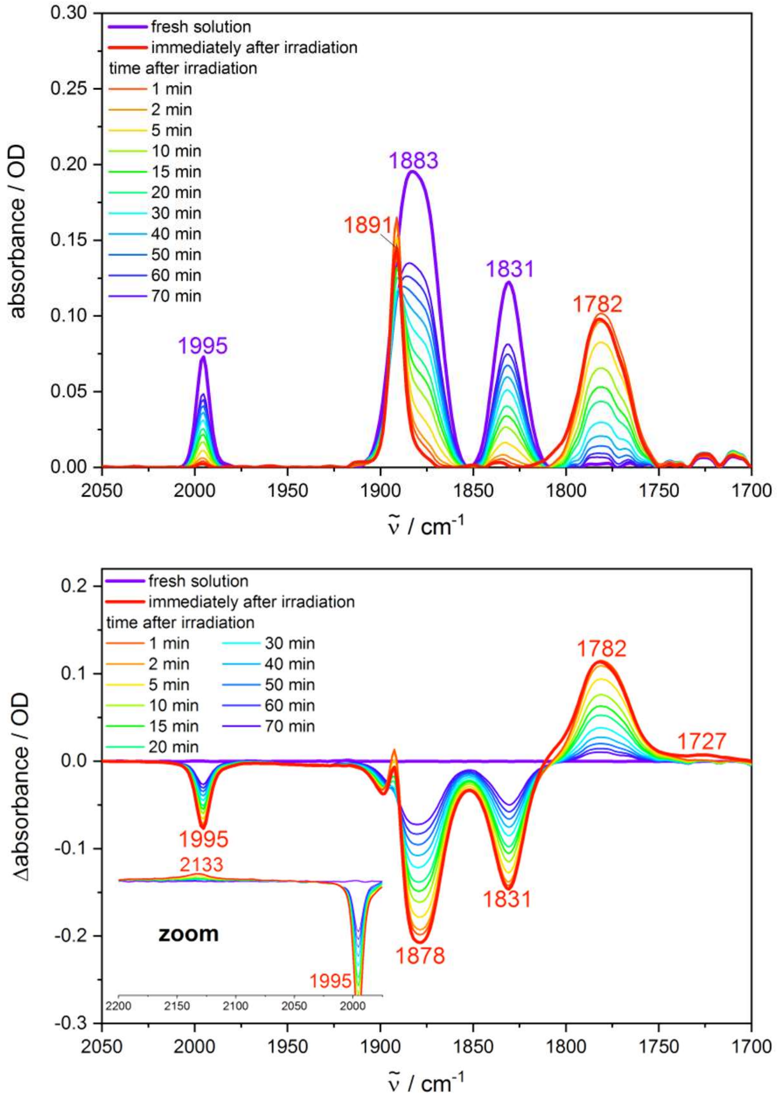

Figure S12. Irradiation (λ_{ex} = 355 nm) of a fresh solution of **Cr** in py (c = 3 mM) and spectra recorded in the dark after irradiation (top: absolute absorption spectra, bottom: difference spectra).

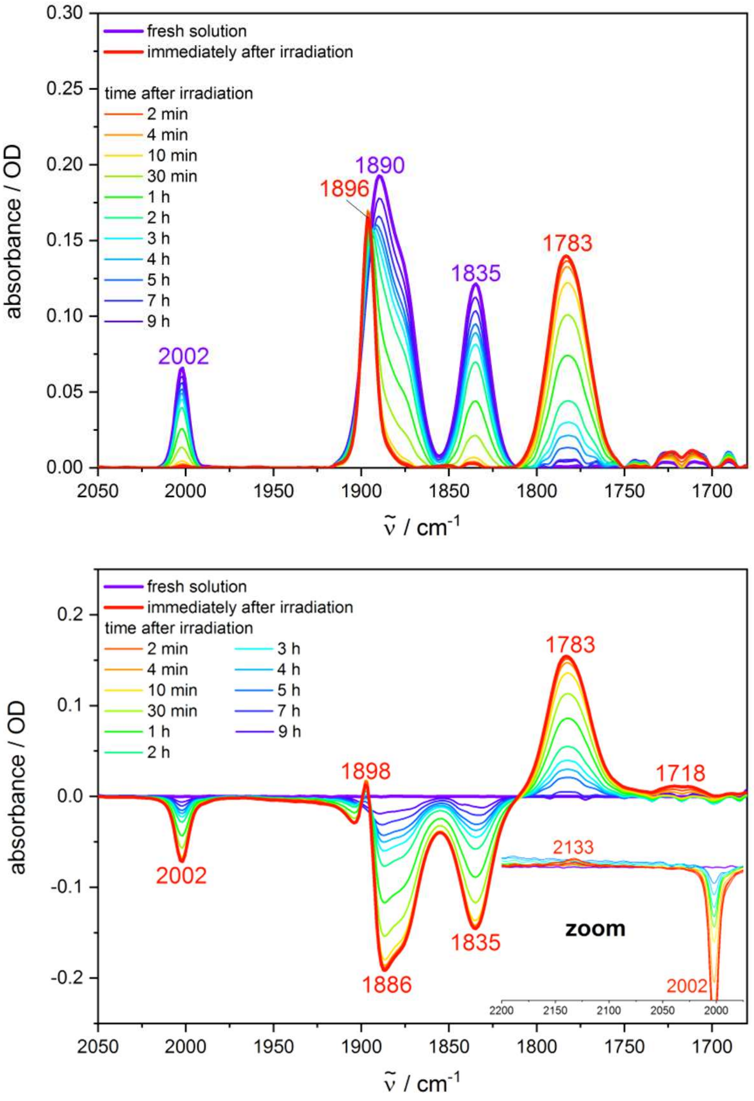

Figure S13. Irradiation (λ_{ex} = 355 nm) of a fresh solution of **Mo** in py (c = 3 mM) and spectra recorded in the dark after irradiation (top: absolute absorption spectra, bottom: difference spectra).

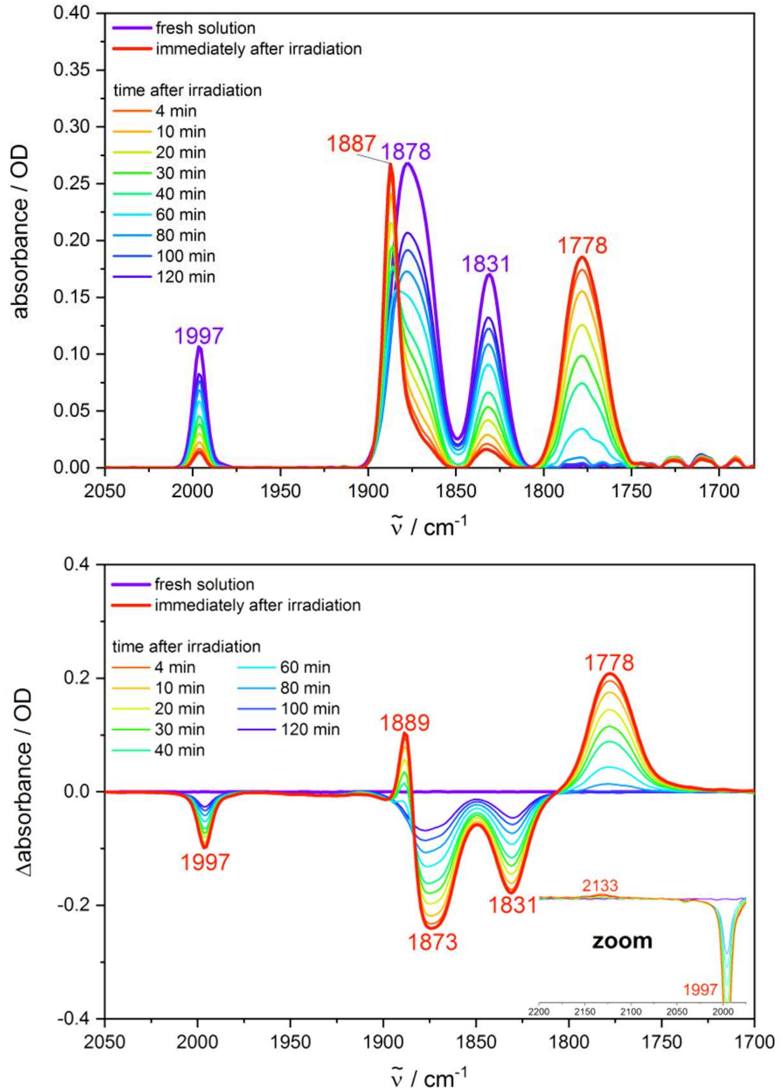

Figure S14. Irradiation (λ_{ex} = 355 nm) of a fresh solution of **W** in py (c = 3 mM) and spectra recorded in the dark after irradiation (top: absolute absorption spectra, bottom: difference spectra).

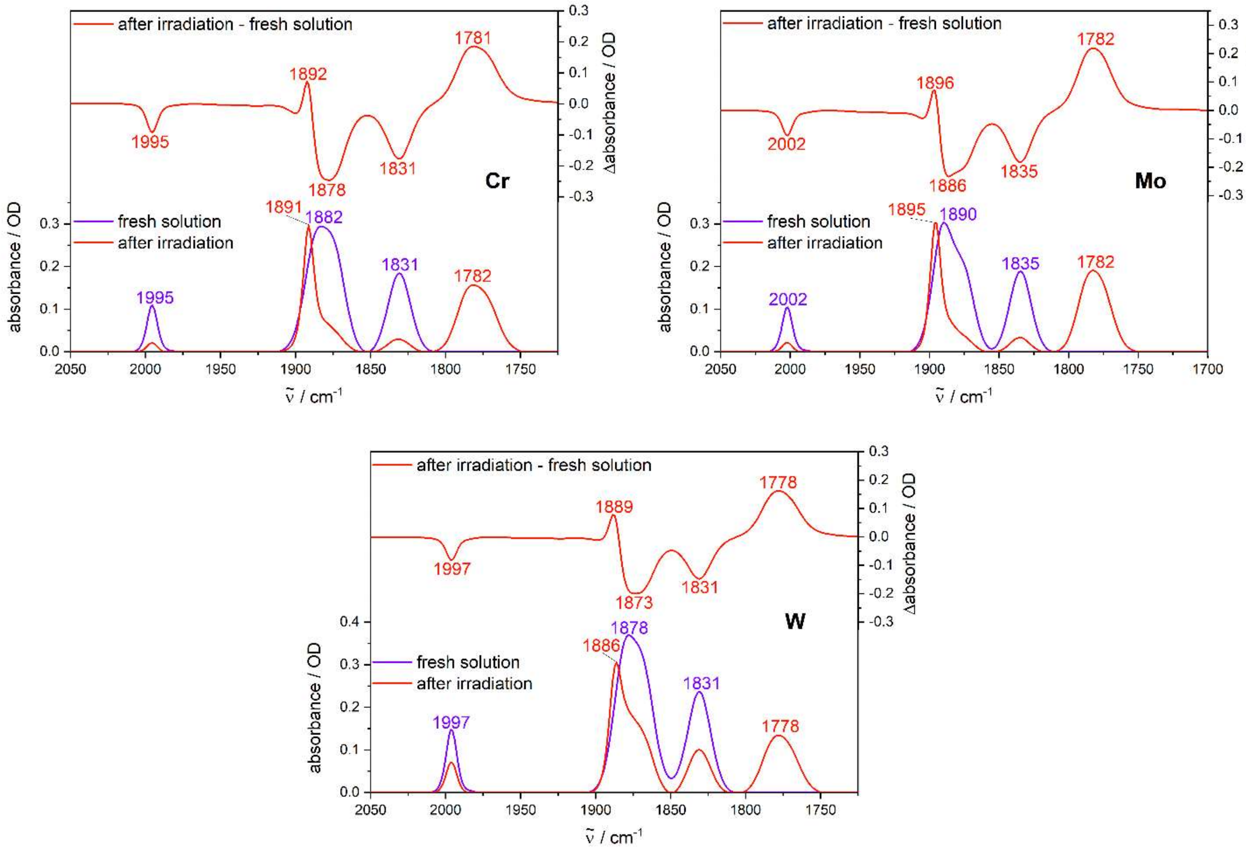

Figure S15. Irradiation (λ_{ex} = 532 nm) of fresh solutions of **Cr** (top, left), **Mo** (top, right) and **W** (bottom) in py (c = 6 mM) with representation of the absolute spectra before and immediately after irradiation as well as the corresponding difference spectrum (irradiated – fresh).

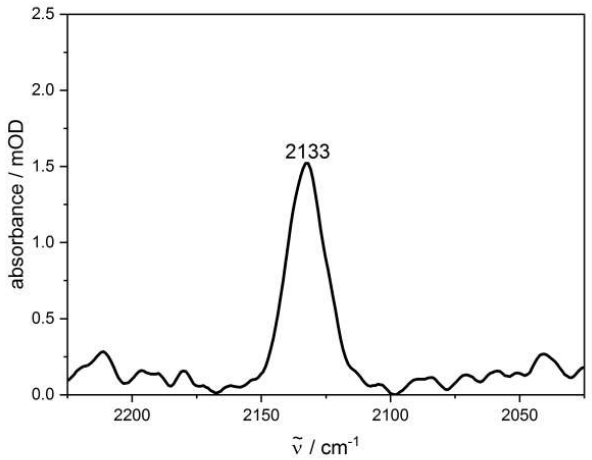

Figure S16. IR spectrum of CO dissolved in py.

2.3 Valeronitrile

Figure S17. Irradiation (λ_{ex} = 355 nm) of fresh solutions of **Cr** (top, left), **Mo** (top, right) and **W** (bottom)

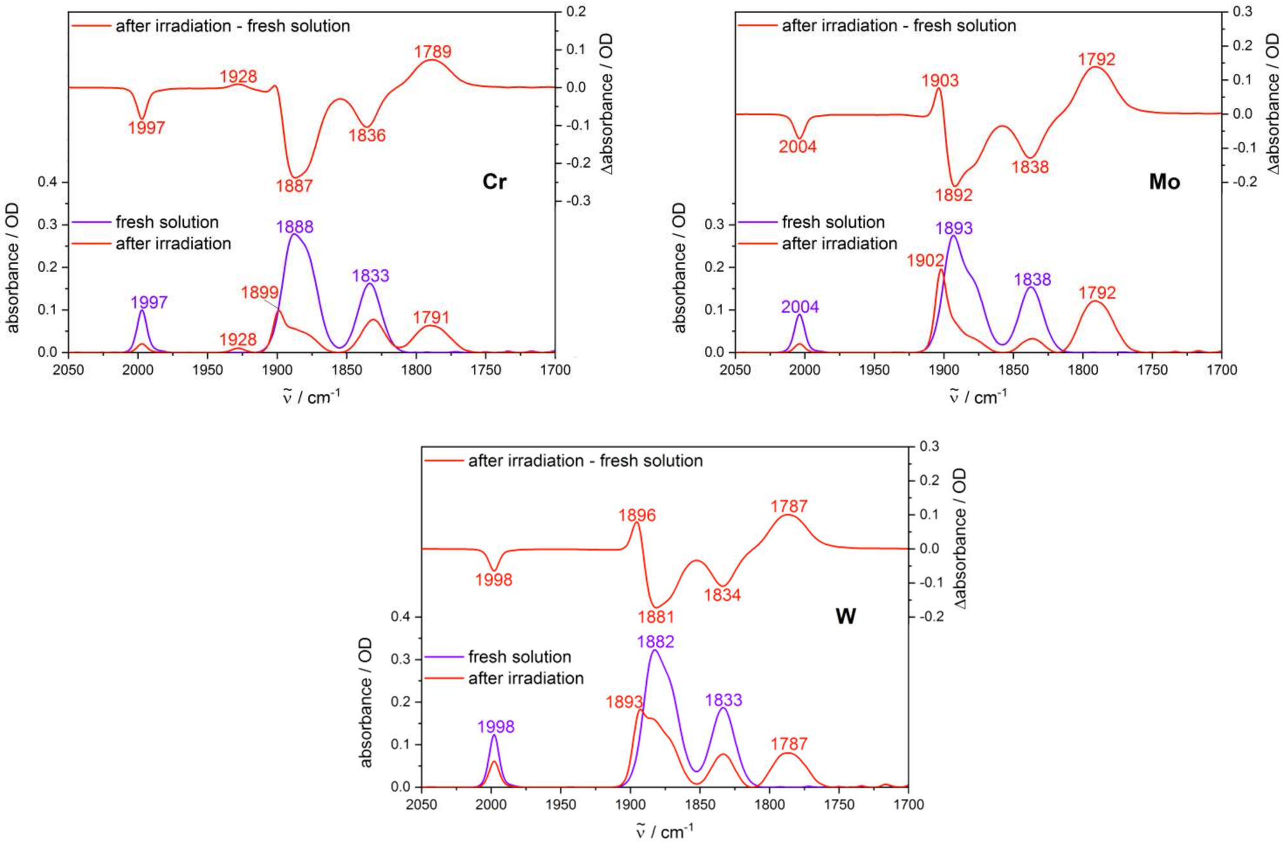

in BuCN (c = 6 mM) with representation of the absolute spectra before and immediately after irradiation as well as the corresponding difference spectrum (irradiated – fresh).

2.4 KBr matrix

Figure S18. Positive signals in the region of free CO in the difference spectra (irradiated – fresh sample)

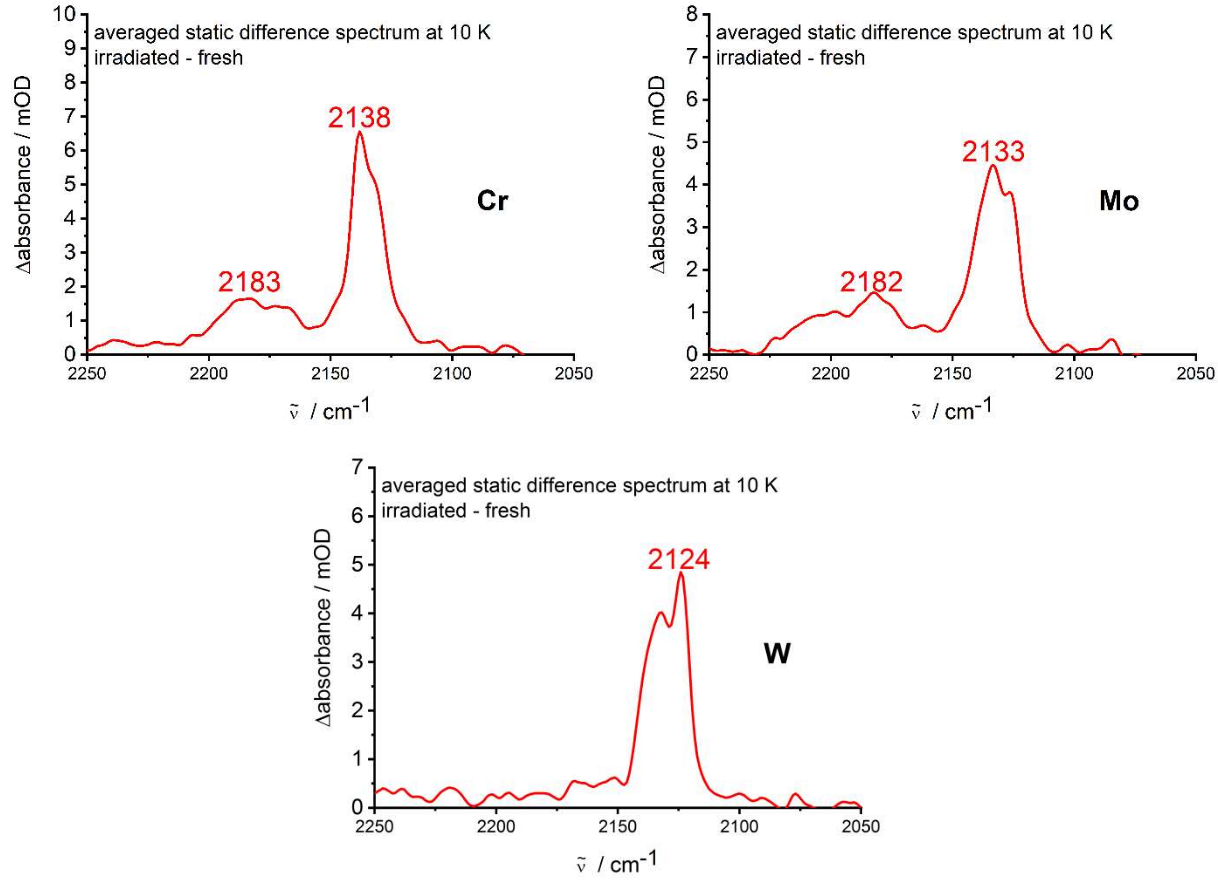

of **Cr**, **Mo** and **W** (KBr pellets) at 10 K after irradiation (λ_{ex} = 355 nm).

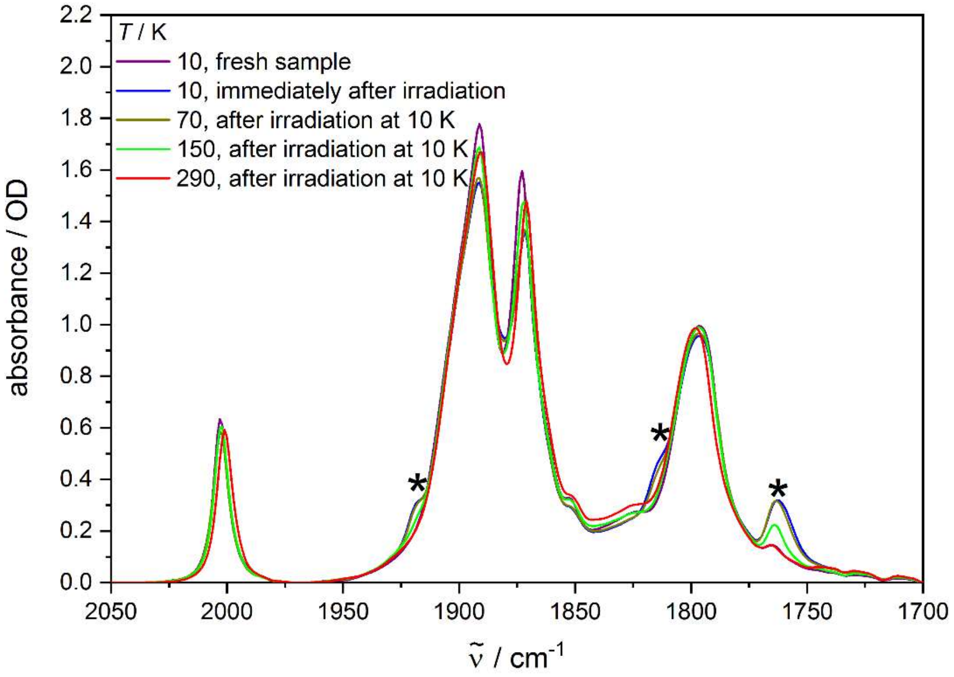

Figure S19. Irradiation (λ_{ex} = 355 nm) of a fresh sample of **Cr** (KBr pellets) at 10 K and subsequent heating to 70 K, 150 K and 290 K. Product bands are marked with asterisks.

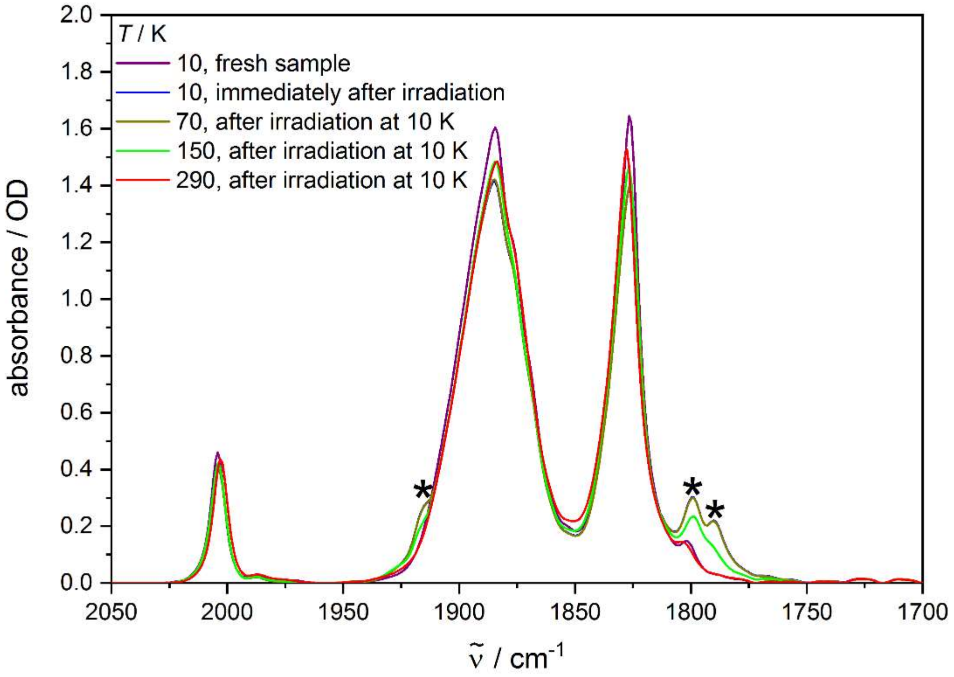

Figure S20. Irradiation (λ_{ex} = 355 nm) of a fresh sample of **Mo** (KBr pellets) at 10 K and subsequent heating to 70 K, 150 K and 290 K. Product bands are marked with asterisks.

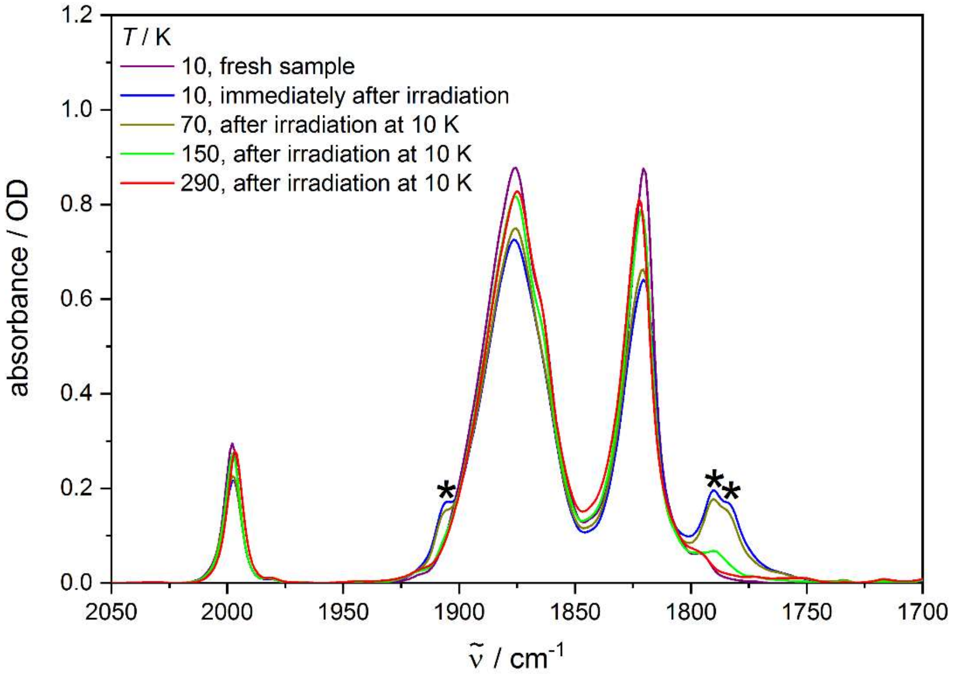

Figure S21. Irradiation (λ_{ex} = 355 nm) of a fresh sample of **W** (KBr pellets) at 10 K and subsequent heating to 70 K, 150 K and 290 K. Product bands are marked with asterisks.

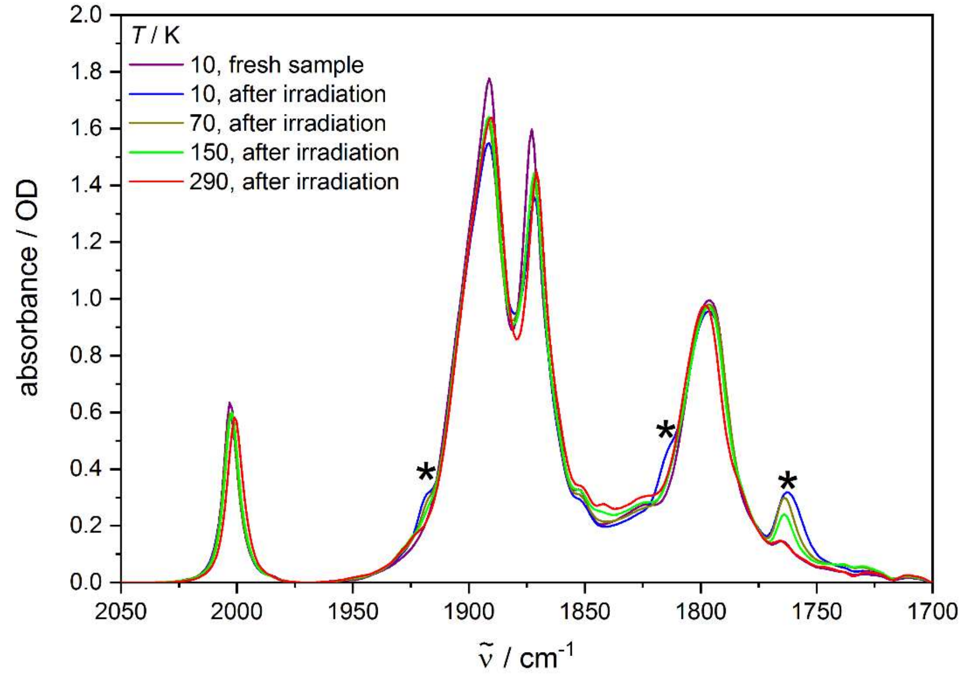

Figure S22. Irradiation (λ_{ex} = 355 nm) of fresh samples of **Cr** (KBr pellet) at 10 K, 70 K, 150 K and 290 K. Product bands are marked with asterisks.

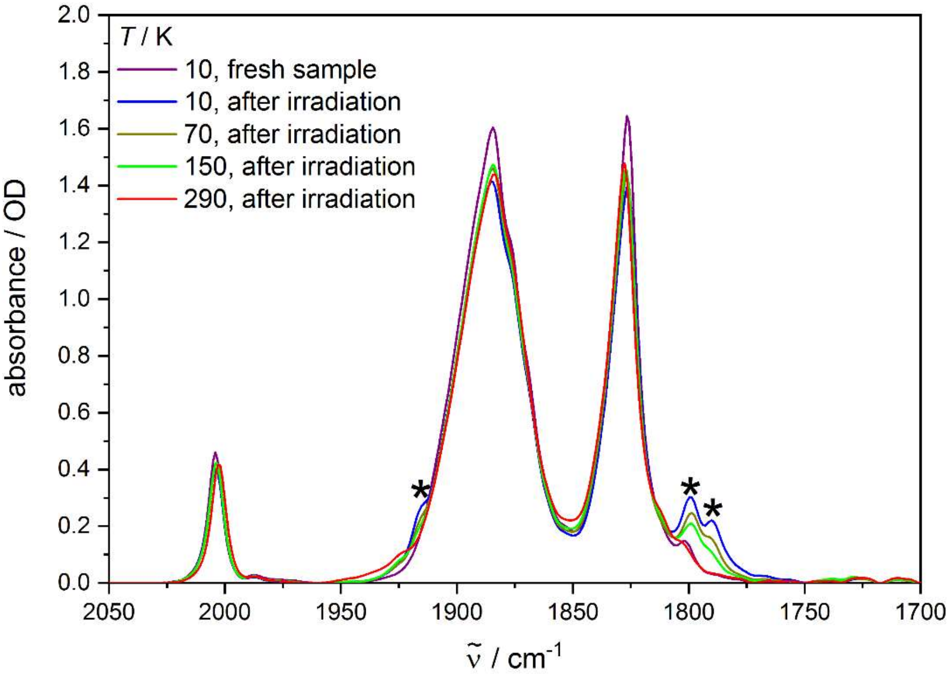

Figure S23. Irradiation (λ_{ex} = 355 nm) of fresh samples of **Mo** (KBr pellet) at 10 K, 70 K, 150 K and 290 K. Product bands are marked with asterisks.

Figure S24. Irradiation (λ_{ex} = 355 nm) of fresh samples of **W** (KBr pellet) at 10 K, 70 K, 150 K and 290 K. Product bands are marked with asterisks.

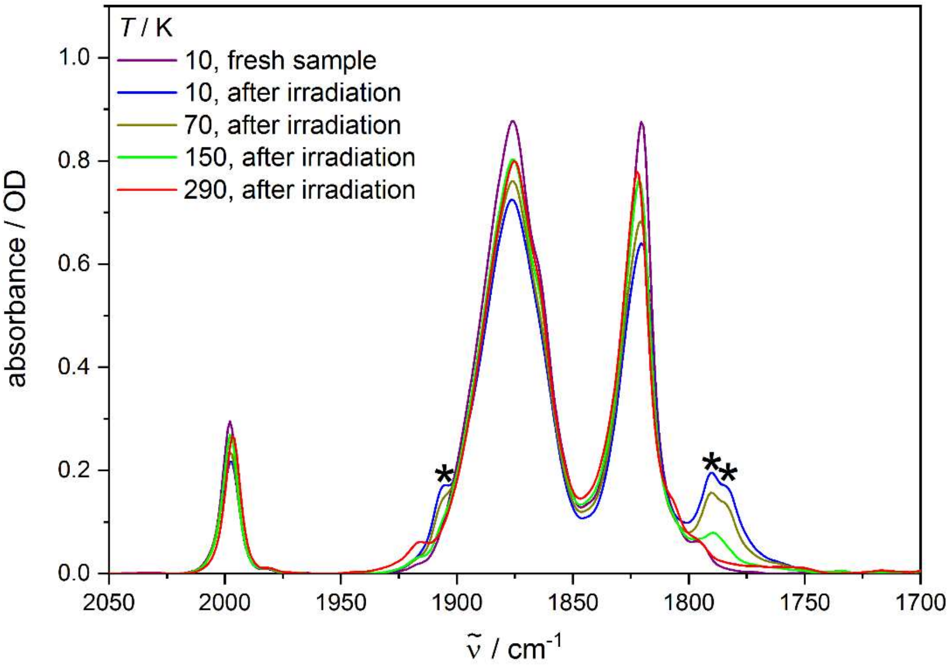

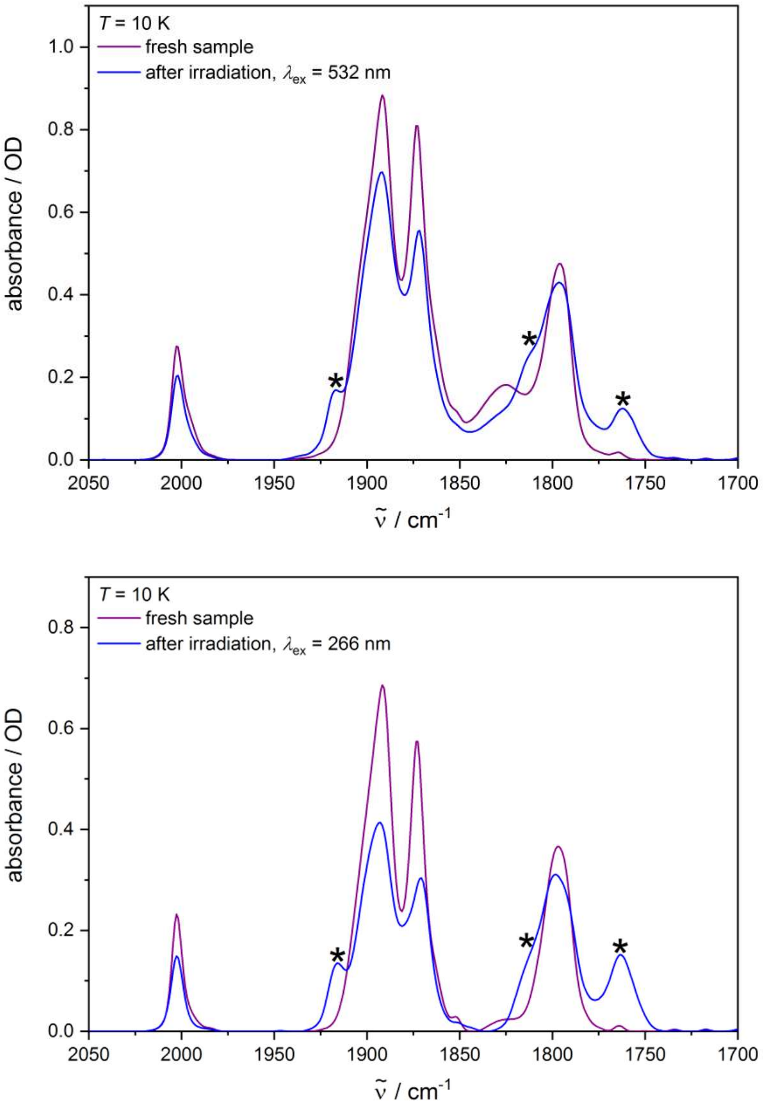

Figure S25. Irradiation of fresh samples of **Cr** (KBr pellets) at λ_{ex} = 532 nm (top) and 266 nm (bottom) at 10 K. Product bands are marked with asterisks.

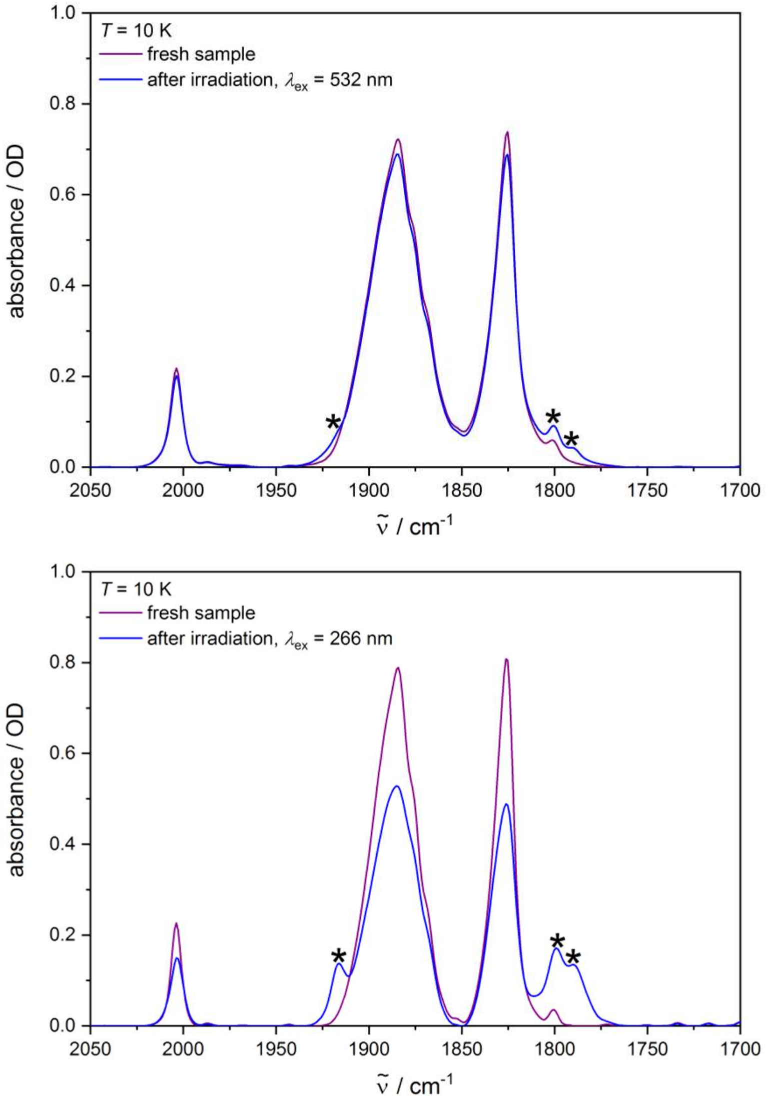

Figure S26. Irradiation of fresh samples of **Mo** (KBr pellets) at λ_{ex} = 532 nm (top) and 266 nm (bottom) at 10 K. Product bands are marked with asterisks.

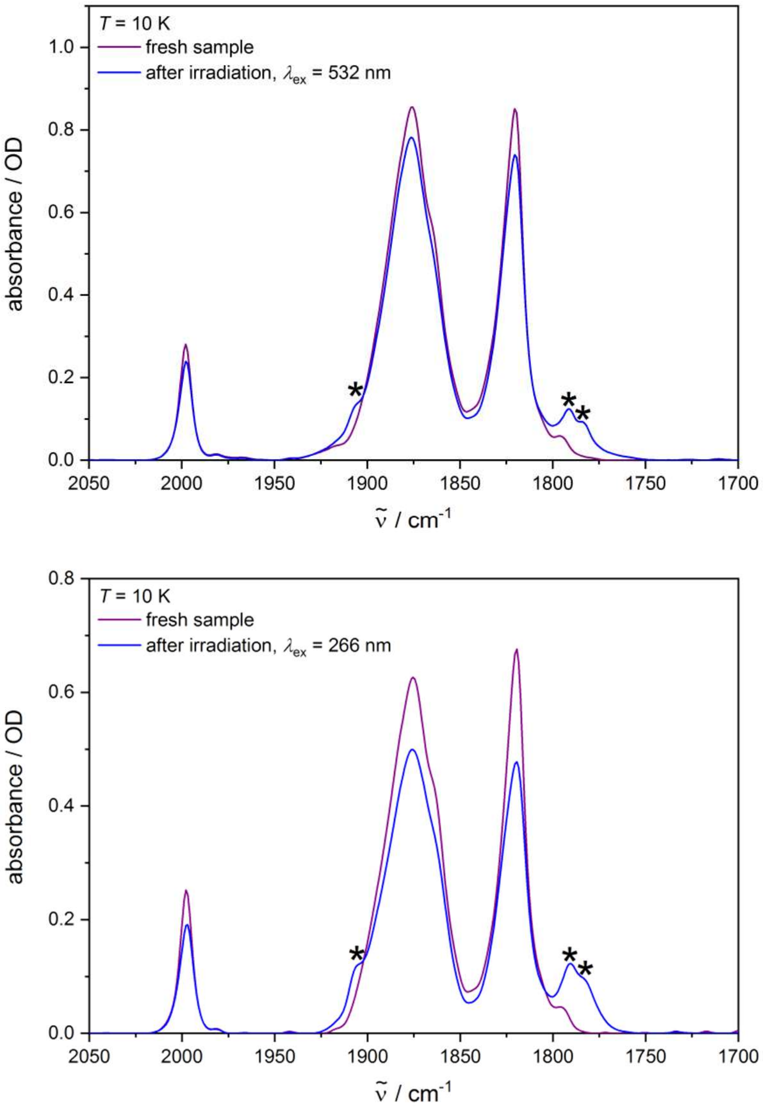

Figure S27. Irradiation of fresh samples of **W** (KBr pellets) at λ_{ex} = 532 nm (top) and 266 nm (bottom) at 10 K. Product bands are marked with asterisks.

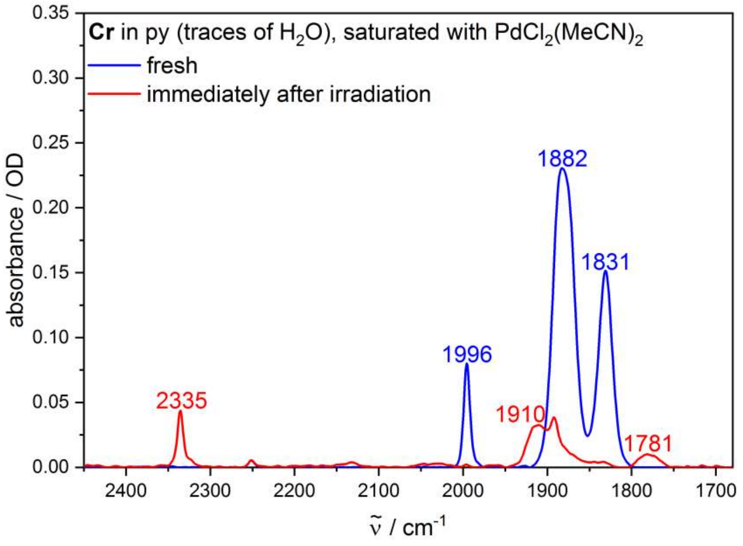

3 Detection of CO with the detection reagent $PdCl_2(MeCN)_2$

Figure S28. IR spectra of a solution of **Cr** in py (containing traces of H_2O, c = 6 mM), saturated with $PdCl_2(MeCN)_2$, before and after irradiation (λ_{ex} = 355 nm).

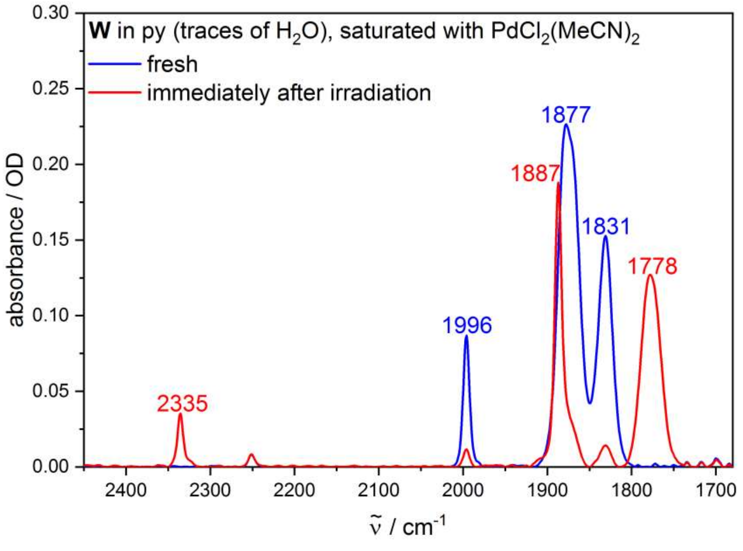

Figure S29. IR spectra of a solution of **W** in py (containing traces of H_2O, c = 6 mM), saturated with $PdCl_2(MeCN)_2$, before and after irradiation (λ_{ex} = 355 nm).

4 Comparison between experimental and theoretical IR spectra

4.1 IR spectra of Cr, Mo and W

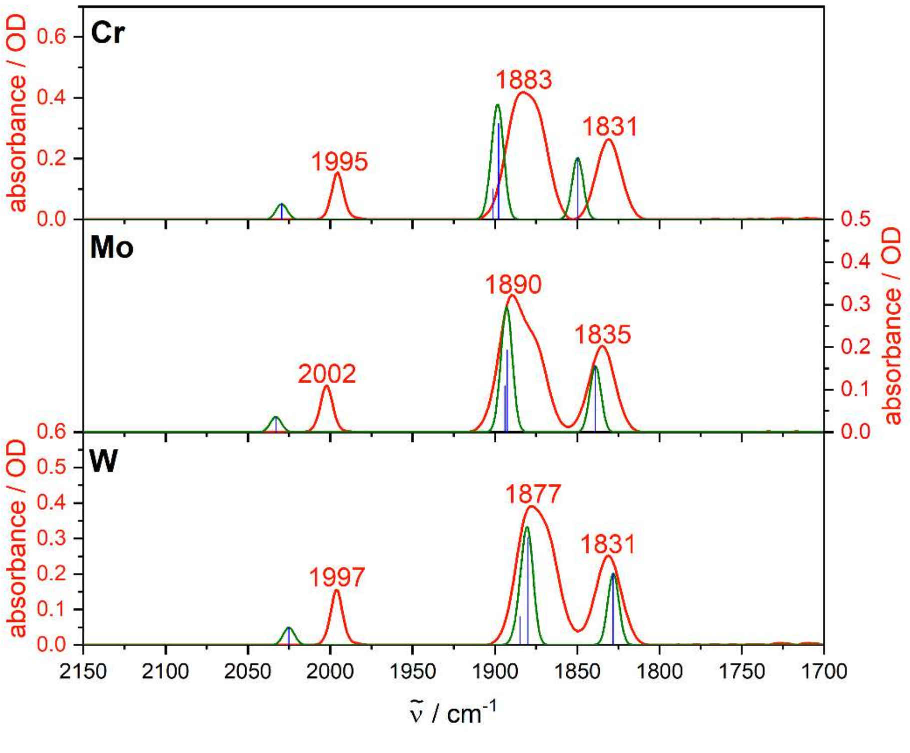

Figure S30. Experimental IR spectra of **Cr**, **Mo** and **W** in py (red) as well as calculated IR frequencies (blue) and convoluted spectra (green). Calculations: DFT/B3LYP-D3(BJ)/def2-TZVP/COSMO, scaling factor: 0.99, Gaussian convolution with FWHM=8 cm^{-1}.

4.2 Monosubstitution

4.2.1 Monosubstitution in acetonitrile

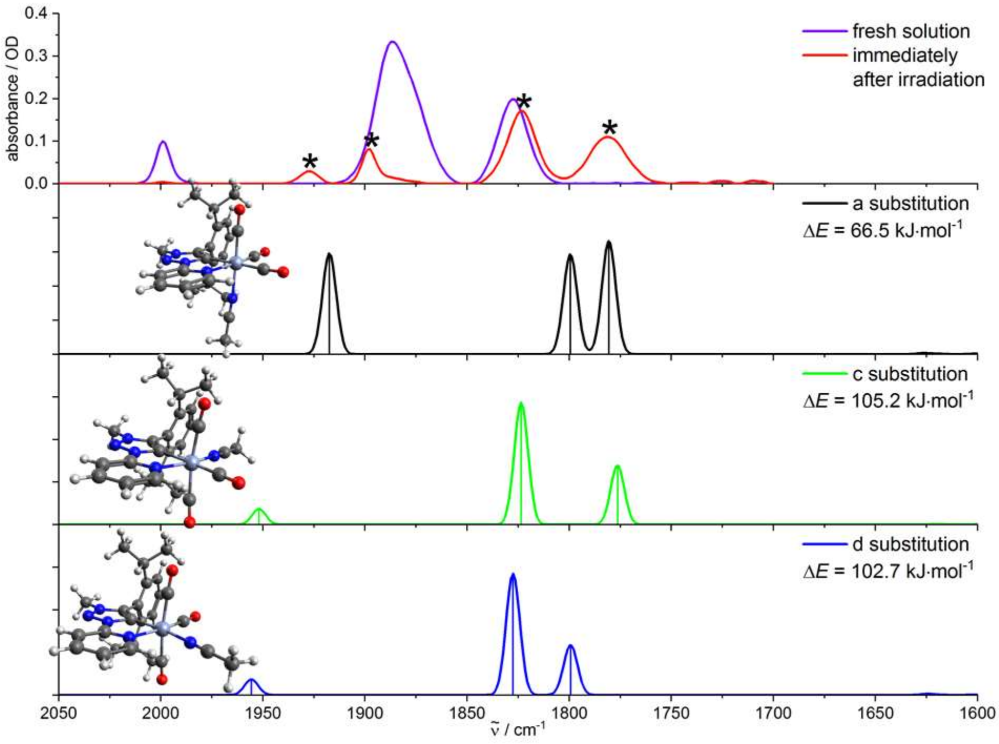

Figure S31. Experimental IR spectra of **Cr** in MeCN before and after irradiation (upper trace, bands marked with asterisks result from the formed photoproduct(s)) and calculated IR spectra of the photoproducts with a substitution of a CO ligand for a MeCN molecule (three lower traces), including the optimized structures and calculated enthalpies of reaction. The substitution occurs in axial position for isomer a ([LCr(CO)$_3$MeCN$_{ax}$]) and in equatorial position for isomers c/d ([LMo(CO)$_3$MeCN$_{eq}$]), respectively. Calculations: DFT/B3LYP-D3(BJ)/def2-TZVP/COSMO, scaling factor: 0.99, Gaussian convolution with FWHM=8 cm^{-1}.

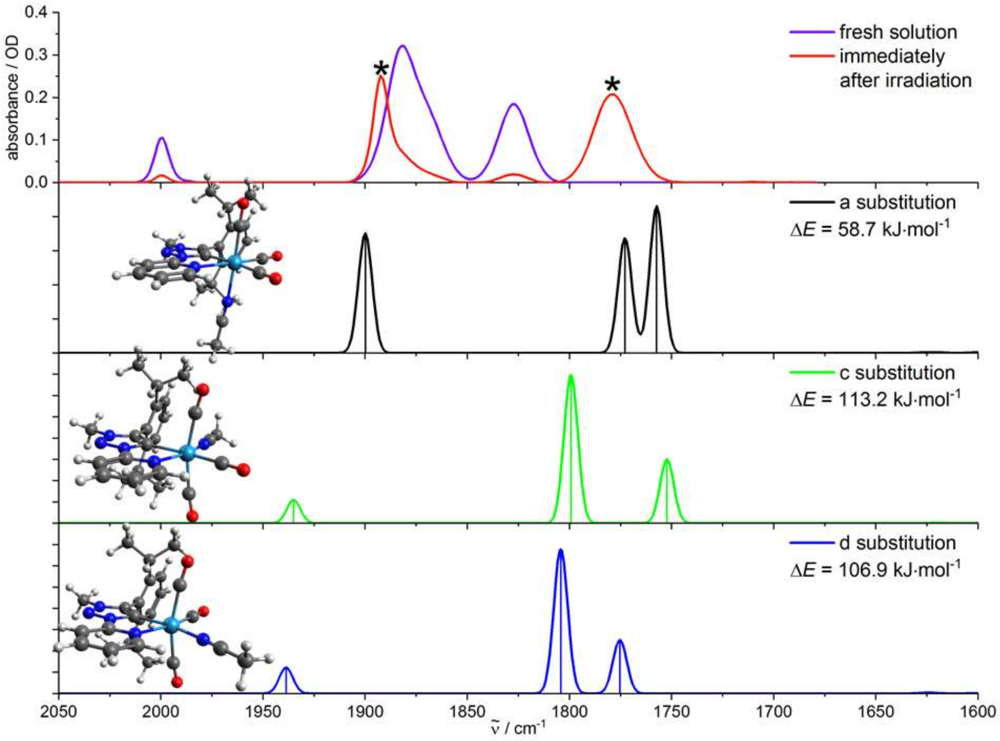

Figure S32. Experimental IR spectra of **W** in MeCN before and after irradiation (upper trace, bands marked with asterisks result from the formed photoproduct(s)) and calculated IR spectra of the photoproducts with a substitution of a CO ligand for a MeCN molecule (three lower traces), including the optimized structures and calculated enthalpies of reaction. The substitution occurs in axial position for isomer a ($[LW(CO)_3MeCN_{ax}]$) and in equatorial position for isomers c/d ($[LW(CO)_3MeCN_{eq}]$), respectively. Calculations: DFT/B3LYP-D3(BJ)/def2-TZVP/COSMO, scaling factor: 0.99, Gaussian convolution with FWHM=8 cm^{-1}.

4.2.2 Monosubstitution in pyridine

Figure S33. Experimental IR spectra of **Cr** in py before and after irradiation (upper trace, bands marked

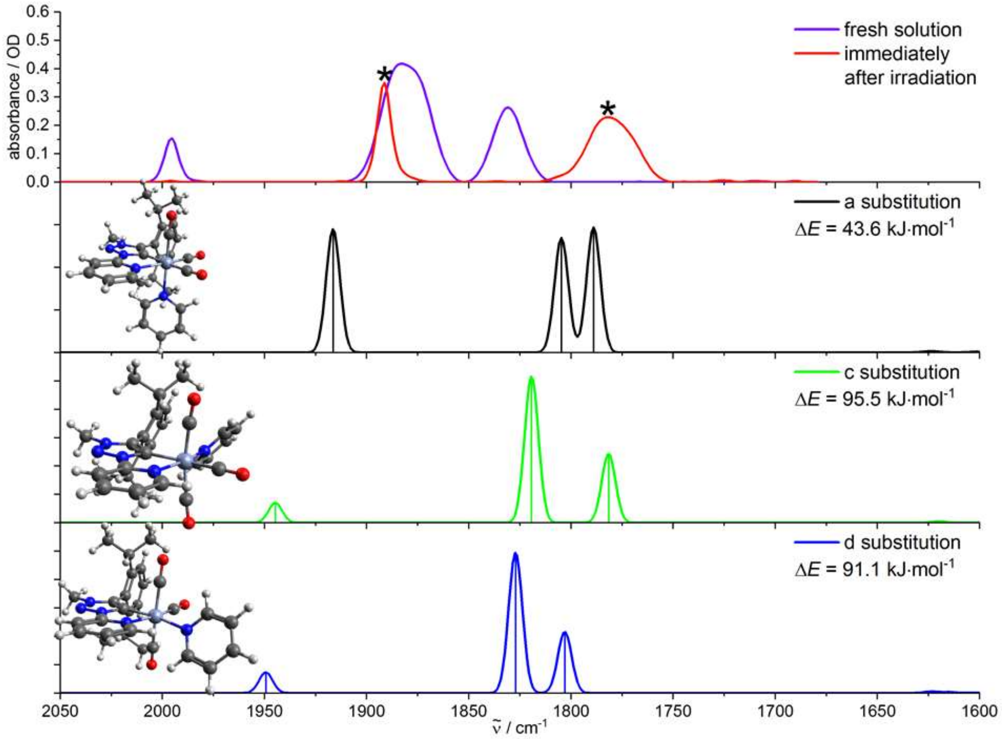

with asterisks result from the formed photoproduct(s)) and calculated IR spectra of the photoproducts with a substitution of a CO ligand for a py molecule (three lower traces), including the optimized structures and calculated enthalpies of reaction. The substitution occurs in axial position for isomer a ($[LCr(CO)_3py_{ax}]$) and in equatorial position for isomers c/d ($[LCr(CO)_3py_{eq}]$), respectively. Calculations: DFT/B3LYP-D3(BJ)/def2-TZVP/COSMO, scaling factor: 0.99, Gaussian convolution with FWHM=8 cm^{-1}.

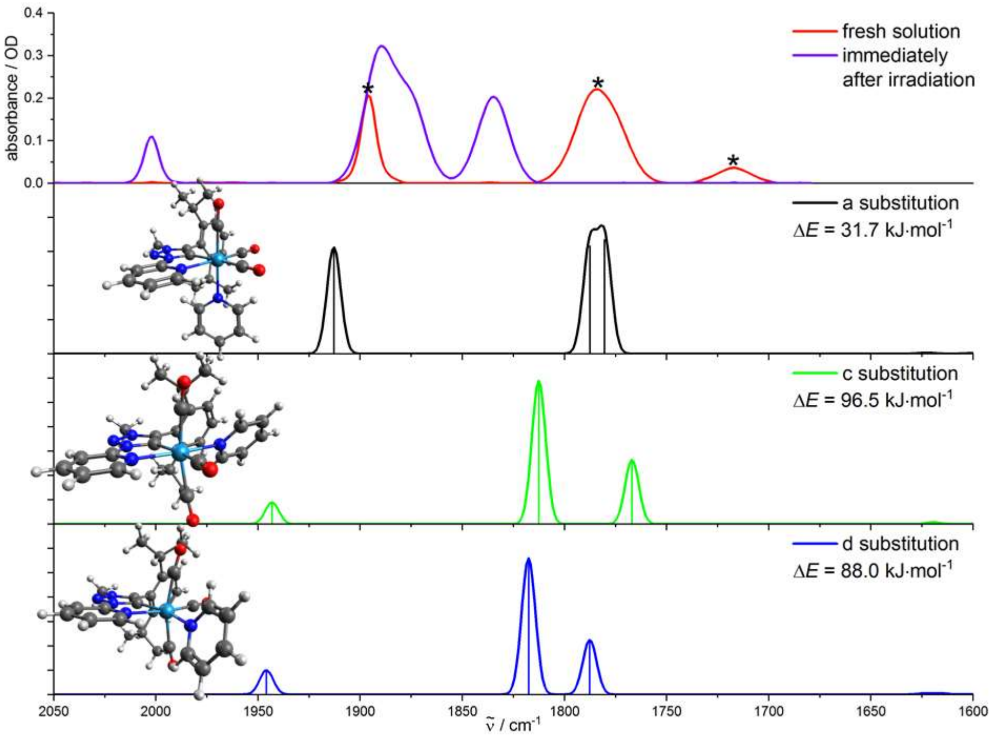

Figure S34. Experimental IR spectra of **Mo** in py before and after irradiation (upper trace, bands marked with asterisks result from the formed photoproduct(s)) and calculated IR spectra of the photoproducts with a substitution of a CO ligand for a py molecule (three lower traces), including the optimized structures and calculated enthalpies of reaction. The substitution occurs in axial position for isomer a ([LMo(CO)$_3$py$_{ax}$]) and in equatorial position for isomers c/d ([LMo(CO)$_3$py$_{eq}$]), respectively. Calculations: DFT/B3LYP-D3(BJ)/def2-TZVP/COSMO, scaling factor: 0.99, Gaussian convolution with FWHM=8 cm^{-1}.

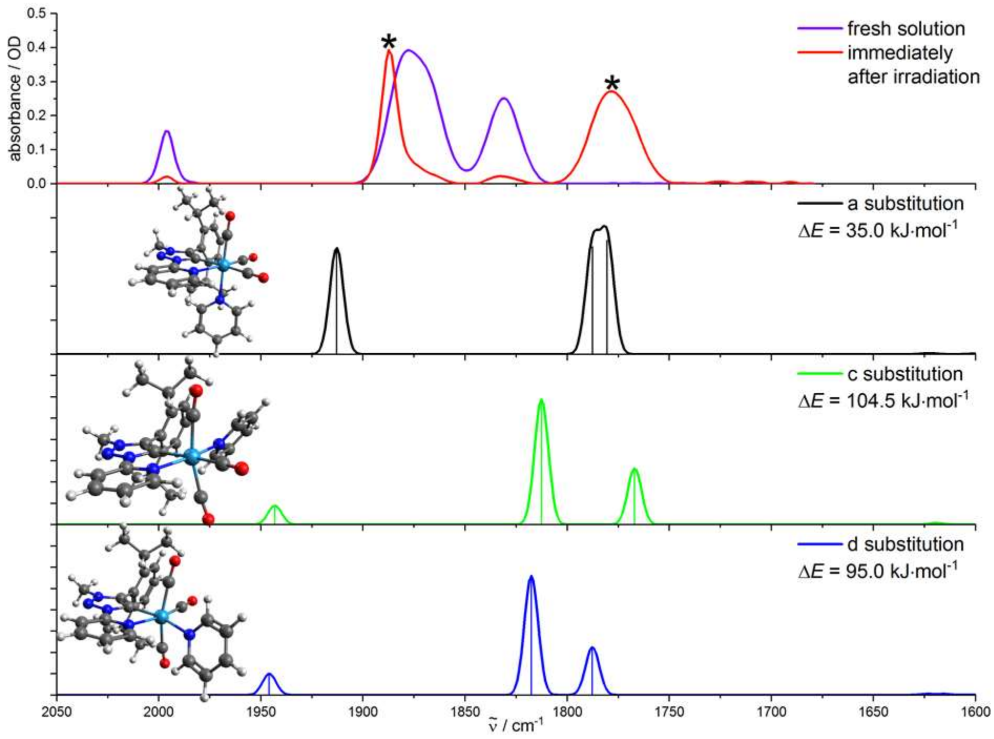

Figure S35. Experimental IR spectra of **W** in py before and after irradiation (upper trace, bands marked with asterisks result from the formed photoproduct(s)) and calculated IR spectra of the photoproducts with a substitution of a CO ligand for a py molecule (three lower traces), including the optimized structures and calculated enthalpies of reaction. The substitution occurs in axial position for isomer a ($[LW(CO)_3py_{ax}]$) and in equatorial position for isomers c/d ($[LW(CO)_3py_{eq}]$), respectively. Calculations: DFT/B3LYP-D3(BJ)/def2-TZVP/COSMO, scaling factor: 0.99, Gaussian convolution with FWHM=8 cm^{-1}.

4.3 Bisubstitution

4.3.1 Bisubstitution in acetonitrile

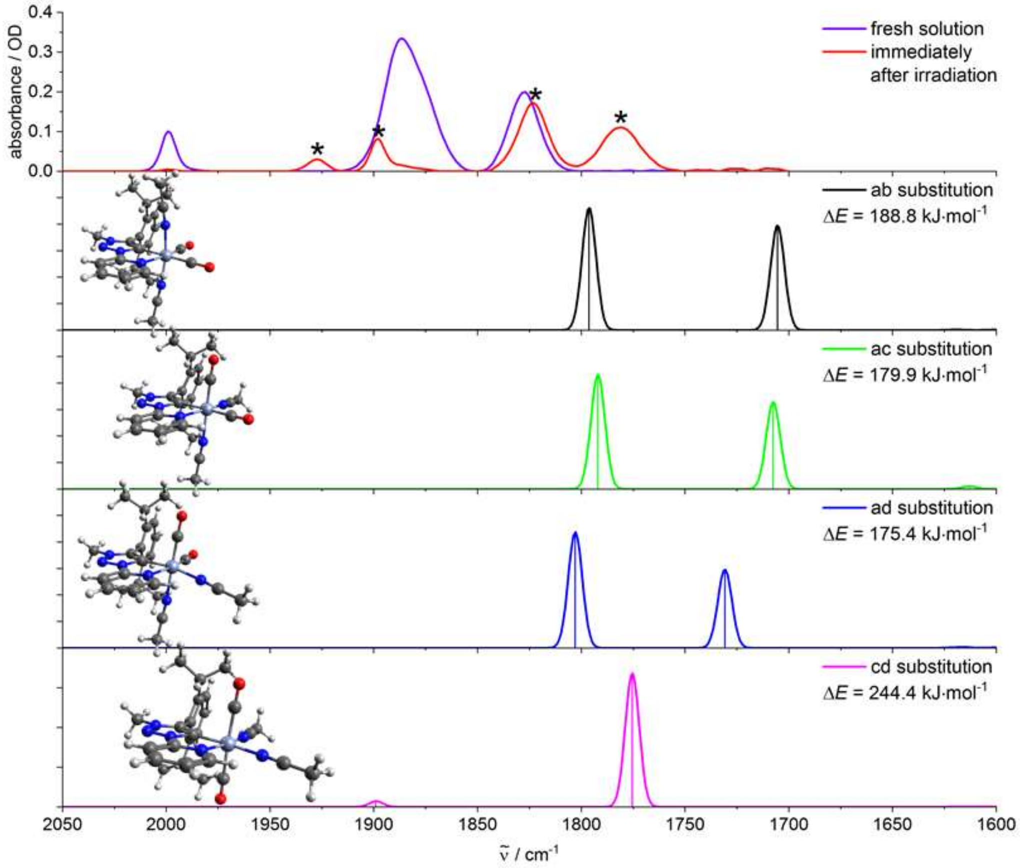

Figure S36. Experimental IR spectra of **Cr** in MeCN before and after irradiation (upper trace, bands marked with asterisks result from the formed photoproduct(s)) and calculated IR spectra of the photoproducts with a substitution of two CO ligands for two MeCN molecules (three lower traces), including the optimized structures and calculated enthalpies of reaction. The bisubstitution occurs in the two axial positions for isomer ab ($[LCr(CO)_3(MeCN_{ax})_2]$), in an axial and an equatorial position for isomers ac/ad ($[LCr(CO)_3(MeCN_{ax})(MeCN_{eq})]$) and in the two equatorial positions for isomer cd ($[LCr(CO)_3(MeCN_{eq})_2]$), respectively. Calculations: DFT/B3LYP-D3(BJ)/def2-TZVP/COSMO, scaling factor: 0.99, Gaussian convolution with FWHM=8 cm^{-1}.

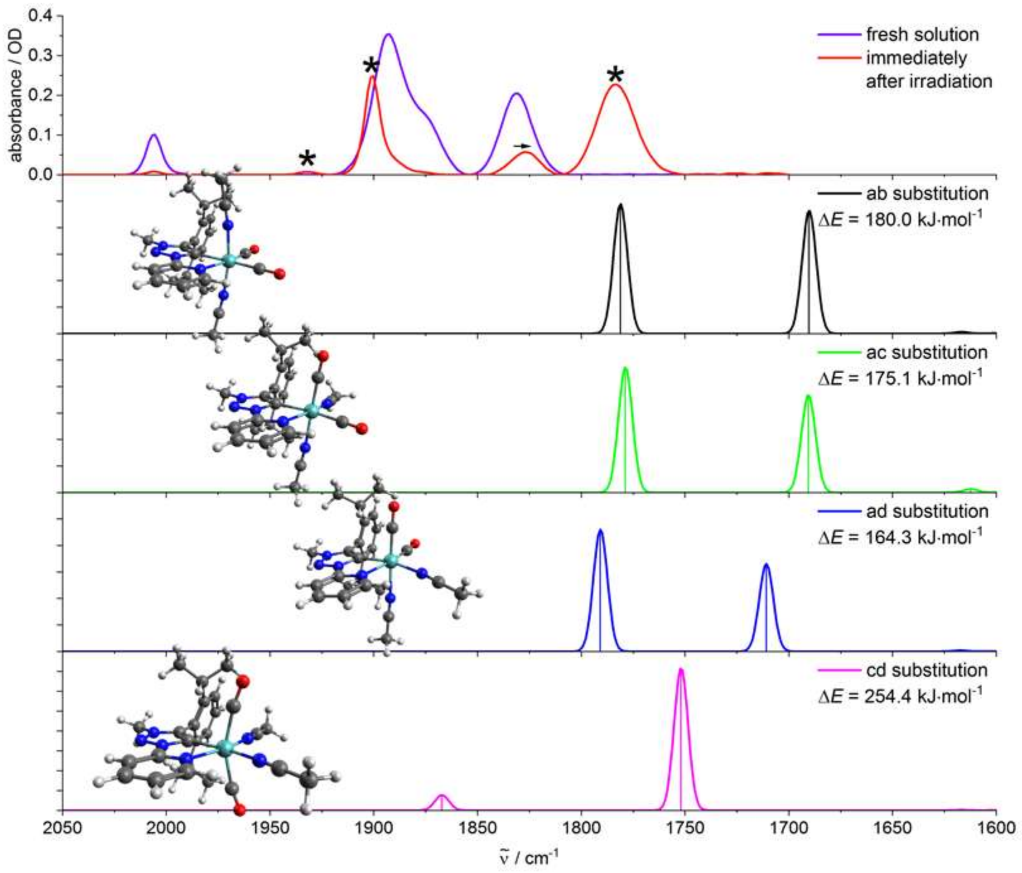

Figure S37. Experimental IR spectra of **Mo** in MeCN before and after irradiation (upper trace, bands marked with asterisks result from the formed photoproduct(s)) and calculated IR spectra of the photoproducts with a substitution of two CO ligands for two MeCN molecules (three lower traces), including the optimized structures and calculated enthalpies of reaction. The bisubstitution occurs in the two axial positions for isomer ab ($[LMo(CO)_3(MeCN_{ax})_2]$), in an axial and an equatorial position for isomers ac/ad ($[LMo(CO)_3(MeCN_{ax})(MeCN_{eq})]$) and in the two equatorial positions for isomer cd ($[LMo(CO)_3(MeCN_{eq})_2]$), respectively. Calculations: DFT/B3LYP-D3(BJ)/def2-TZVP/COSMO, scaling factor: 0.99, Gaussian convolution with FWHM=8 cm^{-1}.

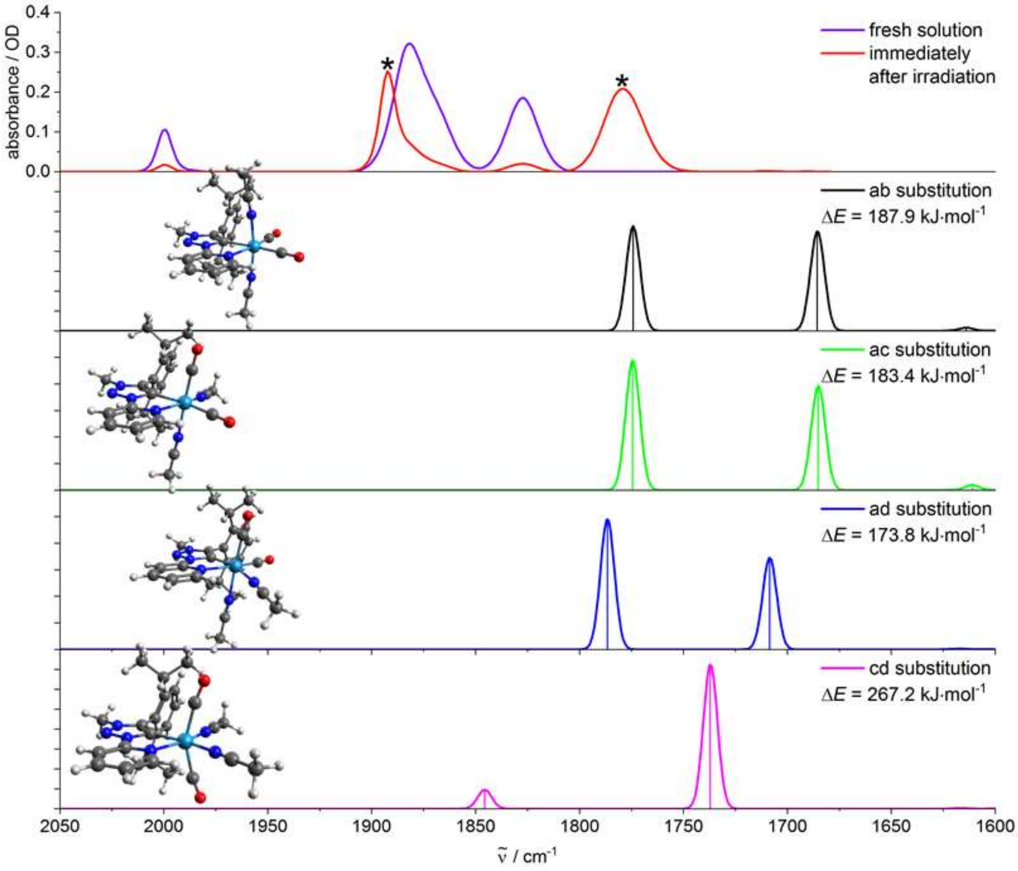

Figure S38. Experimental IR spectra of **W** in MeCN before and after irradiation (upper trace, bands marked with asterisks result from the formed photoproduct(s)) and calculated IR spectra of the photoproducts with a substitution of two CO ligands for two MeCN molecules (three lower traces), including the optimized structures and calculated enthalpies of reaction. The bisubstitution occurs in the two axial positions for isomer ab ($[LW(CO)_3(MeCN_{ax})_2]$), in an axial and an equatorial position for isomers ac/ad ($[LW(CO)_3(MeCN_{ax})(MeCN_{eq})]$) and in the two equatorial positions for isomer cd ($[LW(CO)_3(MeCN_{eq})_2]$), respectively. Calculations: DFT/B3LYP-D3(BJ)/def2-TZVP/COSMO, scaling factor: 0.99, Gaussian convolution with FWHM=8 cm^{-1}.

4.3.2 Bisubstitution in pyridine

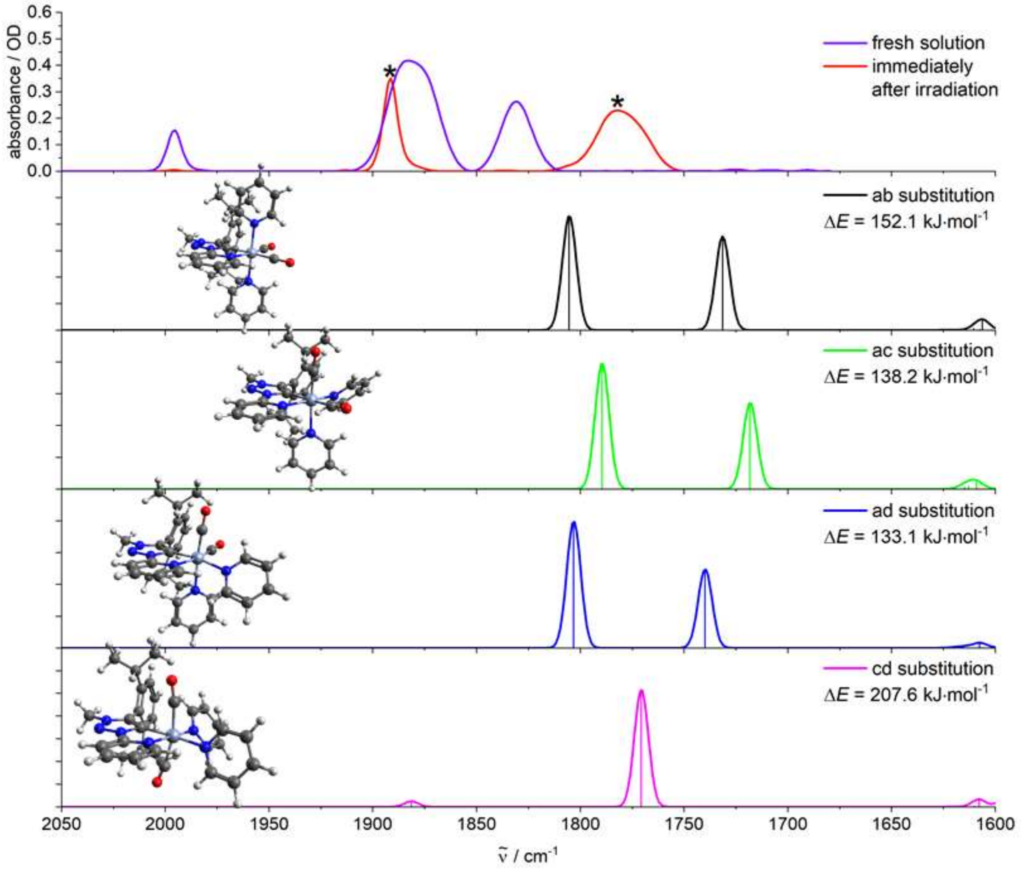

Figure S39. Experimental IR spectra of **Cr** in py before and after irradiation (upper trace, bands marked with asterisks result from the formed photoproduct(s)) and calculated IR spectra of the photoproducts with a substitution of two CO ligands for two py molecules (three lower traces), including the optimized structures and calculated enthalpies of reaction. The bisubstitution occurs in the two axial positions for isomer ab ($[LCr(CO)_3(py_{ax})_2]$), in an axial and an equatorial position for isomers ac/ad ($[LCr(CO)_3(py_{ax})(py_{eq})]$) and in the two equatorial positions for isomer cd ($[LCr(CO)_3(py_{eq})_2]$), respectively. Calculations: DFT/B3LYP-D3(BJ)/def2-TZVP/COSMO, scaling factor: 0.99, Gaussian convolution with FWHM=8 cm^{-1}.

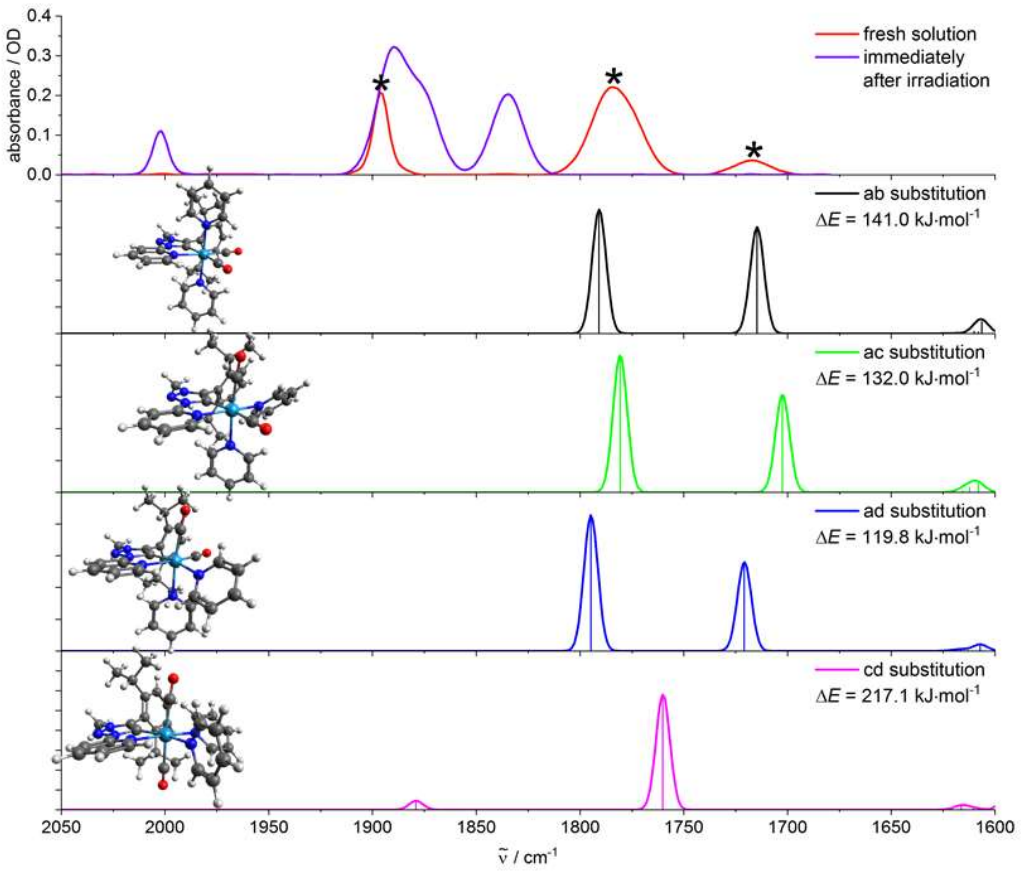

Figure S40. Experimental IR spectra of **Mo** in py before and after irradiation (upper trace, bands marked with asterisks result from the formed photoproduct(s)) and calculated IR spectra of the photoproducts with a substitution of two CO ligands for two py molecules (three lower traces), including the optimized structures and calculated enthalpies of reaction. The bisubstitution occurs in the two axial positions for isomer ab ($[LMo(CO)_3(py_{ax})_2]$), in an axial and an equatorial position for isomers ac/ad ($[LMo(CO)_3(py_{ax})(py_{eq})]$) and in the two equatorial positions for isomer cd ($[LMo(CO)_3(py_{eq})_2]$), respectively. Calculations: DFT/B3LYP-D3(BJ)/def2-TZVP/COSMO, scaling factor: 0.99, Gaussian convolution with FWHM=8 cm^{-1}.

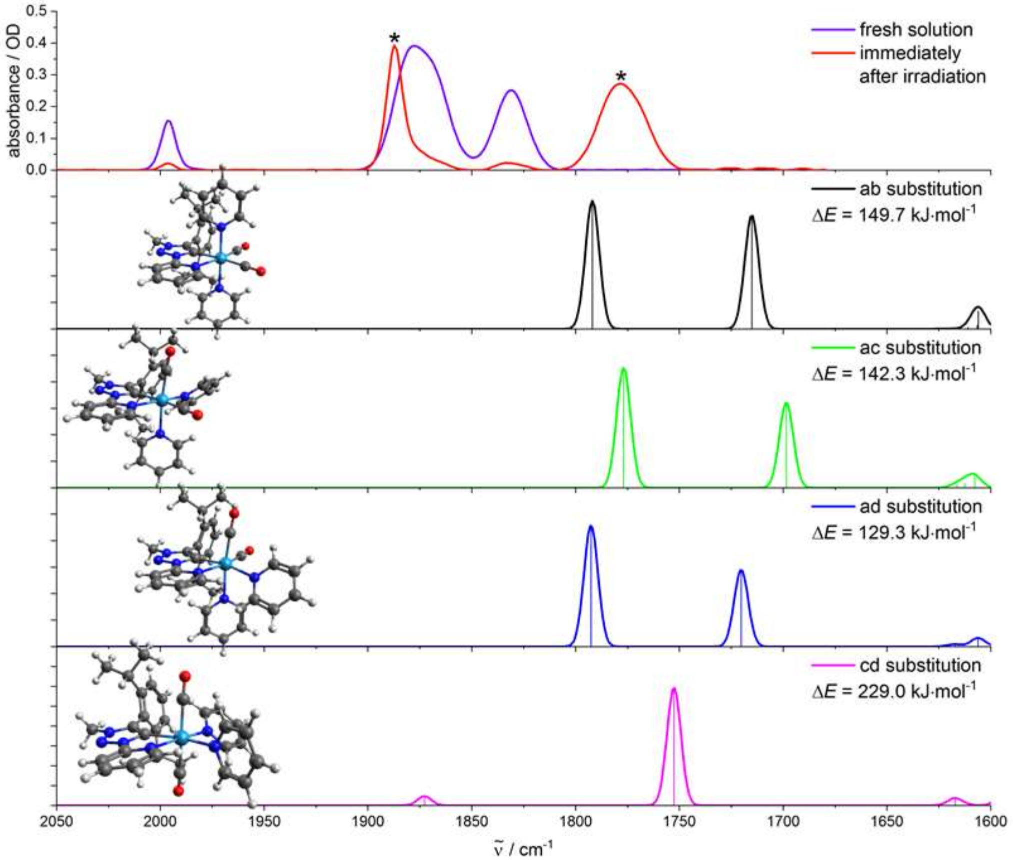

Figure S41. Experimental IR spectra of **W** in py before and after irradiation (upper trace, bands marked with asterisks result from the formed photoproduct(s)) and calculated IR spectra of the photoproducts with a substitution of two CO ligands for two py molecules (three lower traces), including the optimized structures and calculated enthalpies of reaction. The bisubstitution occurs in the two axial positions for isomer ab ($[LW(CO)_3(py_{ax})_2]$), in an axial and an equatorial position for isomers ac/ad ($[LW(CO)_3(py_{ax})(py_{eq})]$) and in the two equatorial positions for isomer cd ($[LW(CO)_3(py_{eq})_2]$), respectively. Calculations: DFT/B3LYP-D3(BJ)/def2-TZVP/COSMO, scaling factor: 0.99, Gaussian convolution with FWHM=8 cm^{-1}.

4.4 Trisubstitution

4.4.1 Trisubstitution in acetonitrile

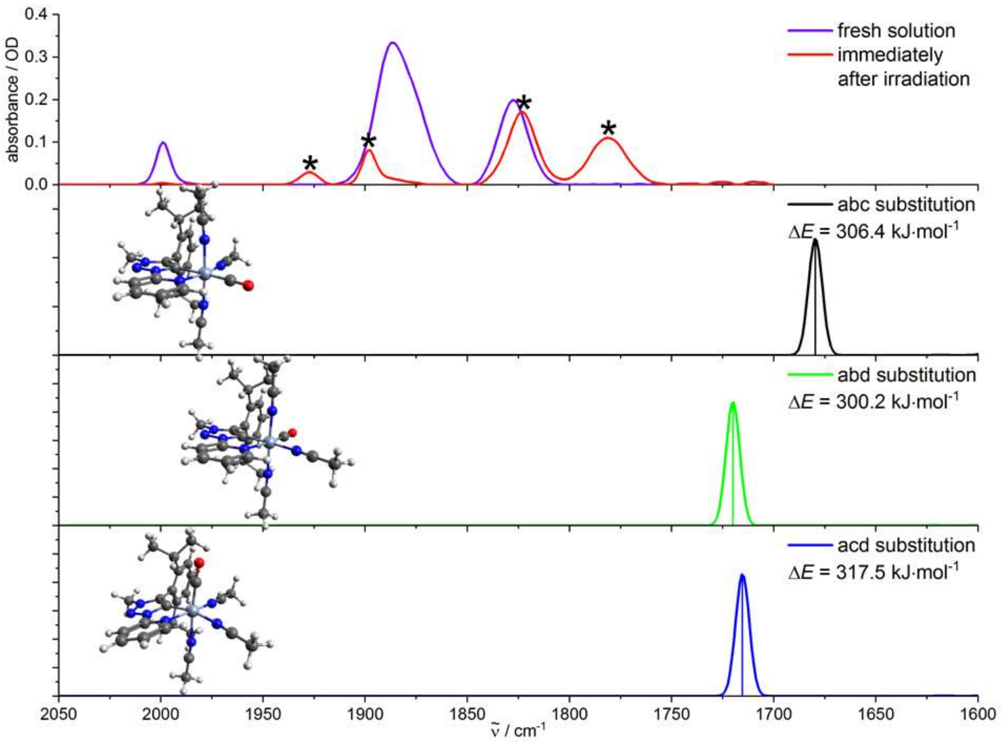

Figure S42. Experimental IR spectra of **Cr** in MeCN before and after irradiation (upper trace, bands marked with asterisks result from the formed photoproduct(s)) and calculated IR spectra of the photoproducts with a substitution of three CO ligands for three MeCN molecules (three lower traces), including the optimized structures and calculated enthalpies of reaction. A coordinated CO ligand remains in equatorial position for isomers abc/abd and in axial position for isomer acd. Calculations: DFT/B3LYP-D3(BJ)/def2-TZVP/COSMO, scaling factor: 0.99, Gaussian convolution with FWHM=8 cm^{-1}.

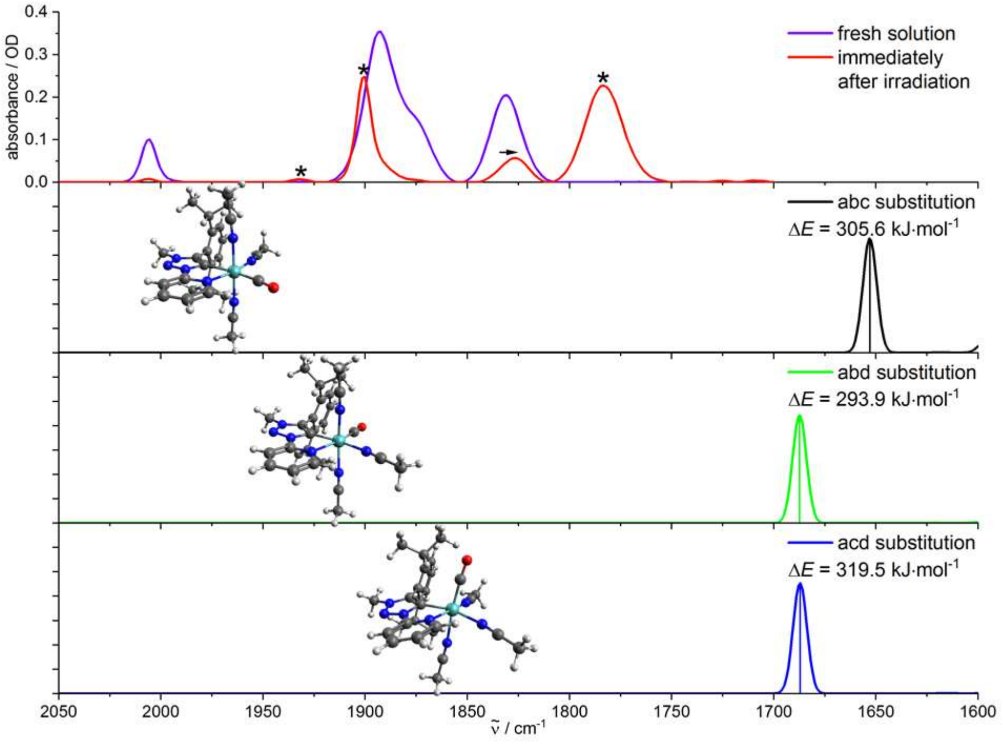

Figure S43. Experimental IR spectra of **Mo** in MeCN before and after irradiation (upper trace, bands marked with asterisks result from the formed photoproduct(s)) and calculated IR spectra of the photoproducts with a substitution of three CO ligands for three MeCN molecules (three lower traces), including the optimized structures and calculated enthalpies of reaction. A coordinated CO ligand remains in equatorial position for isomers abc/abd and in axial position for isomer acd. Calculations: DFT/B3LYP-D3(BJ)/def2-TZVP/COSMO, scaling factor: 0.99, Gaussian convolution with FWHM=8 cm^{-1}.

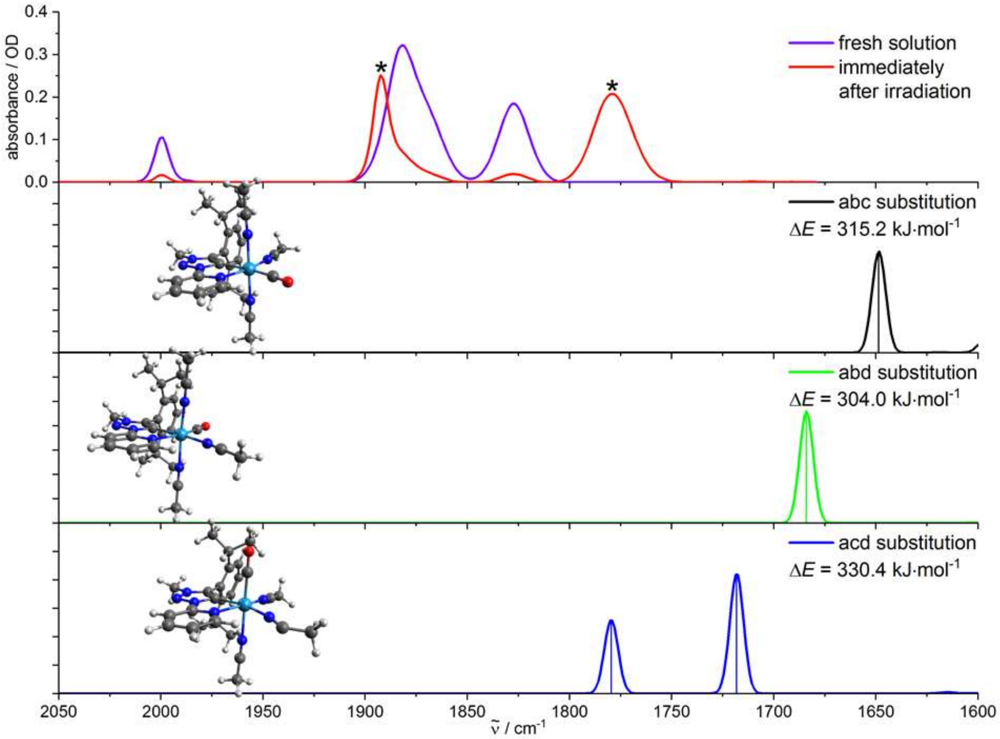

Figure S44. Experimental IR spectra of **W** in MeCN before and after irradiation (upper trace, bands marked with asterisks result from the formed photoproduct(s)) and calculated IR spectra of the photoproducts with a substitution of three CO ligands for three MeCN molecules (three lower traces), including the optimized structures and calculated enthalpies of reaction. A coordinated CO ligand remains in equatorial position for isomers abc/abd and in axial position for isomer acd. Calculations: DFT/B3LYP-D3(BJ)/def2-TZVP/COSMO, scaling factor: 0.99, Gaussian convolution with FWHM=8 cm^{-1}.

4.4.2 Trisubstitution in pyridine

Figure S45. Experimental IR spectra of **Cr** in py before and after irradiation (upper trace, bands marked

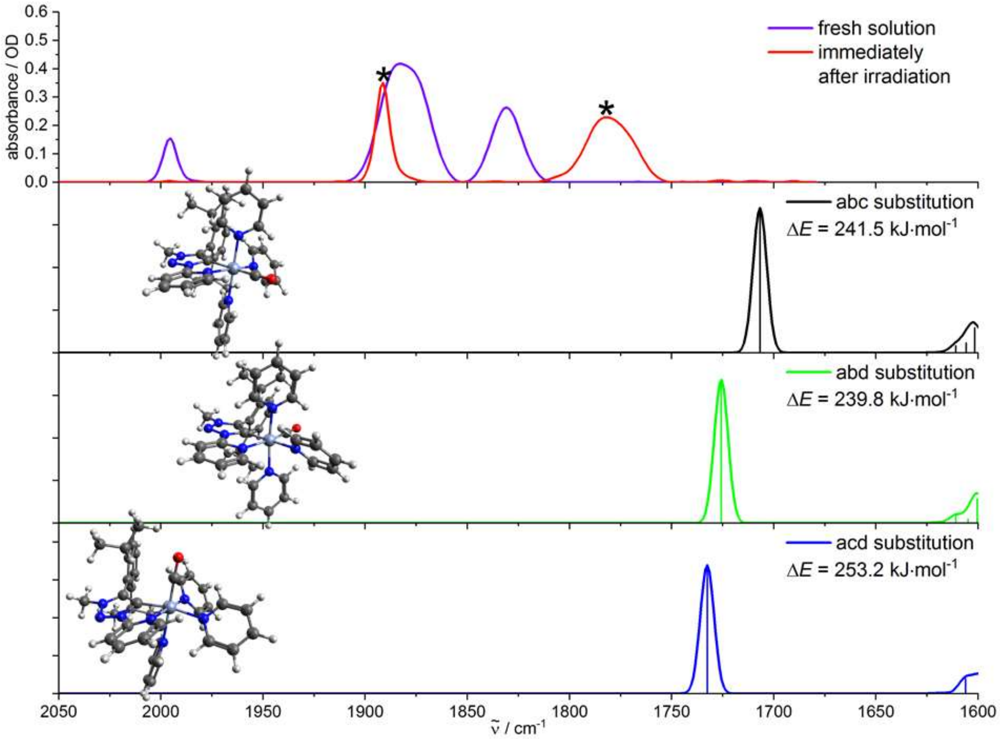

with asterisks result from the formed photoproduct(s)) and calculated IR spectra of the photoproducts with a substitution of three CO ligands for three py molecules (three lower traces), including the optimized structures and calculated enthalpies of reaction. A coordinated CO ligand remains in equatorial position for isomers abc/abd and in axial position for isomer acd. Calculations: DFT/B3LYP-D3(BJ)/def2-TZVP/COSMO, scaling factor: 0.99, Gaussian convolution with FWHM=8 cm^{-1}.

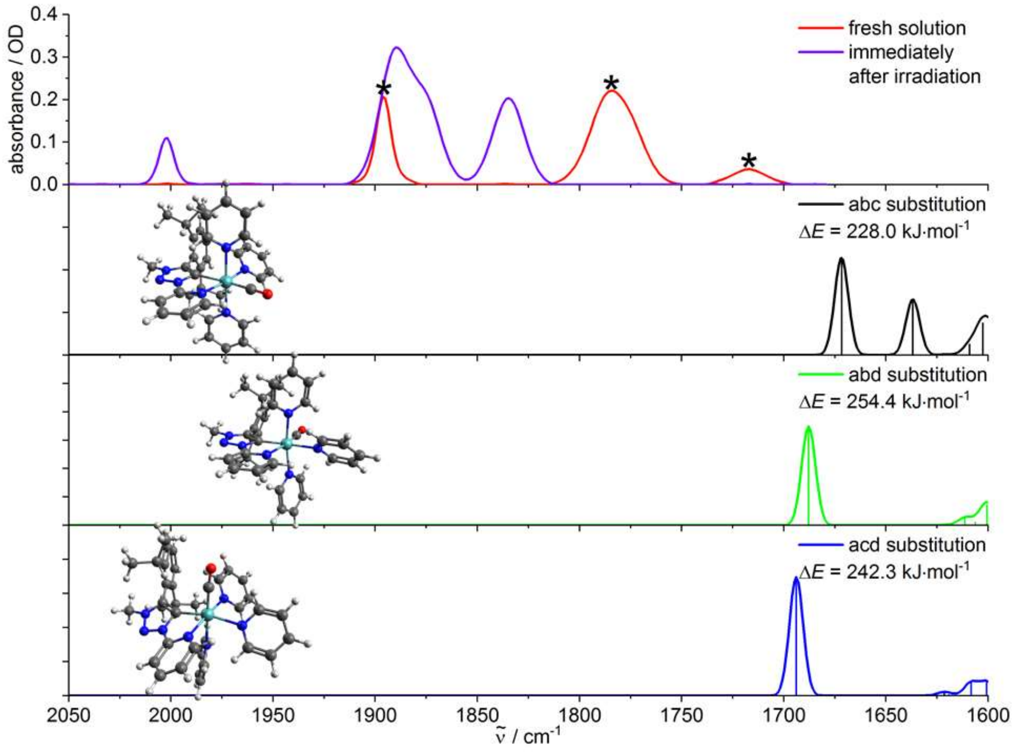

Figure S46. Experimental IR spectra of **Mo** in py before and after irradiation (upper trace, bands marked with asterisks result from the formed photoproduct(s)) and calculated IR spectra of the photoproducts with a substitution of three CO ligands for three py molecules (three lower traces), including the optimized structures and calculated enthalpies of reaction. A coordinated CO ligand remains in equatorial position for isomers abc/abd and in axial position for isomer acd. Calculations: DFT/B3LYP-D3(BJ)/def2-TZVP/COSMO, scaling factor: 0.99, Gaussian convolution with FWHM=8 cm^{-1}.

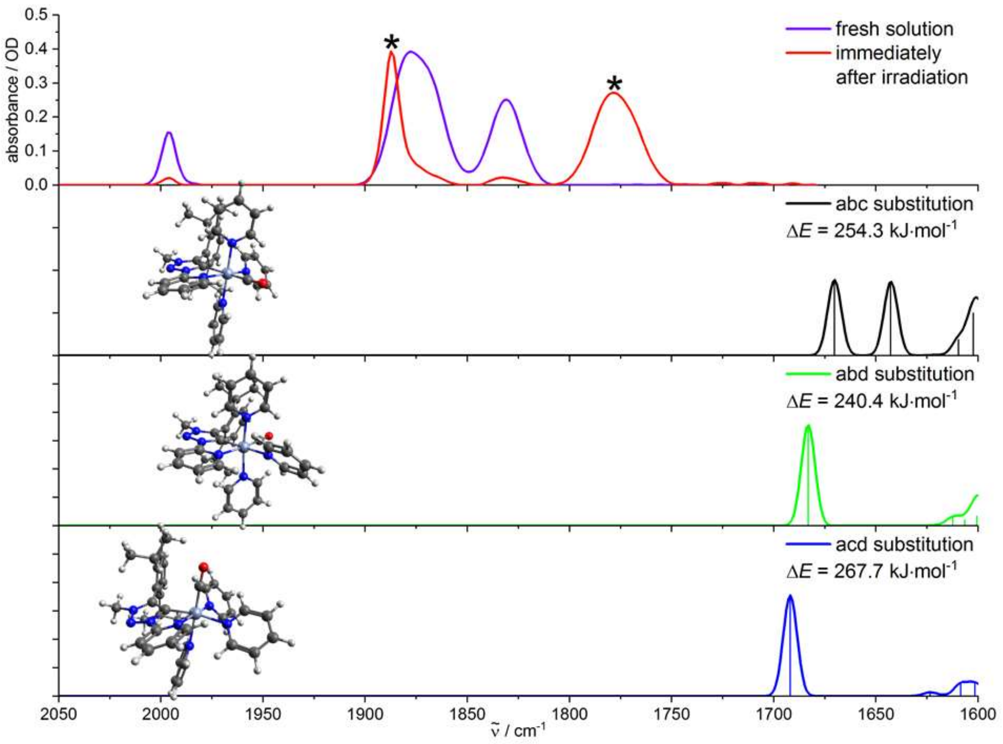

Figure S47. Experimental IR spectra of **W** in py before and after irradiation (upper trace, bands marked with asterisks result from the formed photoproduct(s)) and calculated IR spectra of the photoproducts with a substitution of three CO ligands for three py molecules (three lower traces), including the optimized structures and calculated enthalpies of reaction. A coordinated CO ligand remains in equatorial position for isomers abc/abd and in axial position for isomer acd. Calculations: DFT/B3LYP-D3(BJ)/def2-TZVP/COSMO, scaling factor: 0.99, Gaussian convolution with FWHM=8 cm^{-1}.

4.5 Formation of a metallaketene

4.5.1 Formation of a metallaketene in acetonitrile

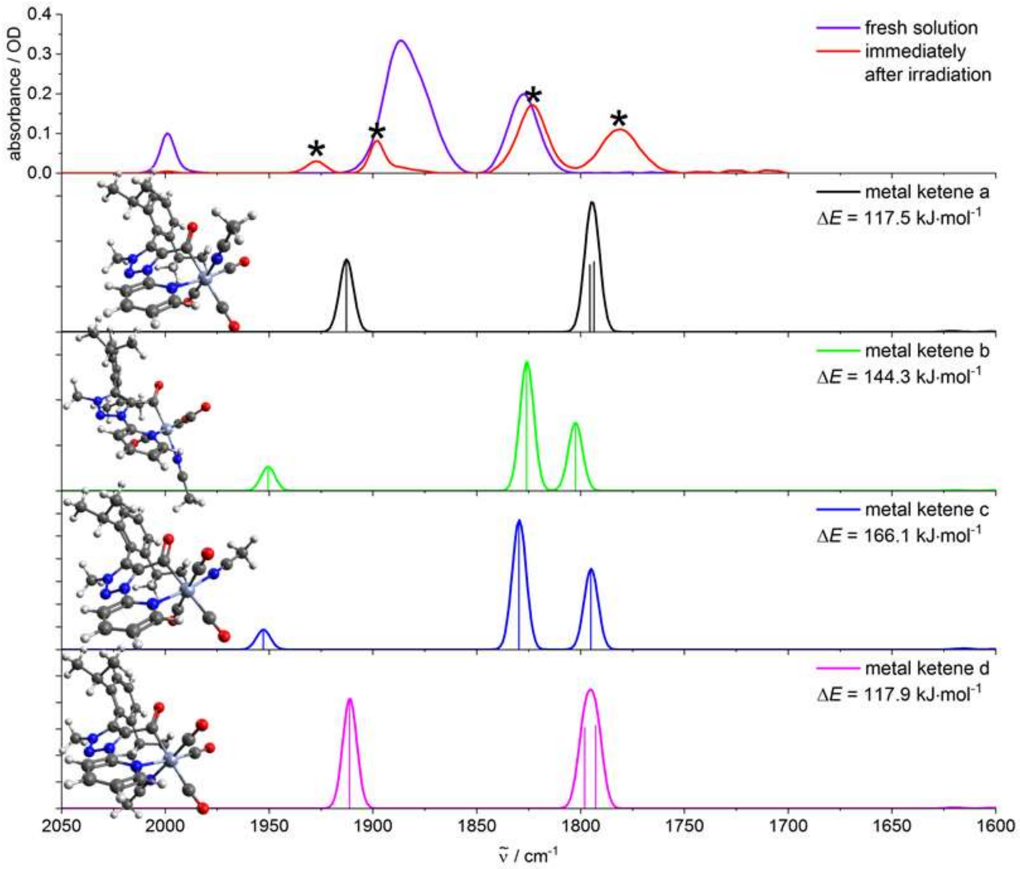

Figure S48. Experimental IR spectra of **Cr** in MeCN before and after irradiation (upper trace, bands marked with asterisks result from the formed photoproduct(s)) and calculated IR spectra of the photoproducts with formation of a metallaketene (four lower traces), including the optimized structures and calculated enthalpies of reaction. The coordinated MeCN is localized in axial position for isomers a/d and in equatorial position for b/c. Calculations: DFT/B3LYP-D3(BJ)/def2-TZVP/COSMO, scaling factor: 0.99), Gaussian convolution with FWHM=8 cm^{-1}.

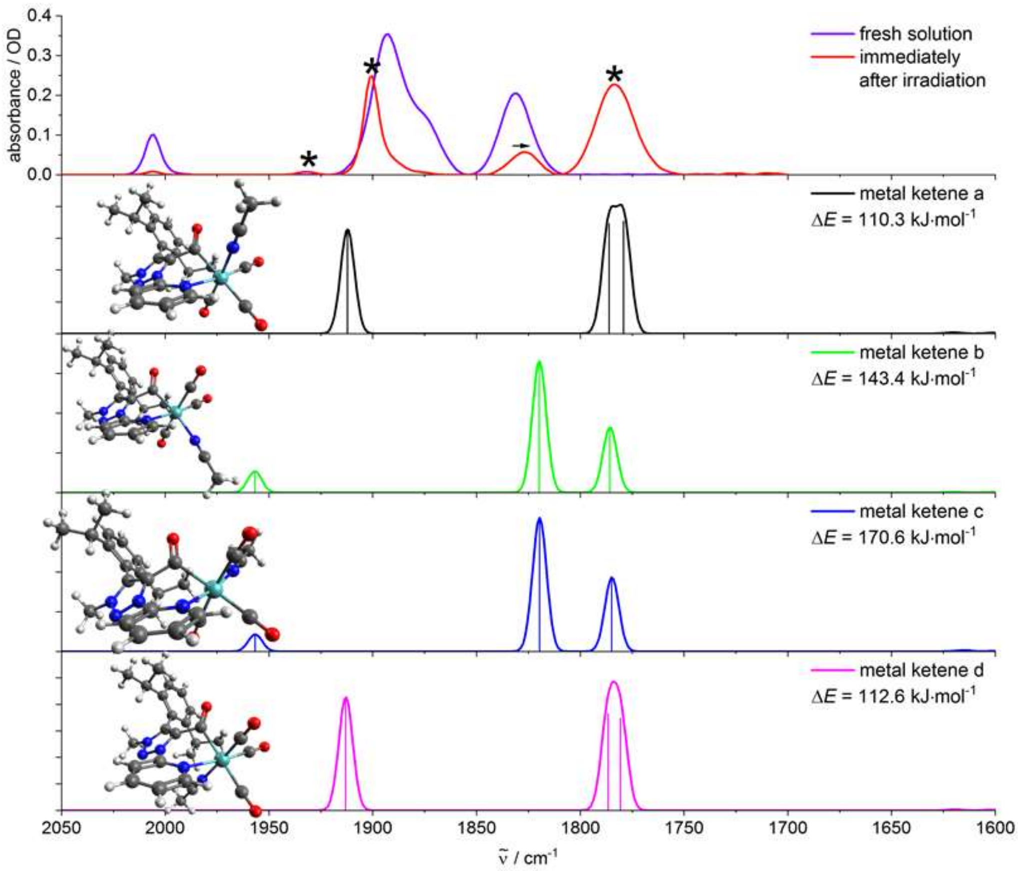

Figure S49. Experimental IR spectra of **Mo** in MeCN before and after irradiation (upper trace, bands marked with asterisks result from the formed photoproduct(s)) and calculated IR spectra of the photoproducts with formation of a metallaketene (four lower traces), including the optimized structures and calculated enthalpies of reaction. The coordinated MeCN is localized in axial position for isomers a/d and in equatorial position for b/c. Calculations: DFT/B3LYP-D3(BJ)/def2-TZVP/COSMO, scaling factor: 0.99), Gaussian convolution with FWHM=8 cm^{-1}.

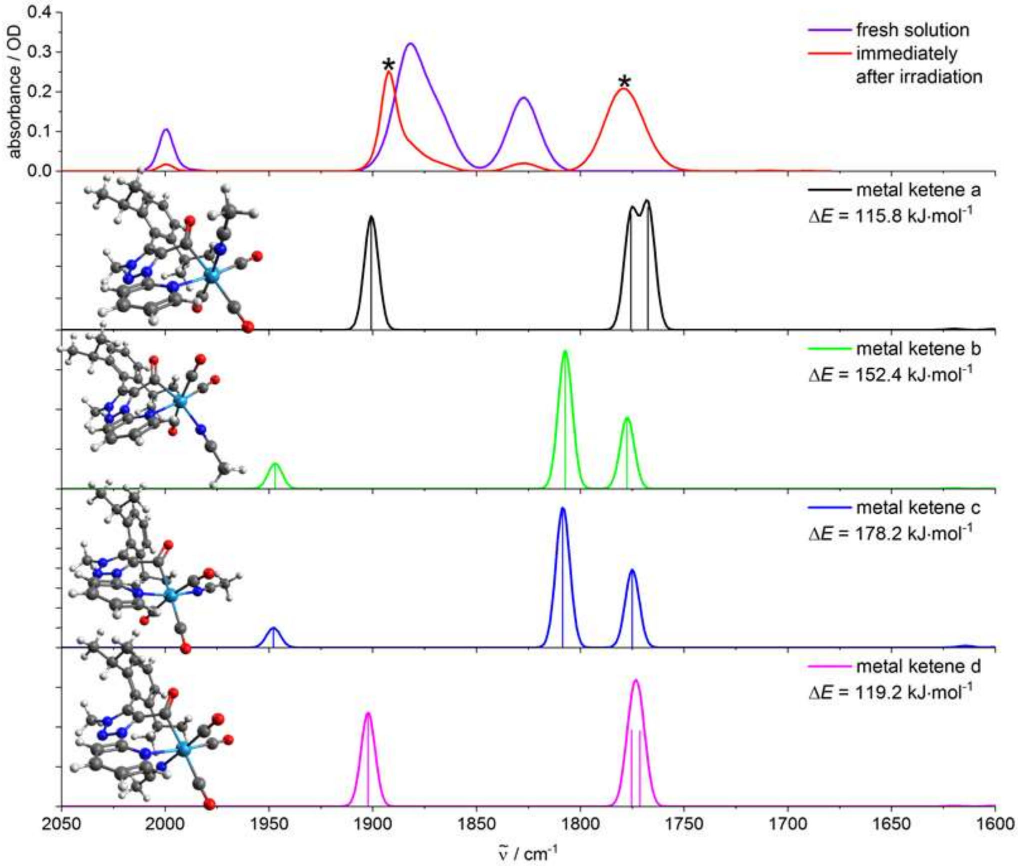

Figure S50. Experimental IR spectra of **W** in MeCN before and after irradiation (upper trace, bands marked with asterisks result from the formed photoproduct(s)) and calculated IR spectra of the photoproducts with formation of a metallaketene (four lower traces), including the optimized structures and calculated enthalpies of reaction. The coordinated MeCN is localized in axial position for isomers a/d and in equatorial position for b/c. Calculations: DFT/B3LYP-D3(BJ)/def2-TZVP/COSMO, scaling factor: 0.99, Gaussian convolution with FWHM=8 cm^{-1}.

4.5.2 Formation of a metallaketene in pyridine

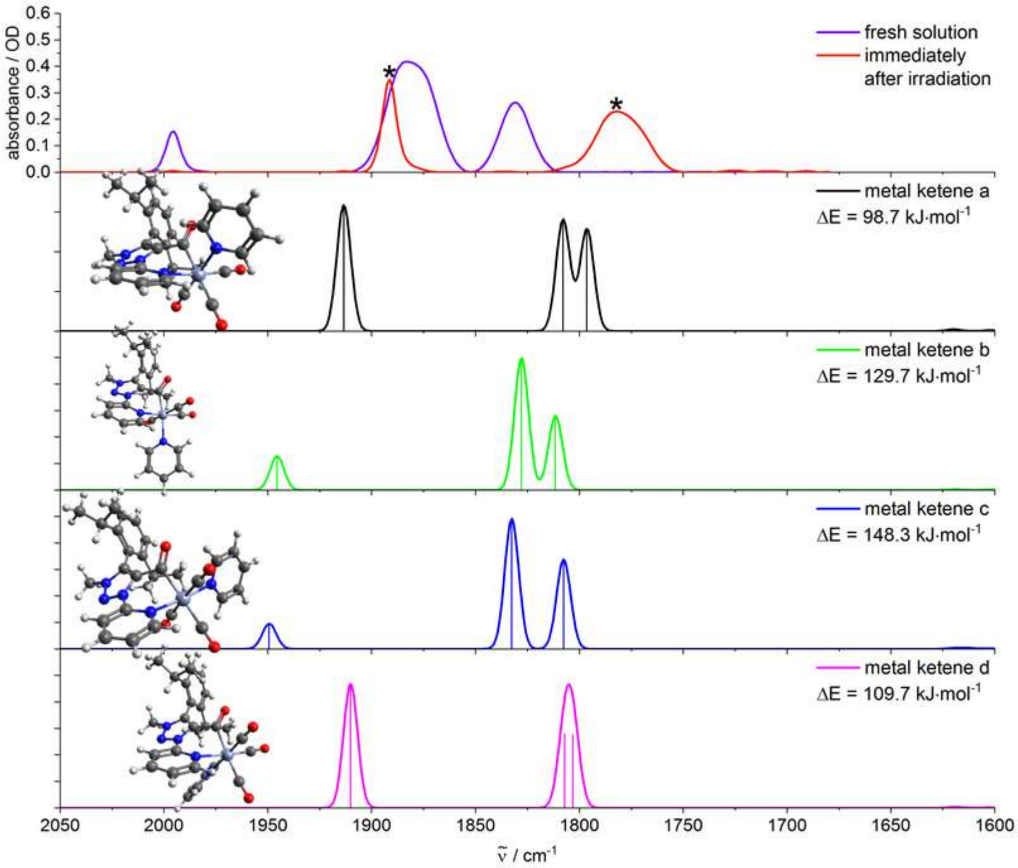

Figure S51. Experimental IR spectra of **Cr** in py before and after irradiation (upper trace, bands marked with asterisks result from the formed photoproduct(s)) and calculated IR spectra of the photoproducts with formation of a metallaketene (four lower traces), including the optimized structures and calculated enthalpies of reaction. The coordinated py is localized in axial position for isomers a/d and in equatorial position for b/c. Calculations: DFT/B3LYP-D3(BJ)/def2-TZVP/COSMO, scaling factor: 0.99, Gaussian convolution with FWHM=8 cm^{-1}.

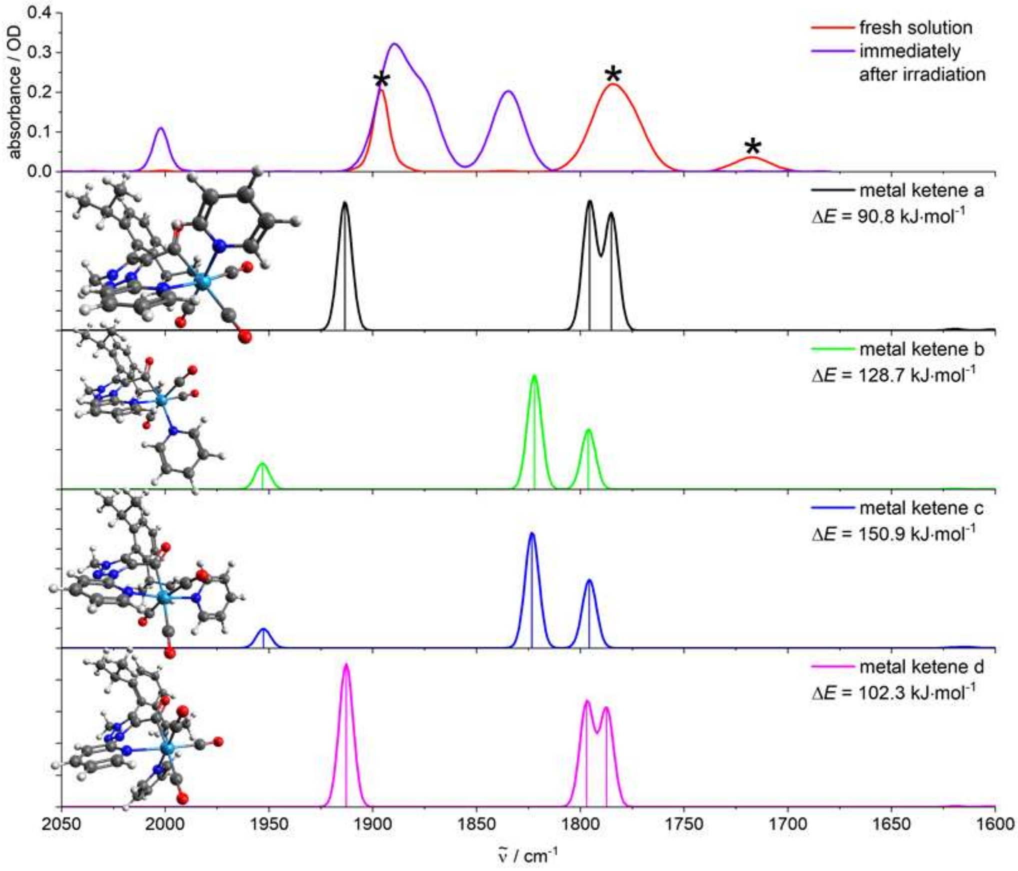

Figure S52. Experimental IR spectra of **Mo** in py before and after irradiation (upper trace, bands marked with asterisks result from the formed photoproduct(s)) and calculated IR spectra of the photoproducts with formation of a metallaketene (four lower traces), including the optimized structures and calculated enthalpies of reaction. The coordinated py is localized in axial position for isomers a/d and in equatorial position for b/c. Calculations: DFT/B3LYP-D3(BJ)/def2-TZVP/COSMO, scaling factor: 0.99, Gaussian convolution with FWHM=8 cm^{-1}.

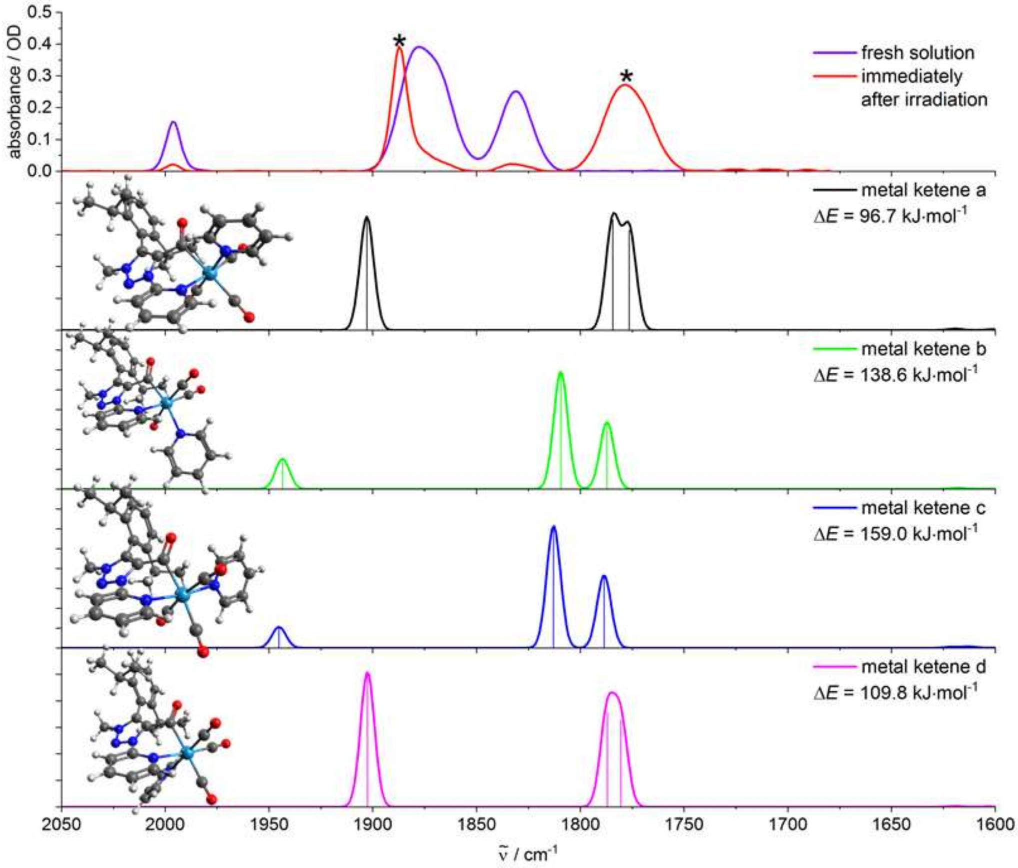

Figure S53. Experimental IR spectra of **W** in py before and after irradiation (upper trace, bands marked with asterisks result from the formed photoproduct(s)) and calculated IR spectra of the photoproducts with formation of a metallaketene (four lower traces), including the optimized structures and calculated enthalpies of reaction. The coordinated py is localized in axial position for isomers a/d and in equatorial position for b/c. Calculations: DFT/B3LYP-D3(BJ)/def2-TZVP/COSMO, scaling factor: 0.99, Gaussian convolution with FWHM=8 cm^{-1}.

4.6 Further isomerizations

4.6.1 Further isomerizations in acetonitrile

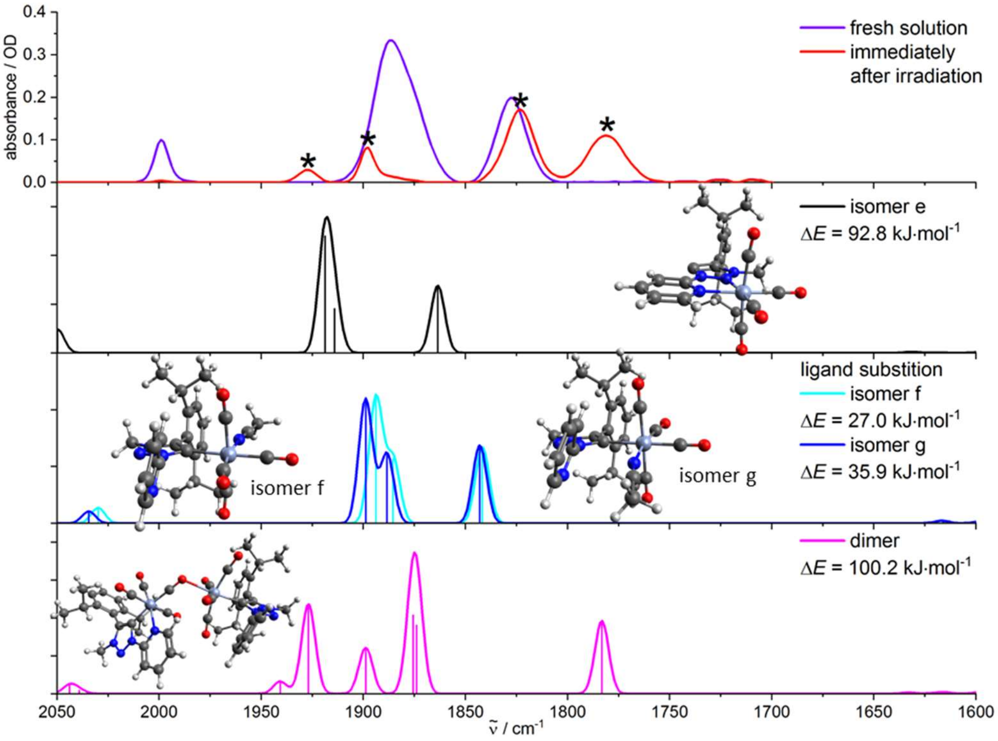

Figure S54. Experimental IR spectra of **Cr** in MeCN before and after irradiation (upper trace, bands marked with asterisks result from the formed photoproduct(s)) and calculated IR spectra of isomers e, f and g as well as a dimer structure (three lower traces), including the optimized structures and calculated enthalpies of reaction. Calculations: DFT/B3LYP-D3(BJ)/def2-TZVP/COSMO, scaling factor: 0.99, Gaussian convolution with FWHM=8 cm^{-1}.

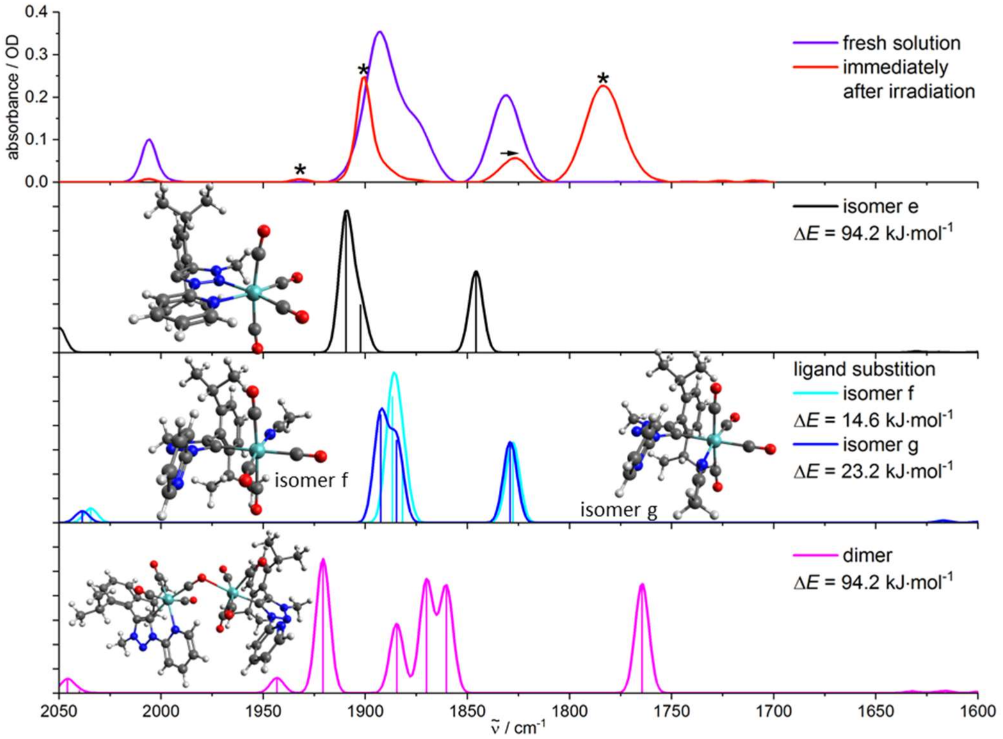

Figure S55. Experimental IR spectra of **Mo** in MeCN before and after irradiation (upper trace, bands marked with asterisks result from the formed photoproduct(s)) and calculated IR spectra of isomers e, f and g as well as a dimer structure (three lower traces), including the optimized structures and calculated enthalpies of reaction. Calculations: DFT/B3LYP-D3(BJ)/def2-TZVP/COSMO, scaling factor: 0.99, Gaussian convolution with FWHM=8 cm^{-1}.

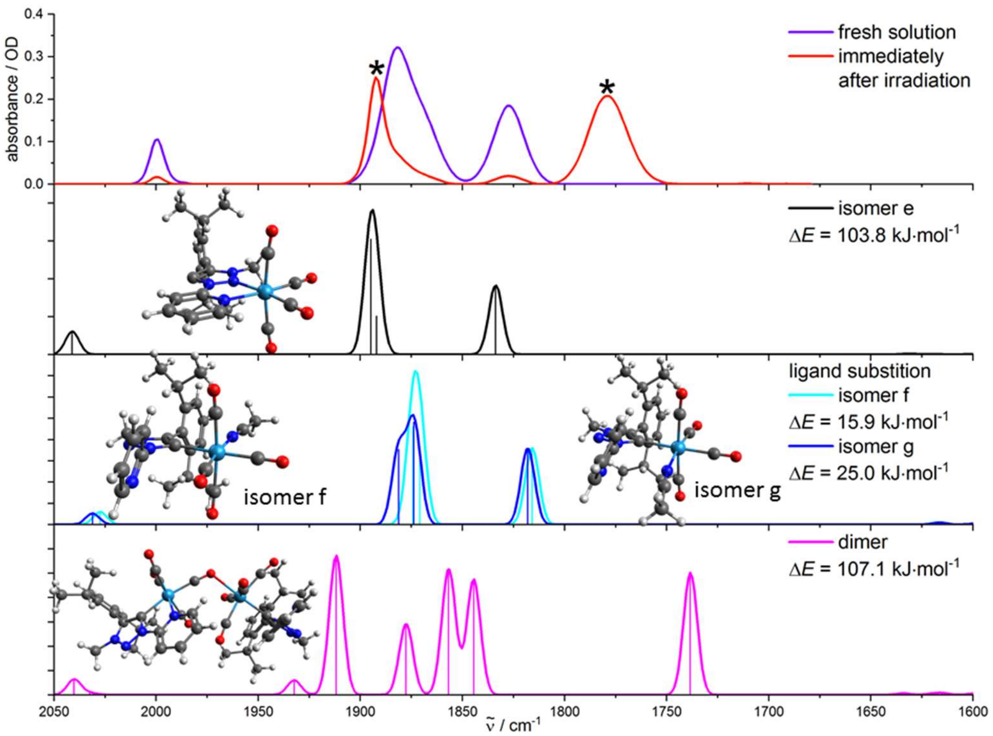

Figure S56. Experimental IR spectra of **W** in MeCN before and after irradiation (upper trace, bands marked with asterisks result from the formed photoproduct(s)) and calculated IR spectra of isomers e, f and g as well as a dimer structure (three lower traces), including the optimized structures and calculated enthalpies of reaction. Calculations: DFT/B3LYP-D3(BJ)/def2-TZVP/COSMO, scaling factor: 0.99, Gaussian convolution with FWHM=8 cm^{-1}.

4.6.2 Further isomerizations in pyridine

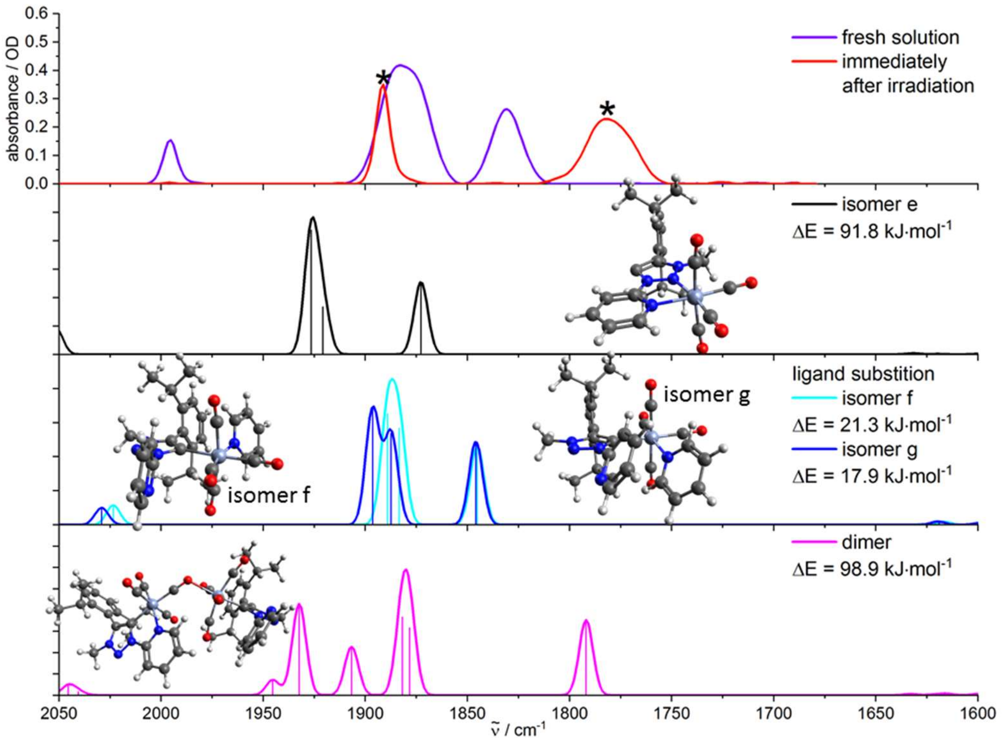

Figure S57. Experimental IR spectra of **Cr** in py before and after irradiation (upper trace, bands marked with asterisks result from the formed photoproduct(s)) and calculated IR spectra of isomers e, f and g as well as a dimer structure (three lower traces), including the optimized structures and calculated enthalpies of reaction. Calculations: DFT/B3LYP-D3(BJ)/def2-TZVP/COSMO, scaling factor: 0.99, Gaussian convolution with FWHM=8 cm^{-1}.

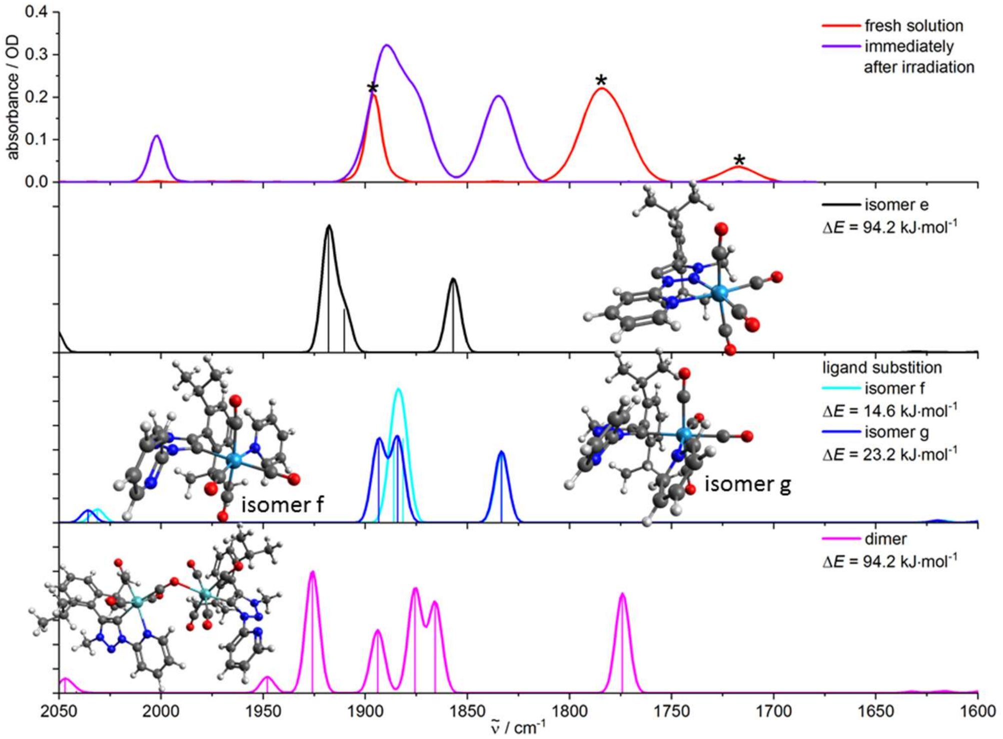

Figure S58. Experimental IR spectra of **Mo** in py before and after irradiation (upper trace, bands marked with asterisks result from the formed photoproduct(s)) and calculated IR spectra of isomers e, f and g as well as a dimer structure (three lower traces), including the optimized structures and calculated enthalpies of reaction. Calculations: DFT/B3LYP-D3(BJ)/def2-TZVP/COSMO, scaling factor: 0.99, Gaussian convolution with FWHM=8 cm^{-1}.

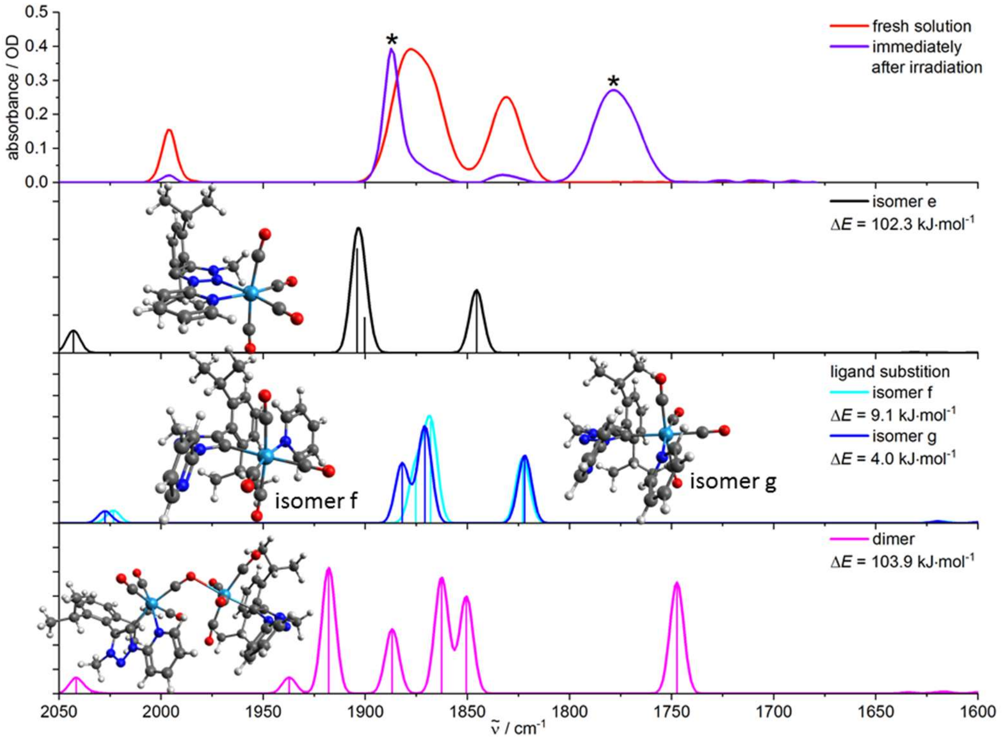

Figure S59. Experimental IR spectra of **W** in py before and after irradiation (upper trace, bands marked with asterisks result from the formed photoproduct(s)) and calculated IR spectra of isomers e, f and g as well as a dimer structure (three lower traces), including the optimized structures and calculated enthalpies of reaction. Calculations: DFT/B3LYP-D3(BJ)/def2-TZVP/COSMO, scaling factor: 0.99, Gaussian convolution with FWHM=8 cm^{-1}.

4.7 Loss of a CO ligand in a KBr matrix

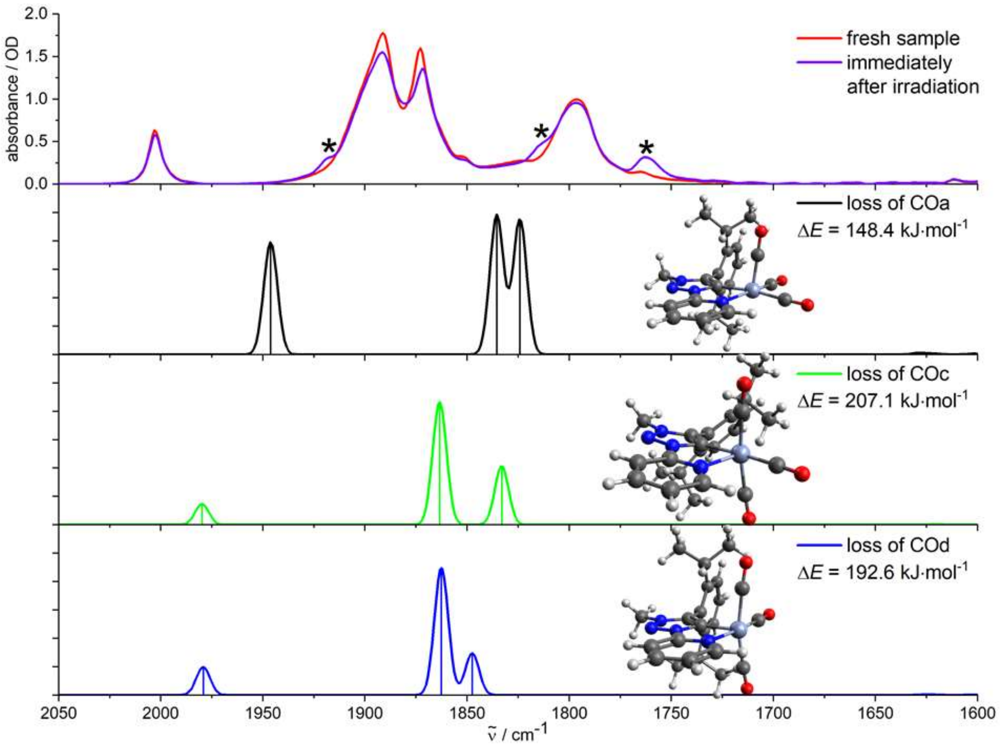

Figure S60. Irradiation (λ_{ex} = 355 nm) of fresh samples of **Cr** (KBr pellet) at 10 K and calculated IR spectra of different photoproducts with loss of a CO ligand, including the optimized structures and calculated enthalpies of reaction. The vacant coordination site is localized in axial position for isomer a and in equatorial position for structures c and d, respectively. Calculations: DFT/B3LYP-D3(BJ)/def2-TZVP/COSMO, scaling factor: 0.99, Gaussian convolution with FWHM=8 cm^{-1}.

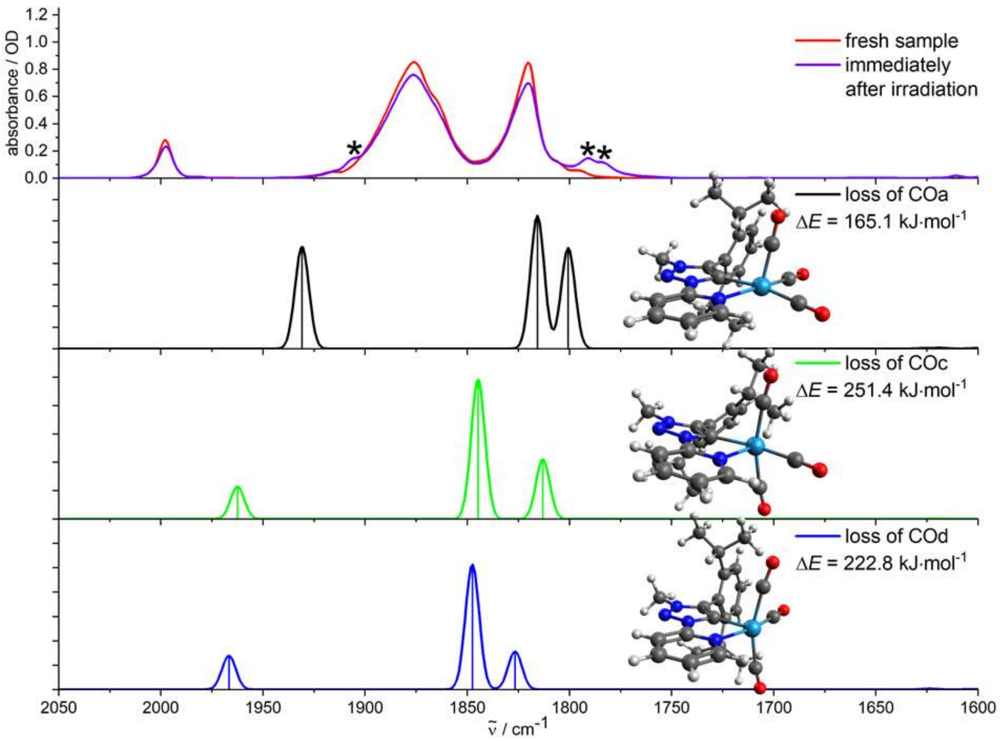

Figure S61. Irradiation (λ_{ex} = 355 nm) of fresh samples of **W** (KBr pellet) at 10 K and calculated IR spectra of different photoproducts with loss of a CO ligand, including the optimized structures and calculated enthalpies of reaction. The vacant coordination site is localized in axial position for isomer a and in equatorial position for structures c and d, respectively. Calculations: DFT/B3LYP-D3(BJ)/def2-TZVP/COSMO, scaling factor: 0.99, Gaussian convolution with FWHM=8 cm^{-1}.

4.8 Photochemistry in frozen valeronitrile

Figure S62. Difference spectra (irradiated – fresh) of **Cr** recorded in frozen and liquid solution at 20 K

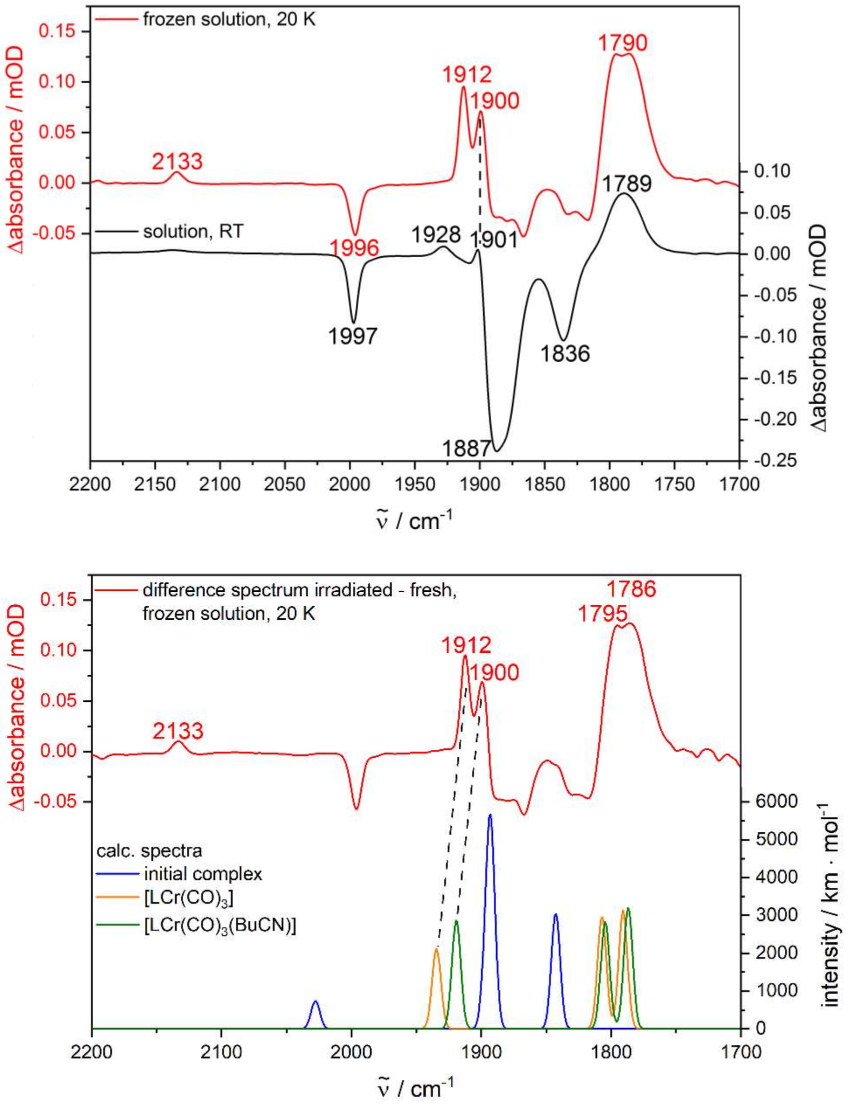

and at room temperature, respectively (top). Comparison of the difference spectrum at 20 K with calculated IR spectra of the initial complex as well as the photoproducts [LCr(CO)$_3$] (vacant coordination site in axial position) and [LCr(CO)$_3$BuCN$_{ax}$] (bottom). The dashed lines are guides to the eye. Calculations: DFT/B3LYP-D3(BJ)/def2-TZVP/COSMO, scaling factor: 0.99, Gaussian convolution with FWHM=8 cm^{-1}.

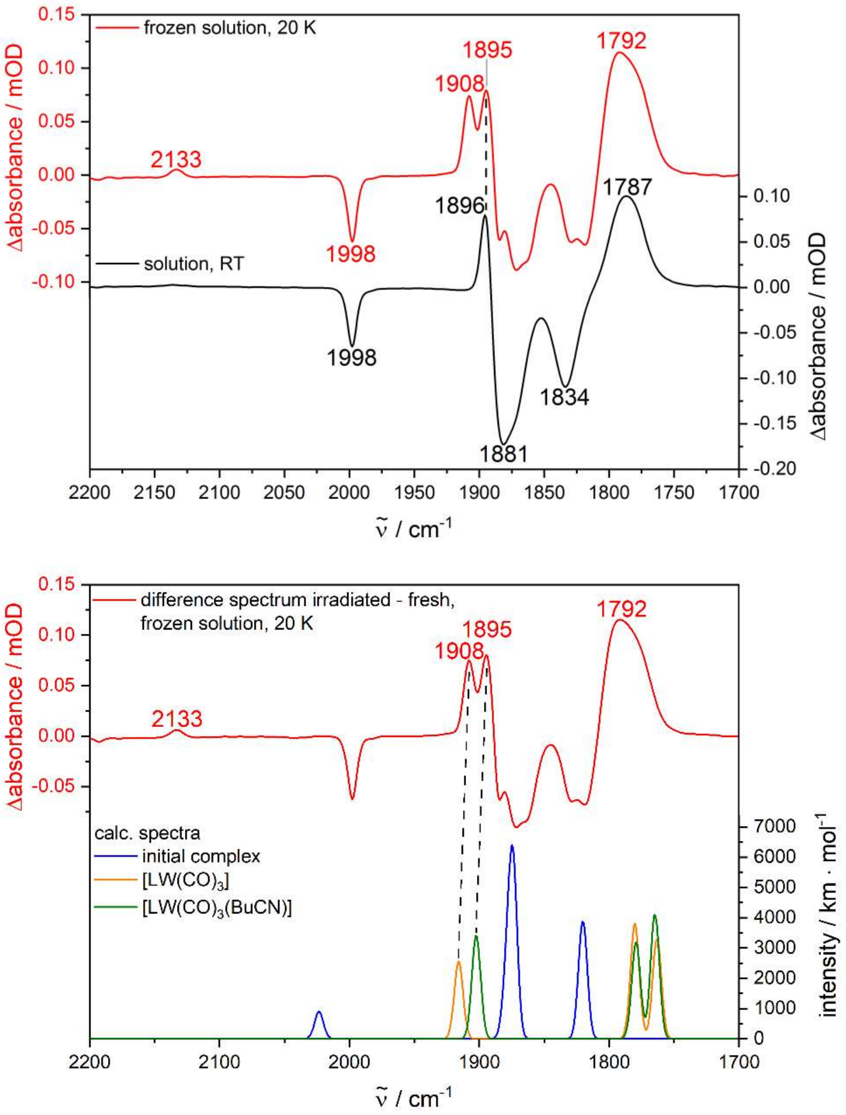

Figure S63. Difference spectra (irradiated – fresh) of **W** recorded in frozen and liquid solution at 20 K and at room temperature, respectively (top). Comparison of the difference spectrum at 20 K with calculated IR spectra of the initial complex as well as the photoproducts [LW(CO)$_3$] (vacant coordination site in axial position) and [LW(CO)$_3$BuCN$_{ax}$] (bottom). The dashed lines are guides to the eye. Calculations: DFT/B3LYP-D3(BJ)/def2-TZVP/COSMO, scaling factor: 0.99, Gaussian convolution with FWHM=8 cm^{-1}.

5 Comparison between experimental and theoretical UV/VIS spectra

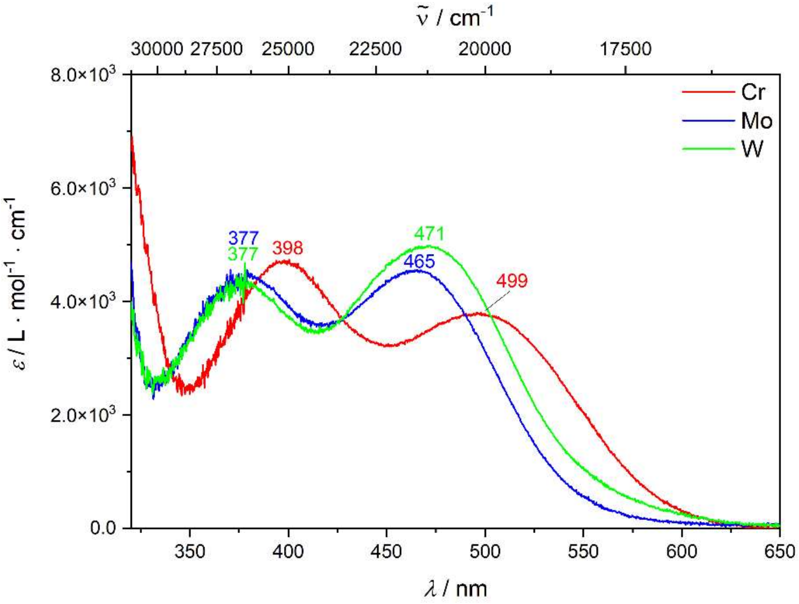

Figure S64. Experimental UV/VIS spectra (extinction coefficients) of fresh solutions of **Cr**, **Mo** and **W** in py.

Table S1. Extinction coefficients of **Cr**, **Mo** and **W** in MeCN as well as py at the wavelengths of 532, 355 and 266 nm.

**Complex**	**solvent**	**λ / nm**	**ε / L · mol^{-1} · cm^{-1}**
**Cr**	MeCN*	532	1741
		355	2746
		266	25730
	py	532	2910
		355	2634
**Mo**	MeCN*	532	621
		355	3897
		266	31797
	py	532	1150
		355	3795
**W**	MeCN*	532	987
		355	3247
		266	24486
	py	532	1775
		355	3630

(*): values taken from ref. [5]

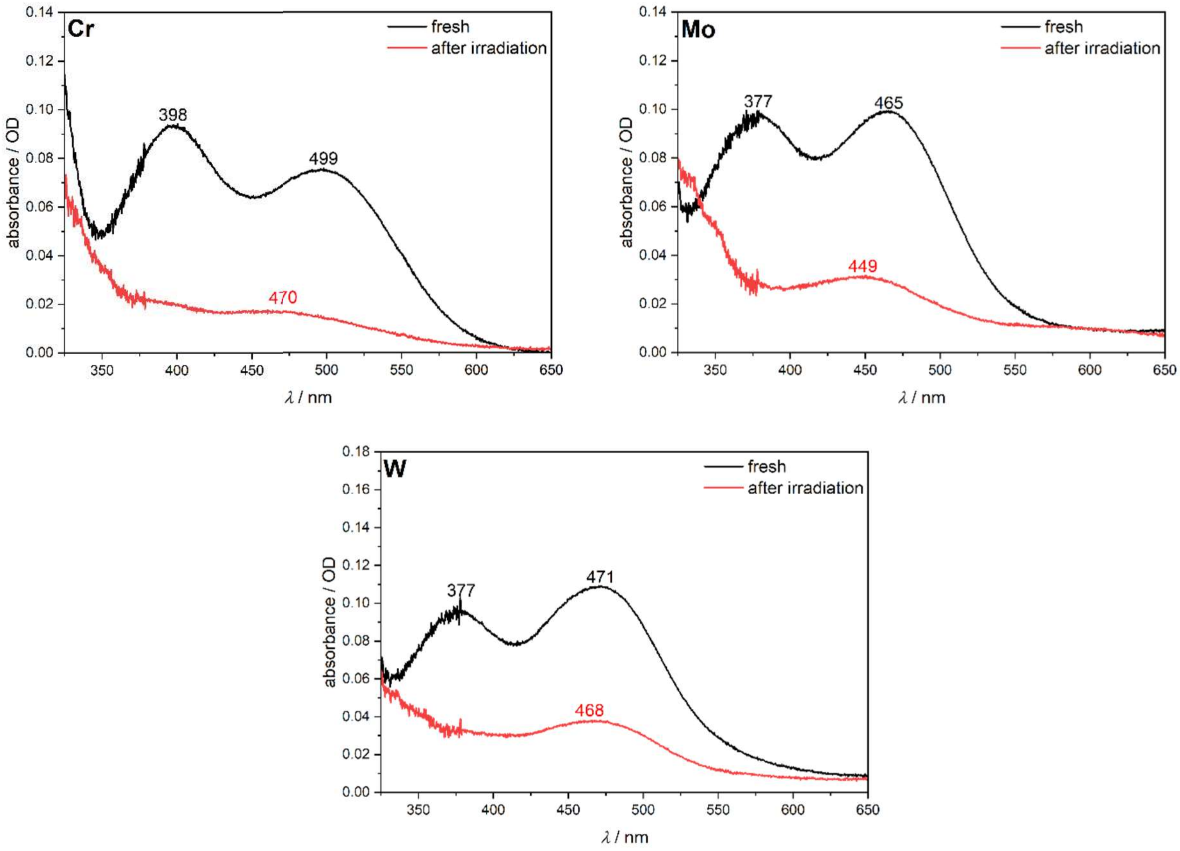

Figure S65. Experimental UV/VIS absorption spectra of **Cr** (top, left), **Mo** (top, right) and **W** (bottom) recorded of fresh solutions and immediately after irradiation at λ_{ex} = 355 nm.

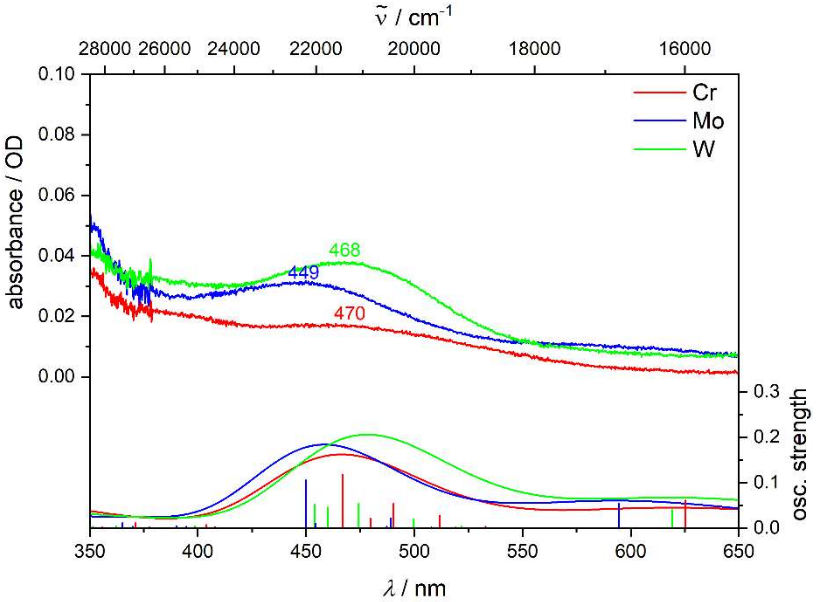

Figure S66. Experimental UV/VIS spectra of solutions of **Cr**, **Mo** and **W** in py after irradiation at 355 nm (upper trace) as well as calculated UV/VIS absorption frequencies (sticks) and convoluted spectra (curves) of the conceivable photoproducts with substitution of an axial CO ligand for a py molecule

(lower trace). Calculations: TDDFT/B3LYP-D3(BJ)/def2-TZVP/COSMO, convolution with FWHM = 1500 cm^{-1}.

Table S2. Assignment of the calculated lowest electronic excitations in the singlet manifold for the photoproduct with an axial CO ligand substituted by a py molecule for Cr in py (TDDFT/B3LYP D3(BJ)/def2-TZVP/COSMO: py).

**transition**	**$\tilde{\nu}$ / cm^{-1}**	**λ / nm**	**%**	**donor orbital**	**acceptor orbital**	**assignment**
7	21082	474	42.7	HOMO	LUMO+2	d_{Cr}, $\pi_{Cr\text{-}CO}$ → $\pi_{pyridine}$
			40.1	HOMO-2	LUMO+1	d_{Cr}, $\pi_{Cr\text{-}CO}$ → $\pi_{ligand\text{-}pyr}$
			11.2	HOMO-1	LUMO+2	d_{Cr}, $\pi_{Cr\text{-}CO}$ → $\pi_{ligand\text{-}pyr}$
8	21473	460	37.0	HOMO-2	LUMO+2	d_{Cr}, $\pi_{Cr\text{-}CO}$ → $\pi_{pyridine}$
			36.5	HOMO-2	LUMO+1	d_{Cr}, $\pi_{Cr\text{-}CO}$ → $\pi_{ligand\text{-}pyr}$
			12.0	HOMO	LUMO+2	d_{Cr}, $\pi_{Cr\text{-}CO}$ → $\pi_{pyridine}$
			7.8	HOMO-1	LUMO+1	d_{Cr}, $\pi_{Cr\text{-}CO}$ → $\pi_{ligand\text{-}pyr}$
9	22029	454	57.9	HOMO-2	LUMO+2	d_{Cr}, $\pi_{Cr\text{-}CO}$ → $\pi_{pyridine}$
			19.4	HOMO	LUMO+2	d_{Cr}, $\pi_{Cr\text{-}CO}$ → $\pi_{pyridine}$
			10.6	HOMO-2	LUMO+1	d_{Cr}, $\pi_{Cr\text{-}CO}$ → $\pi_{ligand\text{-}pyr}$
			6.1	HOMO	LUMO+2	d_{Cr}, $\pi_{Cr\text{-}CO}$ → $\pi_{pyridine}$

Table S3. Assignment of the calculated lowest electronic excitations in the singlet manifold for the photoproduct with an axial CO ligand substituted by a py molecule for Mo in py (TDDFT/B3LYP D3(BJ)/def2-TZVP/COSMO: py).

transition	$\tilde{\nu}$ / cm^{-1}	λ / nm	%	donor orbital	acceptor orbital	assignment
7	21421	467	39.4	HOMO-2	LUMO+1	d_{Mo}, $\pi_{Mo\text{-}CO} \rightarrow \pi_{ligand\text{-}pyr}$
			36.0	HOMO	LUMO+2	d_{Mo}, $\pi_{Mo\text{-}CO} \rightarrow \pi_{pyridine}$
			16.0	HOMO-1	LUMO+2	d_{Mo}, $\pi_{Mo\text{-}CO} \rightarrow \pi_{pyridine}$
9	22233	450	39.3	HOMO-2	LUMO+1	d_{Mo}, $\pi_{Mo\text{-}CO} \rightarrow \pi_{ligand\text{-}pyr}$
			30.5	HOMO	LUMO+2	d_{Mo}, $\pi_{Mo\text{-}CO} \rightarrow \pi_{pyridine}$
			12.2	HOMO-1	LUMO+2	d_{Mo}, $\pi_{Mo\text{-}CO} \rightarrow \pi_{pyridine}$
			9.5	HOMO-1	LUMO+1	d_{Mo}, $\pi_{Mo\text{-}CO} \rightarrow \pi_{ligand\text{-}pyr}$

Table S4. Assignment of the calculated lowest electronic excitations in the singlet manifold for the photoproduct with an axial CO ligand substituted by a py molecule for W in py (TDDFT/B3LYP D3(BJ)/def2-TZVP/COSMO: py).

transition	$\tilde{\nu}$ / cm^{-1}	λ / nm	%	donor orbital	acceptor orbital	assignment
7	20390	490	41.5	HOMO	LUMO+2	d_{W}, $\pi_{W\text{-}CO} \rightarrow \pi_{pyridine}$
			30.0	HOMO-2	LUMO+1	d_{W}, $\pi_{W\text{-}CO} \rightarrow \pi_{ligand\text{-}pyr}$
			18.3	HOMO-2	LUMO+2	d_{W}, $\pi_{W\text{-}CO} \rightarrow \pi_{pyridine}$
			8.9	HOMO-1	LUMO+2	d_{W}, $\pi_{W\text{-}CO} \rightarrow \pi_{pyridine}$
9	21419	467	49.3	HOMO-2	LUMO+1	d_{W}, $\pi_{W\text{-}CO} \rightarrow \pi_{ligand\text{-}pyr}$
			26.4	HOMO	LUMO+2	d_{W}, $\pi_{W\text{-}CO} \rightarrow \pi_{pyridine}$
			11.7	HOMO-1	LUMO+1	d_{W}, $\pi_{W\text{-}CO} \rightarrow \pi_{ligand\text{-}pyr}$
			5.8	HOMO-1	LUMO+2	d_{W}, $\pi_{W\text{-}CO} \rightarrow \pi_{pyridine}$

6 Kinetic data for the dark reverse reaction

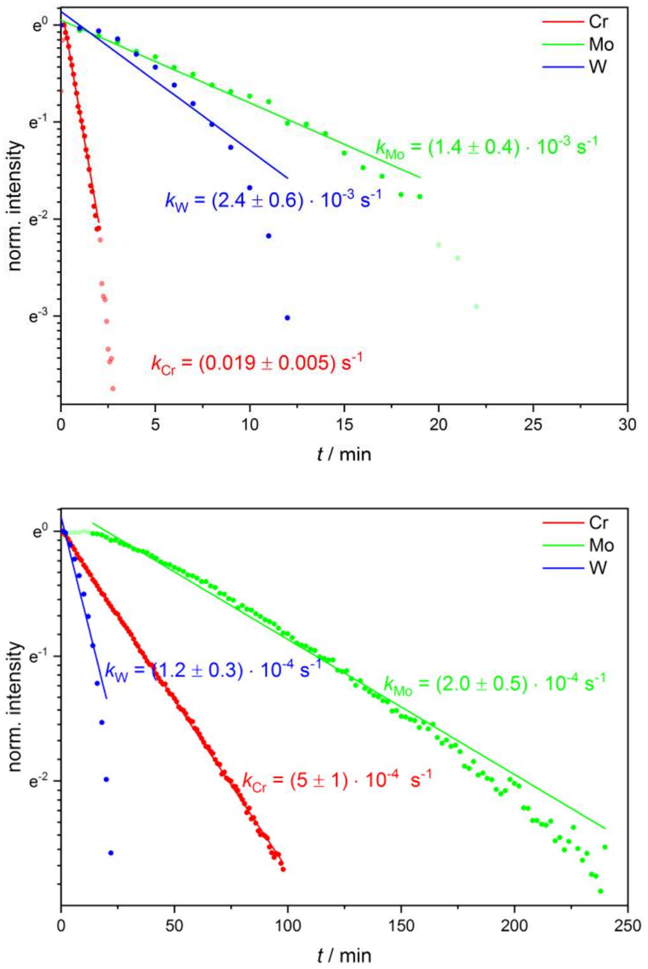

Figure S67. First order kinetic plots and fits of the dark reverse reaction of **Cr**, **Mo** and **W** measured in MeCN (top) and py (bottom) (c = 3 mM). The shown kinetics were recorded subsequent to irradiation at 532 nm. The IR intensity at the end of the irradiation period (t = 0) was normalized to 1.

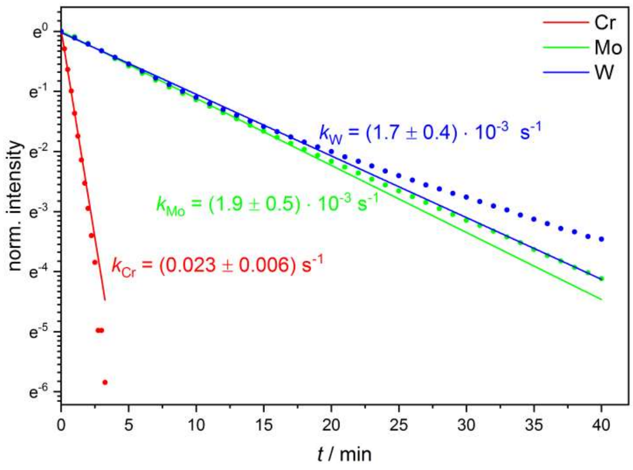

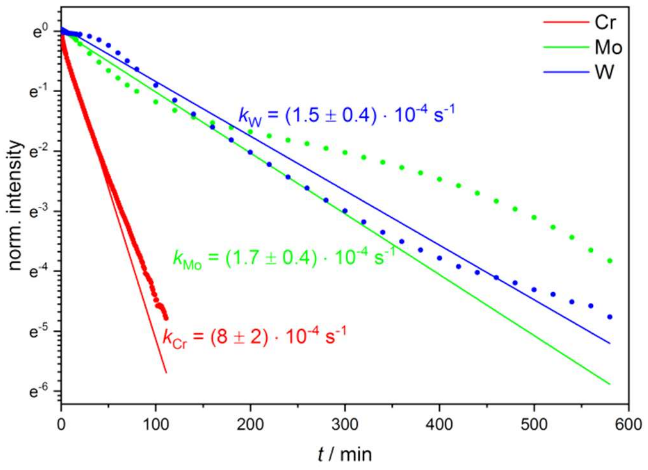

Figure S68. First order kinetic plots and fits of the dark reverse reaction of **Cr**, **Mo** and **W** measured in MeCN (top) and py (bottom) (c = 6 mM). The shown kinetics were recorded subsequent to irradiation at 355 nm. The IR intensity at the end of the irradiation period (t = 0) was normalized to 1.

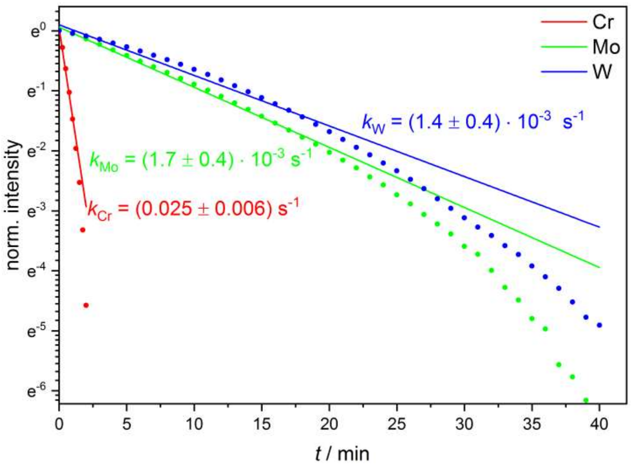

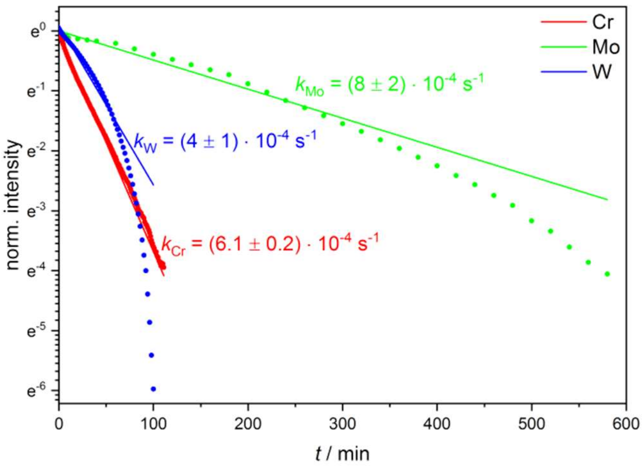

Figure S69. First order kinetic plots and fits of the dark reverse reaction of **Cr**, **Mo** and **W** measured in MeCN (top) and py (bottom) (c = 3 mM). The shown kinetics were recorded subsequent to irradiation at 355 nm. The IR intensity at the end of the irradiation period (t = 0) was normalized to 1.

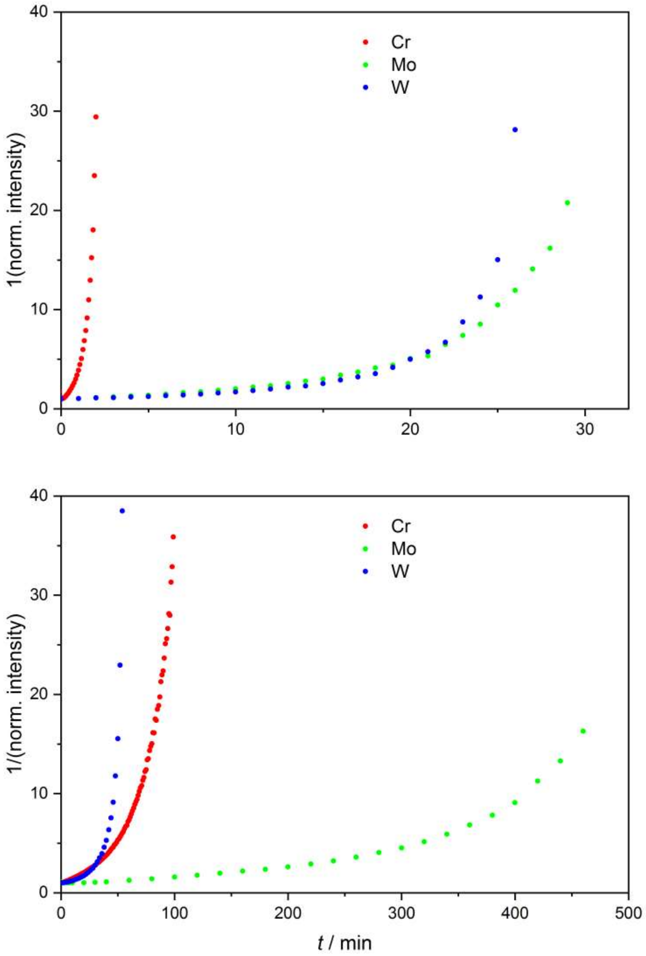

Figure S70. Second order kinetic plot of the dark reverse reaction of **Cr**, **Mo** and **W** measured in MeCN (top) and py (bottom) (c = 6 mM). The shown kinetics were recorded subsequent to irradiation at 532 nm. The IR intensity at the end of the irradiation period (t = 0) was normalized to 1.

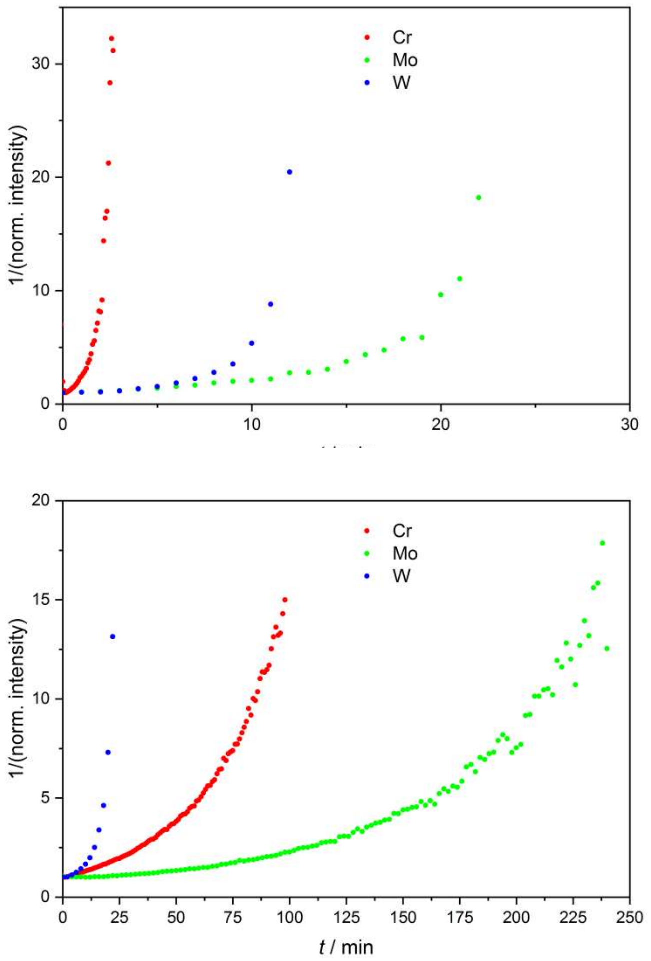

Figure S71. Second order kinetic plot of the dark reverse reaction of **Cr**, **Mo** and **W** measured in MeCN (top) and py (bottom) (c = 3 mM). The shown kinetics were recorded subsequent to irradiation at 532 nm. The IR intensity at the end of the irradiation period (t = 0) was normalized to 1.

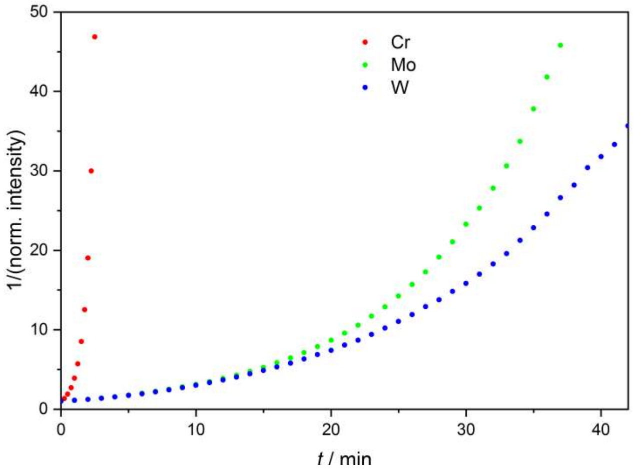

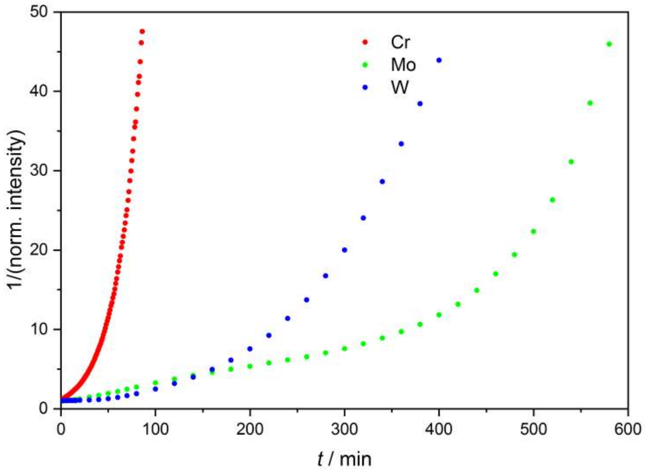

Figure S72. Second order kinetic plot of the dark reverse reaction of **Cr**, **Mo** and **W** measured in MeCN (top) and py (bottom) (c = 6 mM). The shown kinetics were recorded subsequent to irradiation at 355 nm. The IR intensity at the end of the irradiation period (t = 0) was normalized to 1.

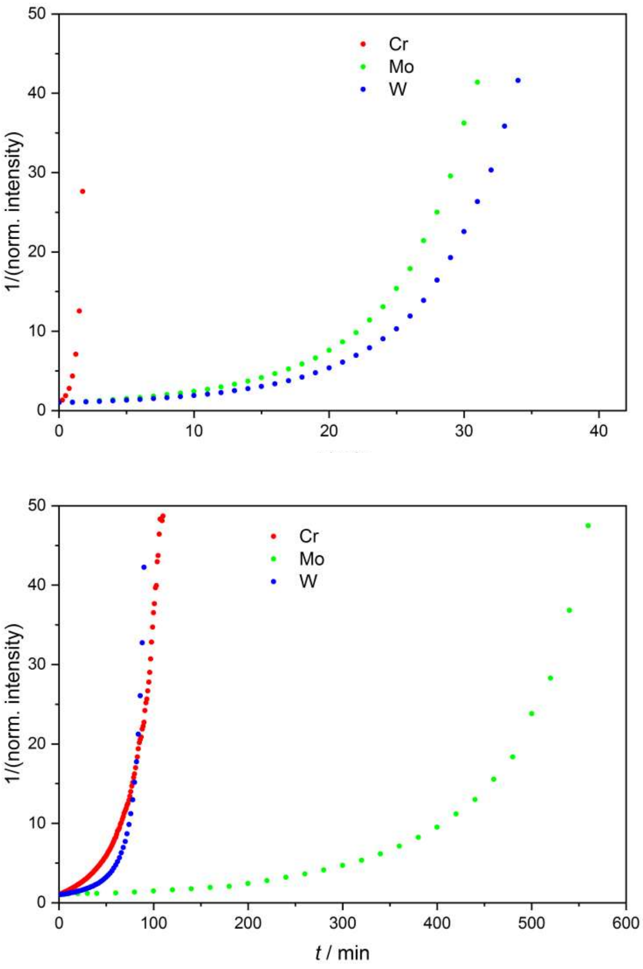

Figure S73. Second order kinetic plot of the dark reverse reaction of **Cr**, **Mo** and **W** measured in MeCN (top) and py (bottom) (c = 3 mM). The shown kinetics were recorded subsequent to irradiation at 355 nm. The IR intensity at the end of the irradiation period (t = 0) was normalized to 1.

7 Calculated bond lengths

Table S5. Calculated M−CO and C−O bond lengths in Å for **Cr**, **Mo** and **W** (DFT/B3LYP D3(BJ)/def2-TZVP/COSMO).

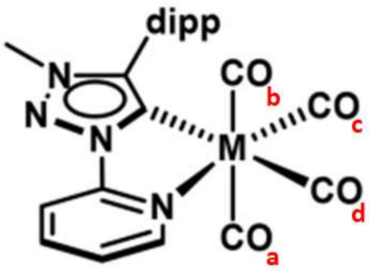

complex	medium	M-COa	COa	M-COb	COb	M-COc	COc	M-COd	COd
**Cr**	KBr[5]	1.894	1.151	1.894	1.151	1.839	1.159	1.853	1.160
	MeCN	1.893	1.152	1.892	1.152	1.835	1.161	1.848	1.163
	py	1.893	1.151	1.892	1.151	1.836	1.160	1.849	1.162
	BuCN	1.893	1.151	1.892	1.152	1.836	1.160	1.848	1.162
**Mo**	KBr[5]	2.043	1.151	2.044	1.151	1.966	1.161	1.997	1.160
	MeCN	2.042	1.151	2.043	1.151	1.962	1.163	1.992	1.163
	py	2.042	1.151	2.043	1.151	1.964	1.162	1.994	1.162
	BuCN	2.042	1.151	2.043	1.151	1.963	1.163	1.993	1.162
**W**	KBr[5]	2.052	1.153	2.052	1.153	1.981	1.163	2.009	1.163
	MeCN	2.051	1.153	2.052	1.153	1.976	1.165	2.004	1.165
	py	2.051	1.153	2.052	1.153	1.978	1.165	2.006	1.164
	BuCN	2.051	1.153	2.052	1.153	1.977	1.165	2.005	1.165

8 References

[1] M. J. Frisch, G. W. Trucks, H. B. Schlegel, G. E. Scuseria, M. A. Robb, J. R. Cheeseman, G. Scalmani, V. Barone, B. Mennucci, G. A. Petersson, H. Nakatsuji, M. Caricato, X. Li, H. P. Hratchian, A. F. Izmaylov, J. Bloino, G. Zheng, J. L. Sonnenberg, M. Hada, M. Ehara, K. Toyota, R. Fukuda, J. Hasegawa, M. Ishida, T. Nakajima, Y. Honda, O. Kitao, H. Nakai, T. Vreven, J. A. Montgomery, Jr., J. E. Peralta, F. Ogliaro, M. Bearpark, J. J. Heyd, E. Brothers, K. N. Kudin, V. N. Staroverov, T. Keith, R. Kobayashi, J. Normand, K. Raghavachari, A. Rendell, J. C. Burant, S. S. Iyengar, J. Tomasi, M. Cossi, N. Rega, J. M. Millam, M. Klene, J. E. Knox, J. B. Cross, V. Bakken, C. Adamo, J. Jaramillo, R. Gomperts, R. E. Stratmann, O. Yazyev, A. J. Austin, R. Cammi, C. Pomelli, J. W. Ochterski, R. L. Martin, K. Morokuma, V. G. Zakrzewski, G. A. Voth, P. Salvador, J. J. Dannenberg, S. Dapprich, A. D. Daniels, O. Farkas, J. B. Foresman, J. V. Ortiz, J. Cioslowski, D. J. Fox, Gaussian 09, Gaussian, Inc., Wallingfort, CT, **2013**.

[2] F. Furche, R. Ahlrichs, C. Hättig, W. Klopper, M. Sierka, F. Weigend, *Wiley Interdiscip. Rev. Comput. Mol. Sci.* **2014**, *4*, 91.

[3] *TURBOMOLE V7.4 2019, a development of University of Karlsruhe and Forschungszentrum Karlsruhe GmbH, 1989-2007, TURBOMOLE GmbH, since 2007; available from http://www.turbomole.com.*

[4] a) S. Grimme, J. Antony, S. Ehrlich, H. Krieg, *J. Chem. Phys.* **2010**, *132*, 154104; b) S. Grimme, S. Ehrlich, L. Goerigk, *J. Comput. Chem.* **2011**, *32*, 1456.

[5] P. Boden, P. Di Martino-Fumo, T. Bens, S. Steiger, U. Albold, G. Niedner-Schatteburg, M. Gerhards, B. Sarkar, *Chem. Eur. J.* **2021**, *27*, 12959–12964.
